# The Pink Box: Exclusive Homochiral Aromatic Stacking in a Bis-perylene Diimide Macrocycle

**DOI:** 10.1021/jacs.2c03531

**Published:** 2022-06-28

**Authors:** Samuel E. Penty, Martijn A. Zwijnenburg, Georgia R. F. Orton, Patrycja Stachelek, Robert Pal, Yujie Xie, Sarah L. Griffin, Timothy A. Barendt

**Affiliations:** † School of Chemistry, University of Birmingham, Edgbaston, Birmingham B15 2TT, United Kingdom; ‡ Department of Chemistry, University College London, 20 Gordon Street, London WC1H 0AJ, United Kingdom; § Department of Chemistry, University of Durham, South Road, Durham DH1 3LE, United Kingdom

## Abstract

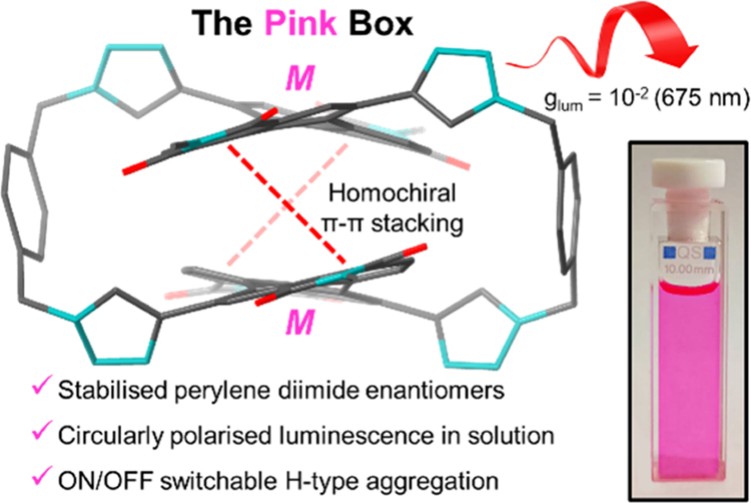

This work showcases chiral complementarity in aromatic stacking interactions as an effective tool to optimize the chiroptical and electrochemical properties of perylene diimides (PDIs). PDIs are a notable class of robust dye molecules and their rich photo- and electrochemistry and potential chirality make them ideal organic building blocks for chiral optoelectronic materials. By exploiting the new bay connectivity of twisted PDIs, a dynamic bis-PDI macrocycle (the “Pink Box”) is realized in which homochiral PDI–PDI π–π stacking interactions are switched on exclusively. Using a range of experimental and computational techniques, we uncover three important implications of the macrocycle’s chiral complementarity for PDI optoelectronics. First, the homochiral intramolecular π–π interactions anchor the twisted PDI units, yielding enantiomers with half-lives extended over 400-fold, from minutes to days (in solution) or years (in the solid state). Second, homochiral H-type aggregation affords the macrocycle red-shifted circularly polarized luminescence and one of the highest dissymmetry factors of any small organic molecule in solution (*g*_lum_ = 10^–2^ at 675 nm). Finally, excellent through-space PDI–PDI π-orbital overlap stabilizes PDI reduced states, akin to covalent functionalization with electron-withdrawing groups.

## Introduction

Since Pedersen’s pioneering work on crown ethers,^1^ macrocycles have been the workhorses of supramolecular chemistry. Their unique shape persistence and tunability have enabled the full tool kit of noncovalent interactions to be explored.^2^ Beyond molecular recognition,^3^ macrocycles are now being exploited in a diverse range of fields, including sensing,^4^ catalysis,^5^ and organic electronics,^6−8^ using architectures that contain multiple π-conjugated components.^9^ Here, the preorganization of aromatic groups to promote inter-^10^ or intramolecular^11^ π–π stacking interactions is key to tuning macrocycle properties. Connecting two viologen units by rigid spacers, the “Blue Box” is an archetypal aromatic-based macrocycle^12^ that has found numerous applications in functional organic materials^13^ and machines.^14^ Other common π-conjugated components for macrocycles include porphyrins,^15,16^ tetrathiafulvalenes,^17,18^ and rylene diimides.^19^ In the latter class, perylene diimides (PDIs) are important targets because they are robust,^20^ economic organic dyes^21^ with a readily functionalizable scaffold.^22^ Coupled with their renowned electron-accepting and photophysical properties, PDIs are ubiquitous organic building blocks for next-generation semiconductor and optoelectronic materials.^23−26^

**Figure 1:**
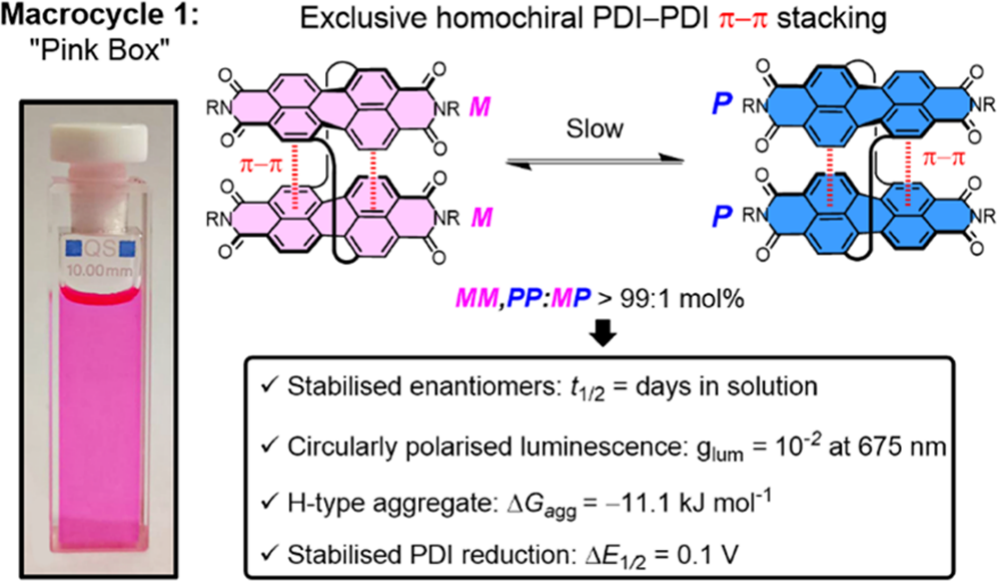
Exclusive homochiral π–π stacking in bis-PDI macrocycle **1**, the Pink Box.

Complementary π–π stacking interactions are critical to advancing the performance and diversifying the applications of PDI-based electronic materials, motivating their integration into preorganized macrocyclic architectures.^4,19,27−32^ Importantly, functionalization of the PDI bay positions (1, 6, 7, 12) imparts a propeller-type contortion of the perylene core, generating axial chirality (denoted ***M*** or ***P***).^33^ However, compared to π–π distance and relative orientation,^34,35^ axial chirality provides an underexploited handle for optimizing PDI–PDI π–π stacking interactions.^36,37^ In all but four bis-PDI macrocycles^27,38−40^ the dyes are connected *via* the imide positions, a strategy that necessitates the installation of up to four bulky groups in the bay region for solubility.^4,19,28,29,31,32^ However, we realized that these bay substituents can hamper homochiral PDI–PDI π–π stacking. This inspired us to exploit connectivity via two PDI bay positions (1, 7), realizing a new bis-PDI macrocycle (**1**), nicknamed the Pink Box due to its color (Figure 1). This macrocycle exhibits exclusive homochiral aromatic stacking between its PDI units, enhancing both chiroptical and electrochemical properties.

**Figure 2:**
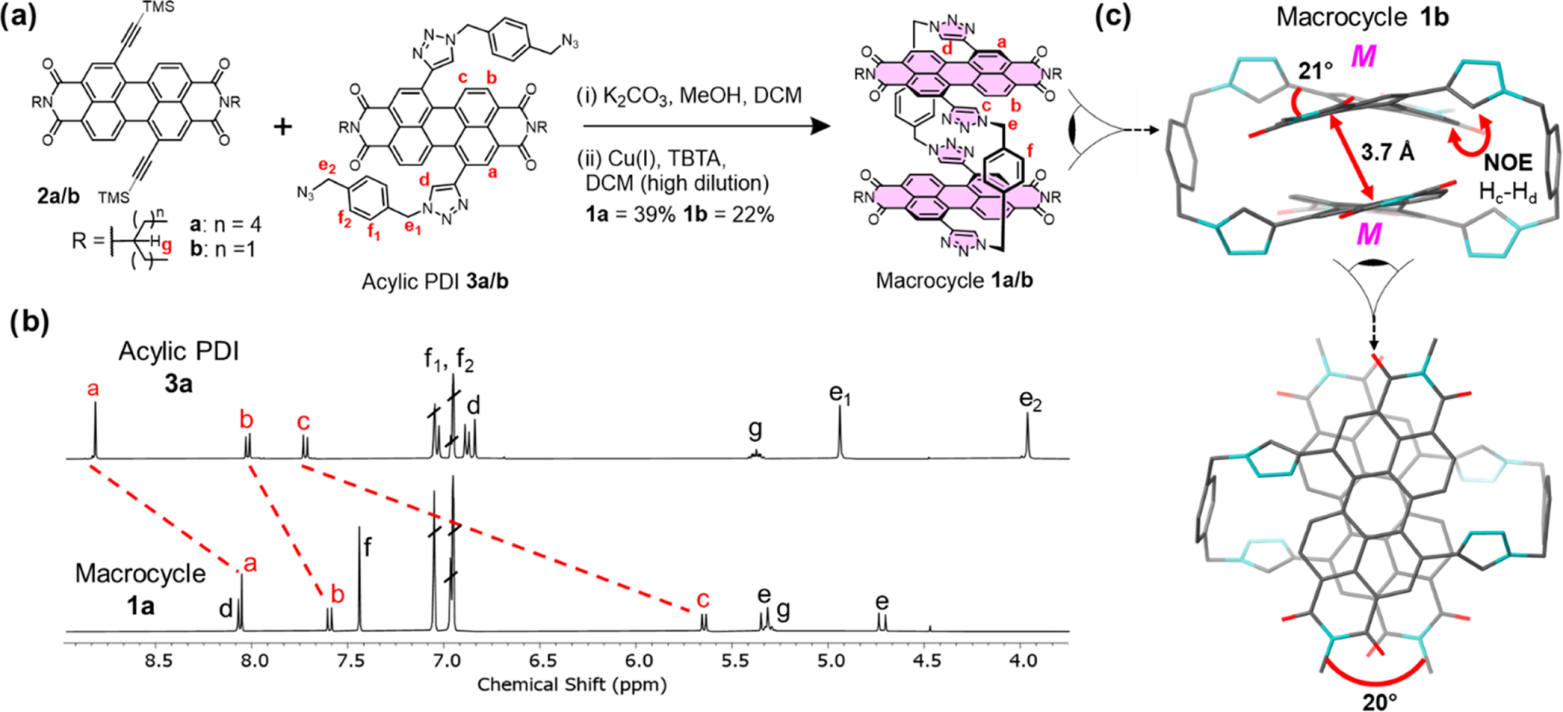
(a) Synthesis of bis-PDI macrocycles **1a/b**. (b) Comparison of the ^1^H NMR spectra of macrocycle **1a** and acyclic PDI **3a** (toluene-*d*_8_, 373 K, 400 MHz). (c) X-ray crystal structure of macrocycle **1b** (***MM***), viewed from the side and the top (alkyl side chains, hydrogens, and cocrystallized solvent molecules are omitted for clarity, see the Supporting Information, Section 3).

The linker choice is also key to our macrocycle design. The short aromatic spacers of **1** afford shape persistence and close PDI–PDI contacts (3.7 Å, Figure 2c), significantly closer than the two previous bay connected bis-PDI macrocycles (6 and 16 Å),^27,40^ which provides excellent electronic coupling. This is important because while the methylene groups afford some conformational flexibility, they prevent PDI–PDI through-bond conjugation.^40^ The crystal structure of macrocycle **1**, the first of a PDI–triazole derivative, reveals that bay heterocycles cause substantial twisting of the aromatic framework (dihedral angle = 21°, Figure 2c), giving rise to enantiomers ***MM*** and ***PP***, and the diastereomer ***MP***, which is a meso-isomer due to a mirror plane between the two PDIs.^40^ Previous bis-PDI macrocycles have shown that ***MM*** and ***PP*** interconvert rapidly at room temperature (*t*_1/2_ = seconds–minutes)^31,40−42^*via**MP***,^40^ hampering applications in chiral optoelectronics. Therefore, our aim was to exploit strong homochiral π–π stacking in macrocycle **1** to stabilize PDI enantiomers for functional chiroptical materials. Although bay and imide connectivity have been used simultaneously to prevent stereoisomer interconversion in a bis-PDI macrocycle,^43^ we report an alternative strategy that uses only bay connectivity, making the imide positions available for future potential modifications.

Herein, we show that complementary intramolecular π–π stacking interactions in macrocycle **1** (Figure 1) generate PDI enantiomers exclusively in solution (***MM***,***PP***:***MP*** > 99:1 mol %). While complete selectivity for homochiral recognition between axially chiral PDIs has been reported,^31,44^ this is unprecedented in solely bay connected bis-PDI macrocycles.^27,40^ Exclusive homochirality affords three important advances for PDI chiral optoelectronics. First, for chirality, it raises the free energy barrier of PDI stereoisomer interconversion, extending ***MM***/***PP*** half-lives more than 400-fold, from minutes to days, enabling their resolution. Second, for photophysics, it generates an intramolecular H-type chiral aggregate. This is key to realizing the highest circularly polarized luminescence dissymmetry factor of any discrete PDI in solution (*g*_lum_ = 10^–2^ at 675 nm vs *g*_lum_ = 10^–3^ at 655 nm),^45−49^ which is also one of the highest of any small organic molecule in solution (Table S3).^50−53^ Third, for PDI electrochemistry, excellent through-space π–π electronic communication stabilizes PDI reduced states, matching the influence of conventional electron-withdrawing groups.^34,54^

## Results and Discussion

### Synthesis and Characterization

The bis-PDI macrocycle **1a** was prepared using a multistage synthetic procedure, capitalizing on robust copper(I)-catalyzed azide–alkyne cycloaddition (CuAAC) “click” chemistry for the final macrocyclization step. Here, stoichiometric amounts of bis-alkyne PDI **2a** and bis-azide PDI **3a** were reacted under high-dilution conditions (0.3 mM), to favor macrocyclization (Figure 2a).^55^ The desired [1 + 1] macrocycle **1a** was isolated in 39% yield following purification by preparative silica thin layer chromatography, with key side products identified as the larger [2 + 2] and [3 + 3] macrocycles (Supporting Information, Section 1). This macrocyclization yield is over four times larger than previous bay connected bis-PDI macrocycles.^27,40^ Full synthetic procedures and compound characterization data are provided in the Supporting Information, Section 1.

Macrocycle **1a** was characterized using ^1^H and ^13^C NMR spectroscopy, which, alongside high-resolution mass spectrometry, confirmed a [1 + 1] macrocyclic product (Supporting Information, Section 1). The ^1^H NMR spectrum in toluene-*d*_8_ at room temperature was broad but dramatically sharpened at 100 °C (Figures 2b and S1), as found for a previous bay-strapped PDI macrocycle due to restricted rotations of the linkers and branched undecyl side chains side chains.^40^ Indeed, even at high temperature, the cyclic framework of **1a** is rigid enough to afford diastereotopic splitting of the methylene protons H_e_ in the *para*-xylyl linker^56^ not found for the acyclic bis-triazole PDI **3a** H_e1,e2_ (Figure 2b).

Further structural characterization of this new macrocycle architecture was provided by single-crystal X-ray diffraction (Supporting Information, Section 3). While our attempts to grow crystals of **1a** were unsuccessful, an identical macrocycle^57^ bearing shorter pentyl chains at the imide positions, **1b**, yielded purple needle-like crystals by slow diffusion of methanol into a chloroform solution, enabling single-crystal diffraction data to be collected at a synchrotron.^58^ The crystal structure of **1b** reveals a small relative rotation of the PDI units (20°), enabling close contacts between them (3.7 Å), characteristic of strong intramolecular π–π stacking (Figures 2c and S9),^59^ alongside CH_d_^...^O triazole–imide hydrogen bonding. This is in line with density functional theory predictions for the lowest energy conformer (Figures S42 and S43), including its predicted ^1^H NMR spectrum (Supporting Information, Section 9c), of a version of **1** with methyl groups at the imide position. The PDI–PDI interactions are maintained in toluene-*d*_8_ solution. This is because relative to acyclic PDI **3a**, the ^1^H NMR spectrum of macrocycle **1a** reveals large upfield shifts of aromatic protons H_a–c_, diagnostic of closely stacked π surfaces (Figure 2b), concomitant with a downfield shift of hydrogen-bonded H_d_.^60^ Furthermore, a new through-space NOE between PDI H_c_ and triazole H_d_ signals in **1a** (Figure S5) is in agreement with their proximity in the crystal structure (Figure 2c).

### PDI Chirality

The crystal structure of macrocycle **1b** reveals the axial chirality exhibited by each PDI unit, arising from its twisted aromatic framework (dihedral angle = 21°, Figure 2c). The intramolecular π–π stacking is complementary, occurring exclusively between PDIs of the same chirality, such that the unit cell contains only the enantiomers of the macrocycle ***MM*** and ***PP***.^61^ The diastereomer ***MP*** is not observed (Supporting Information, Section 3). Importantly, this is also the case in toluene-*d*_8_ solution because the ^1^H NMR spectrum of macrocycle **1a** contains only a single set of peaks for the enantiomers ***MM*** and ***PP*** at 373 K (Figures 2b and S3). Building on previous reports of a preference for PDI–PDI homochirality over heterochirality,^31,44^ the exclusive formation of PDI enantiomers in solution is unprecedented in solely bay connected bis-PDI macrocycles.^27,40^

**Figure 3:**
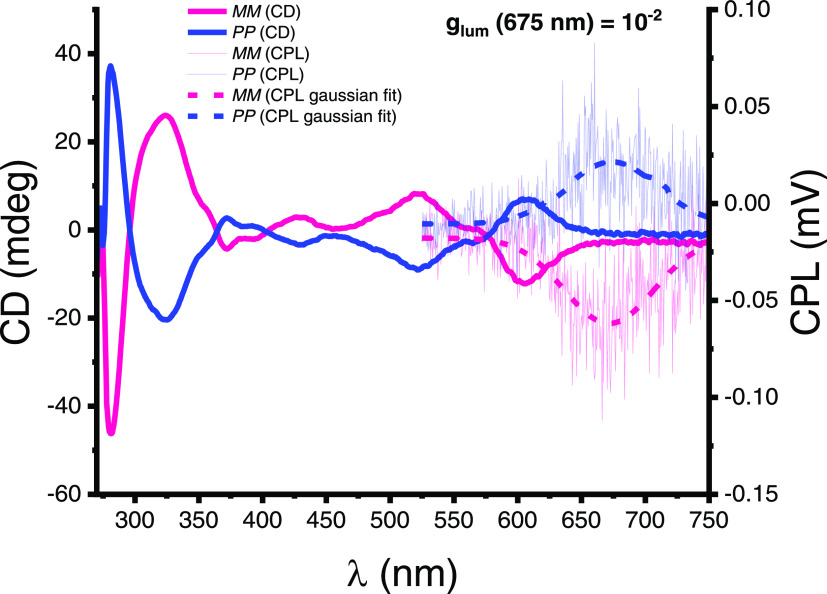
Circular dichroism (CD) and circularly polarized luminescence (CPL) spectra of macrocycle **1a** enantiomers ***MM*** (***MM*****:*****PP*** > 99:1 mol %) and ***PP*** (***MM*****:*****PP*** = 12:88 mol %) in solution (10 μM, toluene, 298 K, λ_ex_ = 520 nm, full experimental details are in the Supporting Information, Section 5). The enantiomers have been assigned by comparison to their theoretically predicted CD spectra (Supporting Information, Section 9).

Our next aim was to resolve the enantiomers of macrocycle **1a** by chiral high-performance liquid chromatography (HPLC) and characterize them using chiroptical techniques. With toluene as the major eluent, the chromatogram of **1a** contained two peaks of equal integration, corresponding to ***MM*** and ***PP*** (Figure S10). The identity of these enantiomers was confirmed by their opposite circular dichroism (CD) spectra (Figure 3), which is consistent with the single set of signals observed in the ^1^H NMR spectrum (Figure 2b) as well as the CD spectra of the two enantiomers of a methyl-capped version of **1** predicted by time-dependent density functional theory calculations in toluene (Table S13). We also measured the circularly polarized luminescence of enantiomers ***MM*** and ***PP*** in toluene (Figure 3 and Supporting Information, Section 5c). Excitingly, macrocycle **1a** exhibits one of the highest luminescence dissymmetry factors for a small organic molecule in solution^50−53^ (*g*_lum_ = 10^–2^, Table S3) and at wavelengths approaching the near-infrared (λ = 675 nm), useful for advanced security inks^62^ and multiphoton imaging.^63^

**Figure 4:**
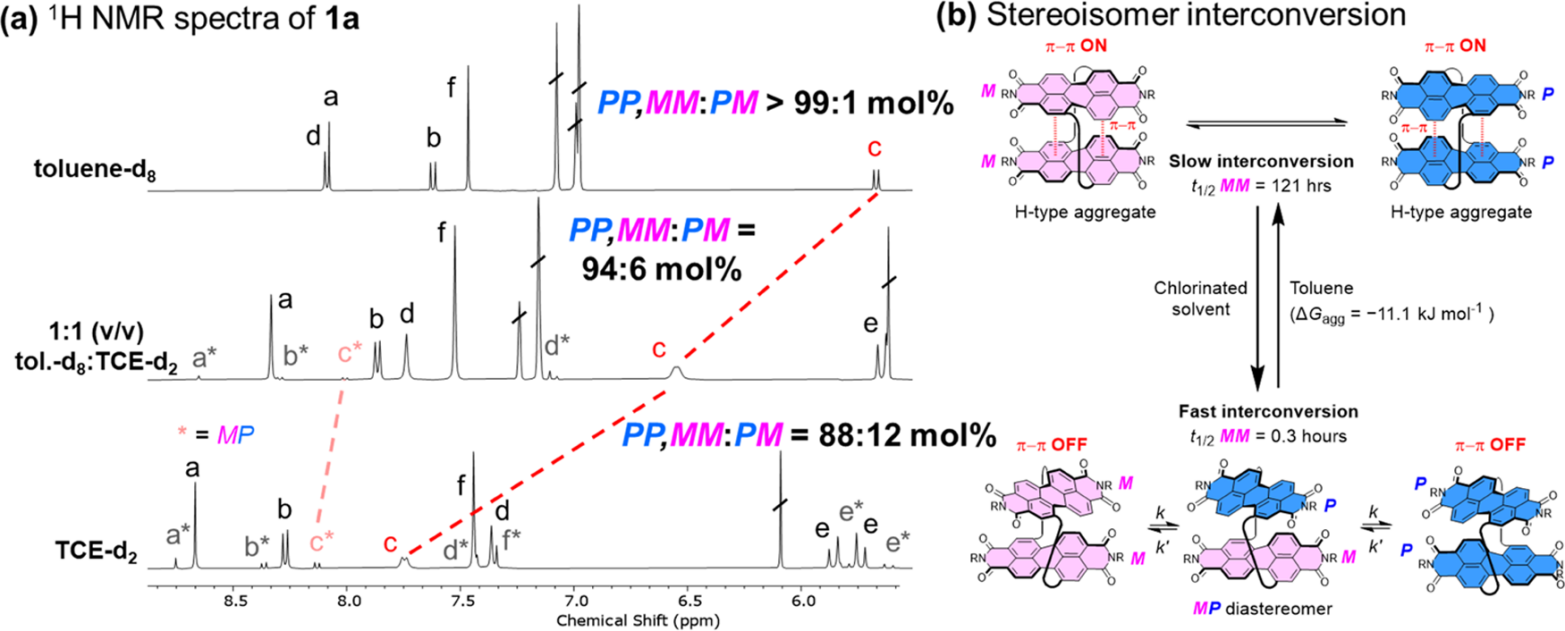
(a) Stacked ^1^H NMR spectra of macrocycle **1a** in different solvents (373 K, 400 MHz, each referenced to the same internal standard, full experimental details are in the Supporting Information, Section 2b). (b) Proposed solvent dependence of macrocycle conformations and stereoisomer interconversion.

We determined the thermodynamics and kinetics of macrocycle racemization in the solvents toluene and dichloromethane by measuring the time dependence of the circular dichroism spectrum decay of an enantiopure solution of **1a** (***MM***:***PP*** > 99:1 mol %) at 298 K (Table 1 and Supporting Information, Section 5b).^64^ Remarkably, the racemization rate in toluene is more than 400 times slower than in dichloromethane due to a significantly larger interconversion barrier (ΔΔ*G*^‡^ = 15 kJ mol^–1^, Figure 4b). Therefore, the ***MM***/***PP*** enantiomer half-life is increased from minutes in chlorinated solvent (*t*_1/2_ = 18 min) to days in toluene (*t*_1/2_ = 5 days), requiring nearly a month to racemize. To put this in context, the interconversion barrier of **1a** in toluene (Δ*G*^‡^ = 108 kJ mol^–1^) is significantly larger than previous dynamically chiral bis-PDI macrocycles employing imide- (53–69 kJ mol^–1^)^31,42^ or bay connectivity (86 kJ mol^–1^).^40^ Indeed, the barrier is the same as some tetra-*ortho*-substituted biaryls used for enantioselective catalysis and approaching that required for configurationally stable drugs (Δ*G*^‡^ = 114 kJ mol^–1^).^65−67^ Importantly for chiroptical materials, the enantiomers of **1a** are further stabilized in the solid state (*t*_1/2_ ∼ years, Figure S14). The racemization parameters were confirmed by time-course chiral HPLC measurements (Supporting Information, Section 4b).

**Table 1:** Kinetic and Thermodynamic Parameters for the Racemization of Macrocycle **1a**^a^

solvent	*t*_1/2_ (h)	Δ*G*^‡^ (kJ mol^–1^, 298 K)
dichloromethane	0.3 ± 0.01	93 ± 0.1
toluene	121 ± 10	108 ± 0.2

a Determined by CD spectroscopy using an enantiopure sample of **1a** (***MM:PP*** > 99:1 mol %). Full experimental details are in the Supporting Information, Section 5b.

To explain the striking difference in enantiomer stabilities, we used ^1^H NMR spectroscopy in different toluene-*d*_8_:1,1,2,2-tetrachloroethane-*d*_2_ (TCE-*d*_2_) ratios to identify the solvent dependence of macrocycle conformations (Figures 4a and S2).^68^ In contrast to toluene-*d*_8_, the chlorinated solvent disrupts PDI–PDI interactions since H_a–c_ are shifted downfield (Δδ = 0.7–2.1 ppm) and H_d_ upfield (0.8 ppm), giving a ^1^H NMR spectrum that more closely resembles the monomeric bis-triazole PDI **3a** (Figures 2b and 4a). The absence of the H_c_–H_d_ NOE for **1a** in TCE-*d*_2_ (Figure S4) also indicates a new conformation. Indeed, density functional theory calculations suggest new conformations may be adopted in dichloromethane due to their closer relative energies (Supporting Information, Section 9). While there are discrepancies in the energy landscape predicted in dichloromethane, most likely caused by the influence of intermolecular macrocycle–solvent hydrogen bonding not included in our model, there is good agreement between theoretical and experimental ^1^H NMR and UV–vis spectra (Supporting Information, Section 9b,c) for a distinct homochiral conformation of **1a** in a chlorinated solvent. Here, the PDI units are further rotated relative to one another (70°) than in the crystal structure (20°), which reduces their π–π interactions (Figures 4b and S44).^69^

Interestingly, the ^1^H NMR spectrum in TCE-*d*_2_ also reveals two sets of signals corresponding to two distinct isomers in an 8:1 ratio at 100 °C (Figure 4a), shown to be exchanging by ^1^H–^1^H EXSY NMR spectroscopy (Figure S4).^70,71^ In agreement with previous bis-PDI macrocycles,^31,40−42^ this strongly suggests the existence of the diastereomer ***MP***, alongside enantiomers ***MM*** and ***PP*** (Figure 4b). Upon increasing the proportion of toluene-*d*_8_, the mole fraction of the major component increases, and its ^1^H NMR signals converge on the single set of peaks observed in pure toluene-*d*_8_ (Figures 4a and S2), meaning high selectivity for the enantiomers over the diastereomer is maintained in chlorinated solvents (***MM,PP*****:*****MP*** = 88:12 mol % in TCE-*d*_2_ at 373 K). Importantly, the PDI–PDI π–π interactions are stronger in homochiral vs heterochiral conformations because ***MM***,***PP*** undergo the largest solvent-induced perturbations (Δδ = 2.1 ppm for H_c_, Figure 4a), while only small shifts are observed for corresponding ***MP*** signals (Δδ = 0.1 ppm for H_c*_).^60^

Using dichloromethane as the major eluent, the chromatogram of **1a** (Figure S11) contains two major peaks for each enantiomer, ***MM*** and ***PP***, alongside a minor third peak for ***MP*** (∼10 mol % at 298 K). While it was not possible to isolate the ***MP*** diastereomer by HPLC due to poor separation, we used ^1^H–^1^H EXSY NMR spectroscopy^70,71^ in TCE-*d*_2_ to show that the interconversion barrier (Δ*G*^‡^ = 95 kJ mol^–1^, 298 K) is close to that for racemization calculated by CD spectroscopy (93 kJ mol^–1^) and so consistent with the reported mechanism of racemization *via**MP*** (Figure 4b and Supporting Information, Section 2d).^40^ Indeed, the addition of toluene-*d*_8_ decreases the rate of ***MM***/***PP*** loss (Δ*k* = −0.02 s^–1^) and increases the rate of ***MP*** loss (Δ*k*′ = 0.49 s^–1^).

### PDI Photophysics

**Figure 5:**
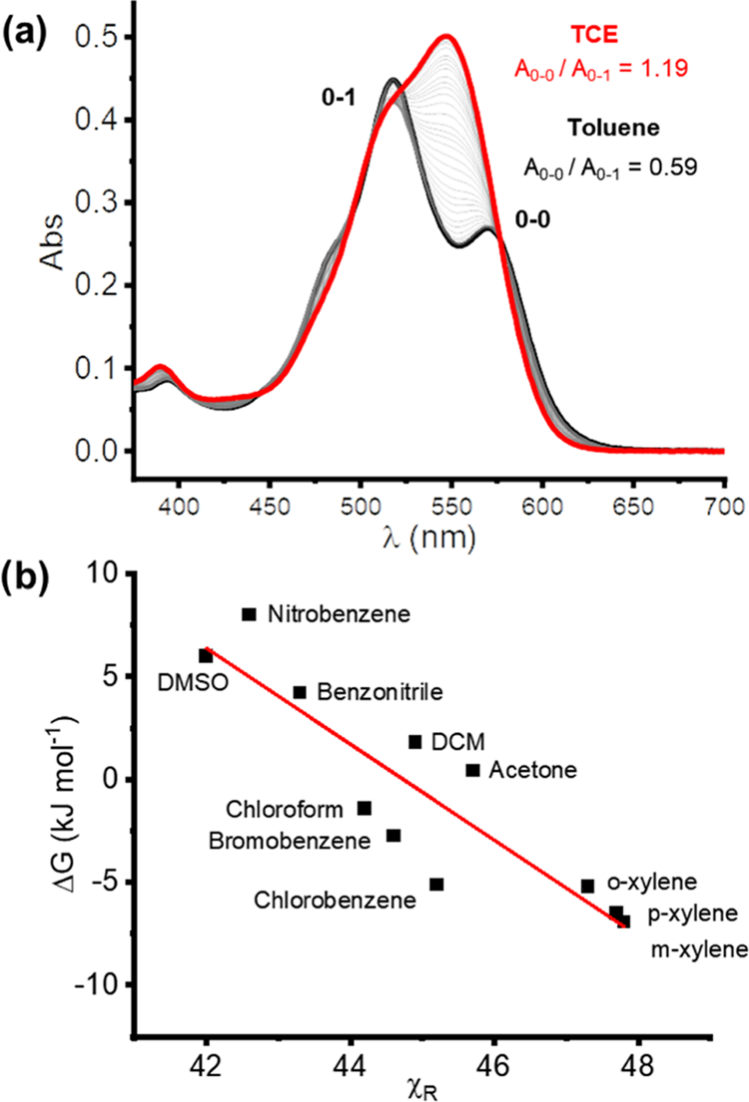
(a) UV–vis absorption spectra of macrocycle **1a** (10 μmol) measured in neat toluene (black), TCE (red), and intermediate toluene:TCE solvent ratios (gray). (b) Plot of Δ*G*_agg_ of **1a** vs the solvent scale χ*_r_* for a range of different solvents (Pearson’s *r* = 0.9).

UV–vis absorption and fluorescence emission spectroscopic studies provided further insight into the macrocycle’s switchable homochiral conformation (Figure 5a and Supporting Information, Section 6).^72^ Toluene induces the formation of an H-type aggregate in the ground state and a PDI excimer in the excited state of **1a**, while in TCE, the PDI–PDI electronic coupling is significantly weaker.^73^ The main PDI absorption band of **1a** in toluene has a 0–1 vibronic peak that is significantly larger than the 0–0 peak (*A*_0–0_/*A*_0–1_ = 0.58), characteristic of an H-type aggregate (Figures 5a and S23).^36^ Indeed, this *A*_0–0_/*A*_0–1_ ratio is one of the lowest of any PDI dimer,^28,30,74^ indicative of strong PDI–PDI electronic coupling in macrocycle **1a** and key to its high *g*_lum_, since the aggregation of chiral monomers is known to amplify their dissymmetry.^52,75^ This also implies that strong PDI–PDI π–π stacking is responsible for raising the barrier to macrocycle racemization. By contrast, the UV–vis spectrum of **1a** in TCE resembles that of the monomeric bis-triazole PDI **3a** (*A*_0–0_/*A*_0–1_ = 1.19, Figure 5a),^76^ consistent with the weakly coupled conformation in which H-type aggregation is disrupted (Figure 4b),^69^ suggested by density functional theory calculations in chlorinated solvents (Figure S44 and Supporting Information, Section 9).^72^ The UV–vis spectra of **1a** follow the Beer–Lambert law in both toluene and TCE, demonstrating that the solvent-mediated π–π stacking observed is intramolecular in origin (Figure S28). The fluorescence spectrum of macrocycle **1a** in toluene is indicative of a PDI–PDI excimer,^77^ since relative to **1a** in TCE and acyclic control **3a** (Figures S22 and S25), the emission becomes weaker (ΔΦ = 0.42), broader (Δ[FWHM] = 19 nm), and bathochromically shifted (Δλ = 20 nm), with a large Stokes shift (λ = 102 nm). The distinct UV–vis absorption spectra of macrocycle **1a** in chlorinated and aromatic solvents enabled us to estimate the strength of intramolecular PDI–PDI π–π interactions. The titration of TCE into a solution of **1a** in toluene (at constant concentration) led to the formation of isosbestic points (λ = 505, 523, 577 nm), indicative of conformations that are in equilibrium (Figures 5a and S37).^76^ Therefore, by applying the method of Moore and Ray,^78^ used for measuring the solvent-induced folding of aromatic oligomers, the free energy of intramolecular PDI–PDI H-type aggregation for **1a** in toluene is estimated to be Δ*G*_agg_ = −11.1 kJ mol^–1^ (Supporting information, Section 7). This is over twice as strong as the aggregation of PDIs functionalized with four bulky 4-*tert*-butylphenoxy groups at the bay positions, as reported using a recent PDI-based macrocycle in toluene.^79^

**Figure 6:**
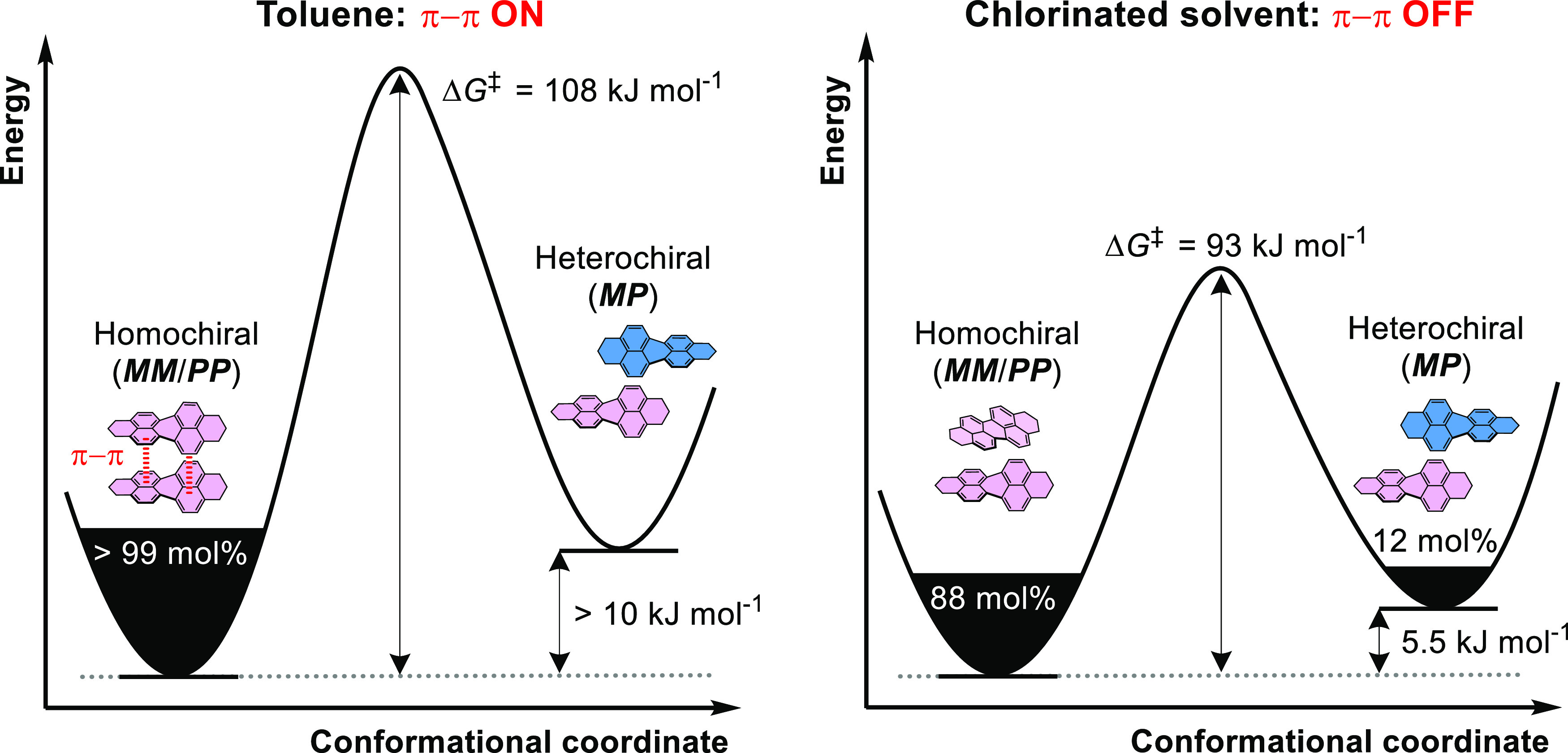
Proposed conformation–energy diagrams for macrocycle **1a** in toluene (left) and chlorinated solvent (right) using experimental data from CD spectroscopy and chiral HPLC. All values are at 298 K, except for the populations and free energy difference between ***MM***/***PP*** and ***MP***, which are estimated at 373 K, by ^1^H NMR spectroscopy.

The strong PDI–PDI π–π interactions in the H-type aggregate of **1a** help to explain the exclusive formation of enantiomers ***MM***,***PP*** and the larger Δ*G*^‡^ in toluene (Figure 6). Ideal H-type aggregation requires complementarity between twisted PDI π surfaces and so can only occur in a homochiral conformation. Indeed, density functional theory calculations showed the energy difference between ***MM/PP*** and ***MP*** decreases in dichloromethane relative to toluene (Supporting Information, Section 9), which will increase the diastereomer population. For bay connected bis-PDI macrocycles, enantiomer interconversion requires PDI units to somersault through the cavity^40^ and, for **1a**, this process will be inhibited by the strong intramolecular π–π stacking interactions in toluene.

We performed further UV–vis spectroscopy to understand the important role of solvent on PDI–PDI π–π interactions in macrocycle **1a** (Table S5). By correlating Δ*G*_agg_ with a range of solvent scales, we determined that solvent polarity and polarizability (e.g., the χ*_r_* scale. Figure 5b)^80^ are key parameters in dictating their strength while hydrogen bonding is not (Figures S29–S36). Therefore, polar and polarizable chlorinated solvents disrupt the macrocycle’s intramolecular π–π stacking interactions and H-type aggregation because, in contrast to toluene, these solvents better solubilize polycyclic aromatic hydrocarbons.^81^ This solvent dependence is analogous to that observed in the supramolecular self-assembly of PDI monomers, since both use π–π stacking interactions.^82^ Molecular recognition studies between **1a** and a range of polycyclic aromatic hydrocarbons revealed no evidence for guest binding, most likely due to the macrocycle’s small cavity, apparent from the crystal structure (Figure 2c).^83^

### PDI Electrochemistry

**Figure 7:**
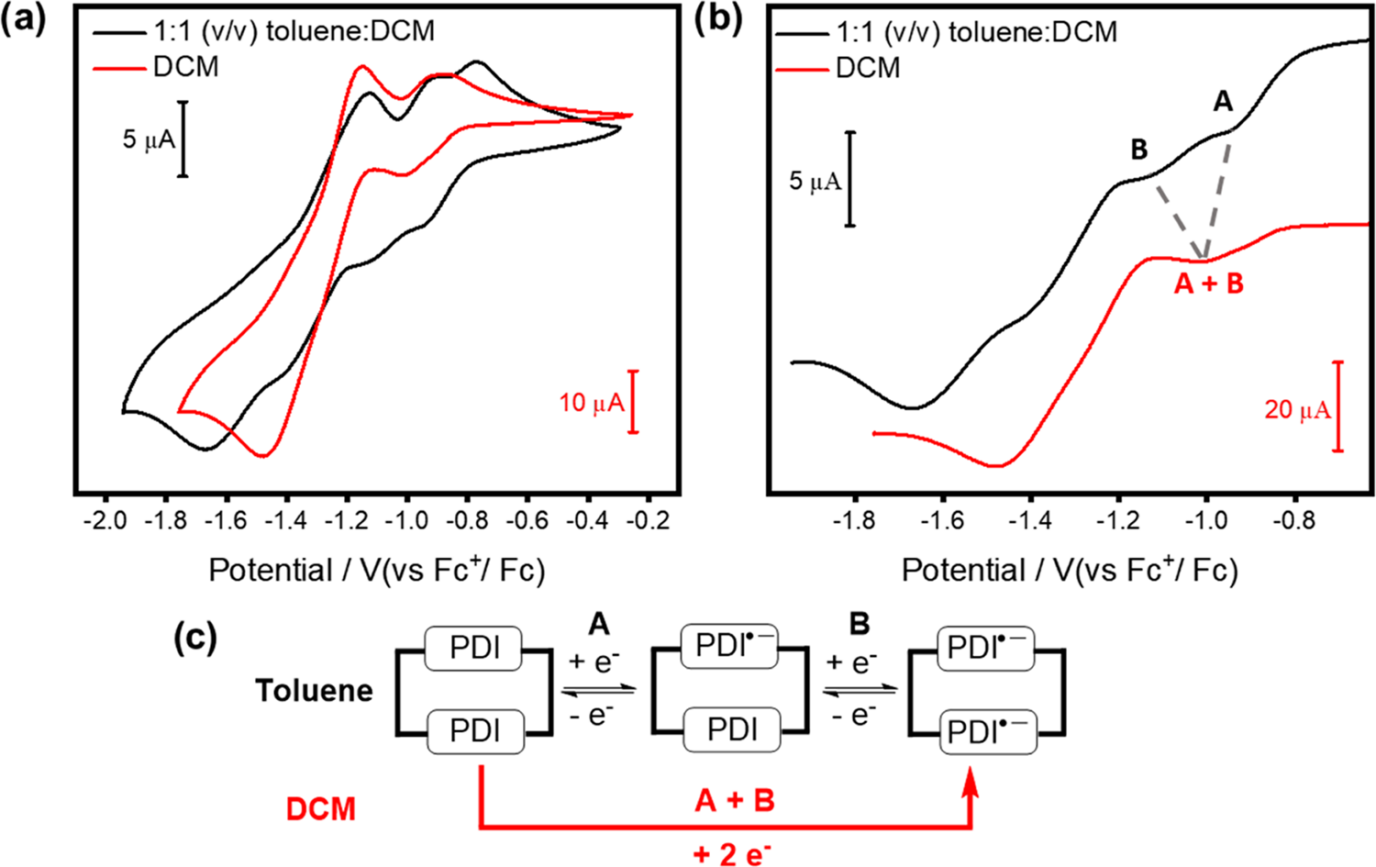
(a) Full and (b) zoomed-in region of the cyclic voltammograms of macrocycle **1a** recorded in dichloromethane and 1:1 (v/v) toluene:dichloromethane (each containing 0.4 M [^*n*^Bu_4_N][BF_4_], full experimental details are provided in the Supporting Information, Section 8). (c) Proposed schematic of the initial redox processes of macrocycle **1a** in each solvent.

The macrocycle’s solvent-dependent conformation is notable for providing switchable electrochemical properties (Figure 7 and Supporting Information, Section 8). In dichloromethane, cyclic voltammetry of **1a** revealed two typical chemically reversible two-electron PDI reduction processes.^36,84^ However, the addition of toluene splits the first peak into two distinct one-electron reductions, A and B (Figure 7b and Table S6).^85^ This demonstrates that homochiral intramolecular π–π stacking between PDI units facilitates their strong through-space electronic communication,^28,86^ which is switchable using a solvent. The H-type aggregate in 1:1 (v/v) toluene/dichloromethane^87^ also significantly stabilizes the first reduced PDI state (**1a**^•–^, *E*_1/2_ = −0.84 V) relative to the corresponding species in neat dichloromethane or the acyclic PDI control **3a**^•–^ (*E*_1/2_ = −0.94 V for both, Table S6).^88^ To put this in context, the enhancement in electron-accepting power of a PDI unit arising from its H-type homochiral π–π stacking is superior to the covalent addition of two *ortho*-cyanophenyl electron-withdrawing groups to the perylene core.^54^ Indeed, the electron deficiency of macrocycle **1a** (*E*_1/2_ = −0.84 V) is in line with a tetrachloro-substituted PDI (*E*_1/2_ = −0.87 V),^34^ demonstrating its potential as an n-type semiconducting material.

## Summary and Conclusions

In this work, we enhance the through-space π–π electronic communication of two PDI units by tuning their relative chirality (homochiral), orientation (H-type aggregate), and distance (3.7 Å) using a macrocycle. We have developed a novel bis-PDI macrocycle (**1**), in which complementary π–π interactions optimize the PDI’s chiroptical and electrochemical properties, demonstrated using a range of experimental and computational techniques.

Macrocycle **1** is unique among solely bay connected bis-PDI macrocycles^27,40^ in exhibiting homochiral π–π stacking exclusively in solution (***MM***,***PP*****:*****MP*** > 99:1 mol % in toluene). The closest analogue exhibits a 72% diastereomeric excess of its homochiral stereoisomers.^40^ An intramolecular H-type aggregate with strong π–π interactions (Δ*G*_agg_ = −11.1 kJ mol^–1^) is key to the chiral complementarity in **1** and arises from a short PDI–PDI separation (3.7 Å), shorter than other bay connected bis-PDI macrocycles.^27,40^ Homochiral aromatic stacking interactions anchor the PDI units of **1**, slowing the racemization rate by >400-fold and increasing the enantiomer half-life from minutes in chlorinated solvents to days in toluene and years in the solid state. As such, stereoisomer interconversion is significantly slower than previous dynamically chiral bis-PDI macrocycles (*t*_1/2_ = days vs minutes),^31,40−42^ with a barrier nearing that required for configurational locking.^43^ The enantiomers of **1** exhibit the highest circularly polarized luminescence dissymmetry factor of any discrete PDI in solution (*g*_lum_ = 10^–2^ vs 10^–3^).^45^ Indeed, in comparison to other small organic molecules in solution, the circularly polarized luminescence of **1** is the most red-shifted and has one of the highest dissymmetry factors (Table S3).^50−53^ The PDI electron-accepting ability of **1** is also elevated (Δ*E*_1/2_ = 0.1 V) and matches that of a tetrachloro-substituted PDI semiconducting material^34^ but with a notably higher barrier to stereoisomer interconversion (Δ*G*^‡^ = 108 vs 97 kJ mol^–1^).^33^ Therefore, this work demonstrates that the ordering of dynamic chiral PDIs using macrocyclic scaffolds can elevate their key chiroptical and electrochemical properties. Efforts to assemble multiple chiral PDIs using supramolecular chemistry are continuing in our laboratory.

## Supporting Information Available

Experimental procedures, NMR spectroscopy, X-ray crystallography, HPLC, CD, CPL, photophysics, cyclic voltammetry and DFT (PDF)
Relevant DFT optimized structures (ZIP)
