# Supplementary material for: The Pink Box: Exclusive Homochiral Aromatic Stacking in a Bis-perylene Diimide Macrocycle
Source: J Am Chem Soc. 2022 Jun 28;144(27):12290–8. doi: 10.1021/jacs.2c03531 (PMC9348826; doi:10.1021/jacs.2c03531)
Supplement: Supplementary file 1 — ja2c03531_si_001.pdf [file ja2c03531_si_001.pdf]

The Pink Box: Exclusive Homochiral Aromatic Stacking in a Bis-Perylene Diimide Macrocycle

Samuel E. Penty,[†] Martijn A. Zwijnenburg,[‡] Georgia R. F. Orton,[†] Patrycja Stachelek,[§] Robert Pal,[§] Yujie Xie,[†] Sarah L. Griffin,[†] Timothy A. Barendt*,[†]

[†]School of Chemistry, University of Birmingham, Edgbaston, Birmingham, B15 2TT, United Kingdom.
[‡]Department of Chemistry, University College London, 20 Gordon Street, London WC1H 0AJ, United Kingdom.
[§]Department of Chemistry, University of Durham, South Road, Durham, DH1 3LE, United Kingdom.
*t.a.barendt@bham.ac.uk

Supporting Information

Contents:

1. Synthesis and Characterisation S2
2. Further NMR spectroscopy experiments S15
3. X-ray crystallography S21
4. Chiral HPLC S23
5. Chiroptical studies S28
6. Photophysics S35
7. Quantification of intramolecular H-type aggregation S41
8. Electrochemistry S50
9. Density Functional Theory Calculations S52
10. References S58

1. Synthesis and Characterisation

All commercial solvents and reagents were used as purchased, unless otherwise stated. Anhydrous solvents were degassed with N_2 and dried using an Innovative Technology PureSolv MD 5 solvent purification system. $Cu(MeCN)_4{\cdot}PF_6$ was stored in a desiccator. TBTA was prepared following a literature procedure.[1] Water was distilled and microfiltered using an ELGA DV 35 Purelab water purification system. Chromatography was undertaken using silica gel (particle size: 40-63 μm) or preparative TLC plates (20 × 20 cm, 1 cm silica thickness).

^{1}H and ^{13}C NMR spectra were recorded using Bruker AVIII400 (400 MHz), Bruker AV NEO 400 (400 MHz) Bruker AV NEO 500 (500 MHz, with cryoprobe). Mass spectra were recorded using a Bruker UltrafleXtreme MALDI-TOF mass spectrometer or a Waters Synapt G2-S mass spectrometer for high resolution MS-ESI.

Details of equipment used for other analytical techniques (photophysics, electrochemistry, HPLC etc.) are provided in their appropriate sections in this supporting information. All analytical experiments were performed with bis-perylene diimide (PDI) macrocycle **1a** and acyclic bis-triazole PDI control **3a**. Only single crystal X-ray diffraction was performed with macrocycle **1b**, for which shorter alkyl chains enabled the growth of useable crystals. Apart from their side chains, macrocycles **1a** and **1b** are structurally identical, as evidenced by their matching ^{1}H NMR (aromatic signals, see below) and UV-vis spectra (**Figure S23** and **S24**).

Synthesis of macrocycles 1a and 1b

The synthesis of macrocycles **1a** and **1b** was carried out according to Scheme S1. Compounds **4b**[2] and **2a**[3] were prepared following literature procedures. Compounds **2a/b**, **3a/b** and **4a/b** were isolated as a mixture of 1,6 and 1,7 PDI regioisomers. Both macrocycles **1a** and **1b** were isolated as the pure 1,7 regioisomer since removal of the 1,6 regioisomer was possible by silica gel column chromatography at this stage.

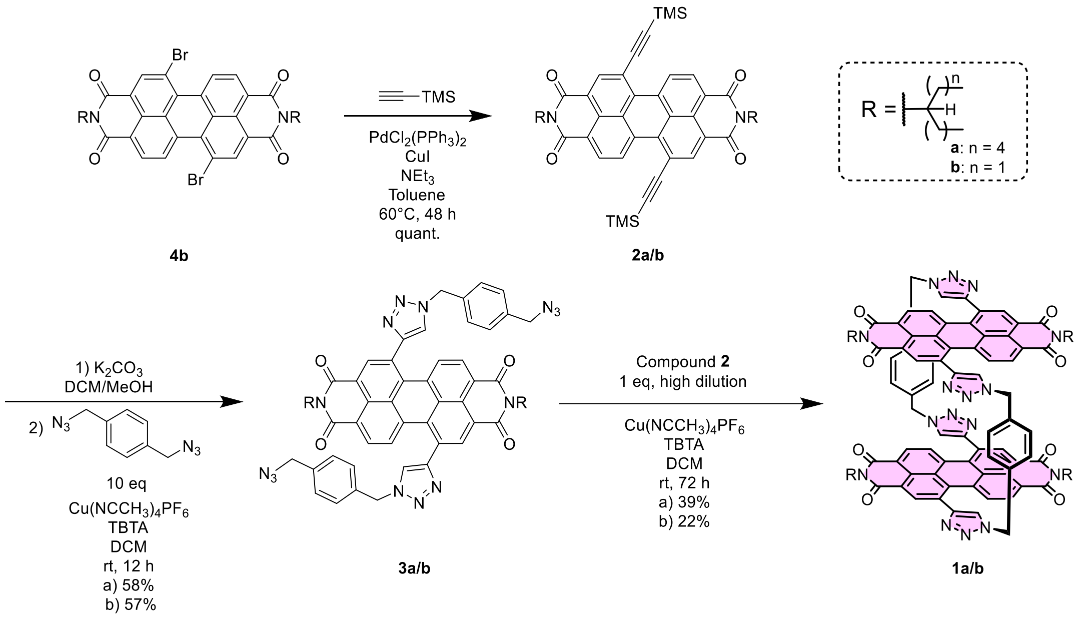

Scheme S1: Multistep synthesis of bis-PDI macrocycles**1a** and **1b**.

Acyclic bis-triazole PDI 3a

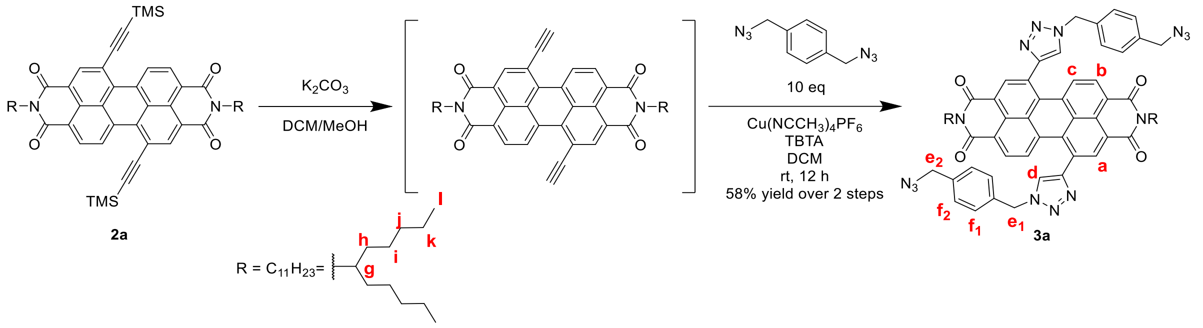

To a solution of TMS-protected bisalkyne PDI **2a**[2] (150 mg, 168 µmol) in DCM (20 ml) was added K_2CO_3 (85 mg, 616 µmol, 3.6 equiv) in MeOH (10 ml). The mixture was stirred at rt for 3 min, and completion of the reaction was confirmed by TLC. A further 20 mL of DCM was added to the mixture. The mixture was then washed with 1 M HCl (2 x 30 mL), water (2 x 30 mL) and brine (30 ml). The mixture was then dried over anhydrous $MgSO_4$ and concentrated to dryness *in vacuo* to afford the PDI bis-alkyne, which was used immediately without further purification. To a solution of this PDI bis-alkyne in dry DCM (20 ml) was added 1,4-bis(azidomethyl)benzene (316 mg, 1.68 mmol, 10 equiv) and Tris((1-benzyl-4-triazolyl)methyl)amine (TBTA) (17.86 mg, 34 µmol, 0.2 equiv). The solution was then de-gassed with argon. The copper (I) catalyst $Cu(CH_3CN)_4PF_6$ (12.5 mg, 34 µmol, 0.2 equiv) was then added and the solution was once again de-gassed with argon. The reaction was stirred at rt for 12 h. The solvent was then removed *in vacuo*. The resulting residue was purified by silica gel flash column chromatography (1:99 MeOH-DCM) affording the title compound as a purple solid (110 mg, 98 µmol, 58%).

^{1}H NMR (500 MHz, Chloroform-*d*, 1,7-regiosiomer) δ 8.63 (d, J = 26.4 Hz, $2H_b$), 8.12 (s, $2H_a$), 7.83 (d, J = 8.1 Hz, $2H_c$), 7.66 (s, $2H_d$), 7.43 – 7.36 (m, $8H_f$), 5.67 (s, $2H_{e1}$), 5.15 (p, $2H_g$), 4.42 (s, $4H_{e2}$), 2.27 – 2.16 (m, $4H_h$), 1.87 – 1.77 (m, $4H_h$), 1.43 – 1.15 (m, $24H_{i-k}$), 0.89 – 0.78 (m, $12H_l$).

^{13}C NMR (126 MHz, $CDCl_3$) δ 164.57, 163.45, 148.63, 136.88, 135.46, 134.60, 133.54, 130.37, 129.65, 129.25, 129.10, 128.96, 128.66, 128.62, 128.55, 123.30, 123.18, 122.61, 122.44, 121.96, 54.40, 54.37, 32.43, 31.88, 26.70, 22.73, 22.70, 14.19.

HRMS ESI (m/z) calculated for $C_{66}H_{71}N_{14}O_4^+$ $[M+H]^+$ 1123.5782, found 1123.5765.

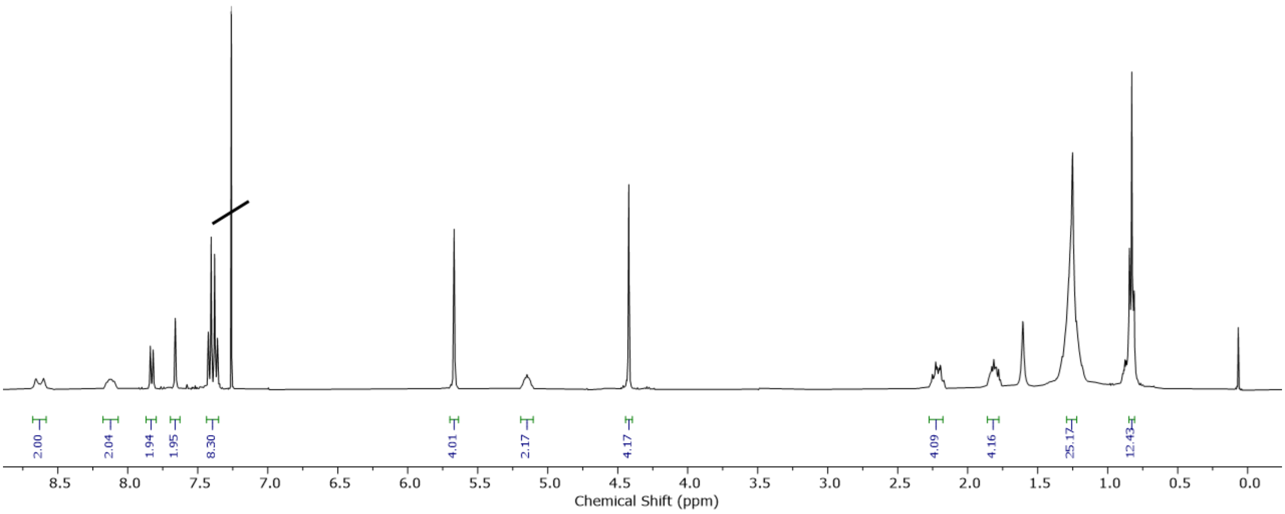

^1H NMR spectrum of **3a** (Chloroform-*d*, 298 K, 500 MHz).

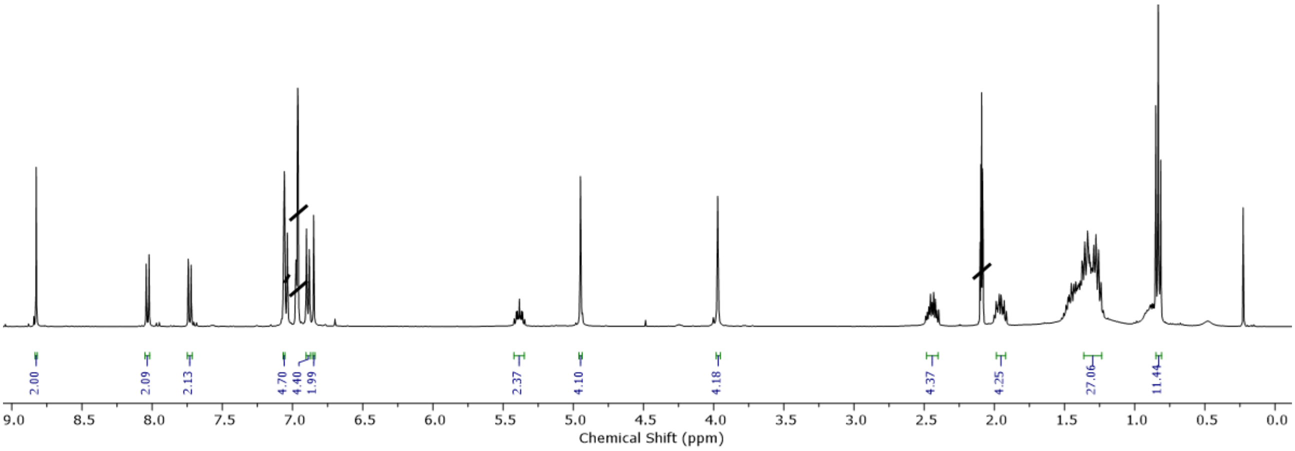

^1H NMR spectrum of **3a** (Toluene-d_8, 298 K, 500 MHz).

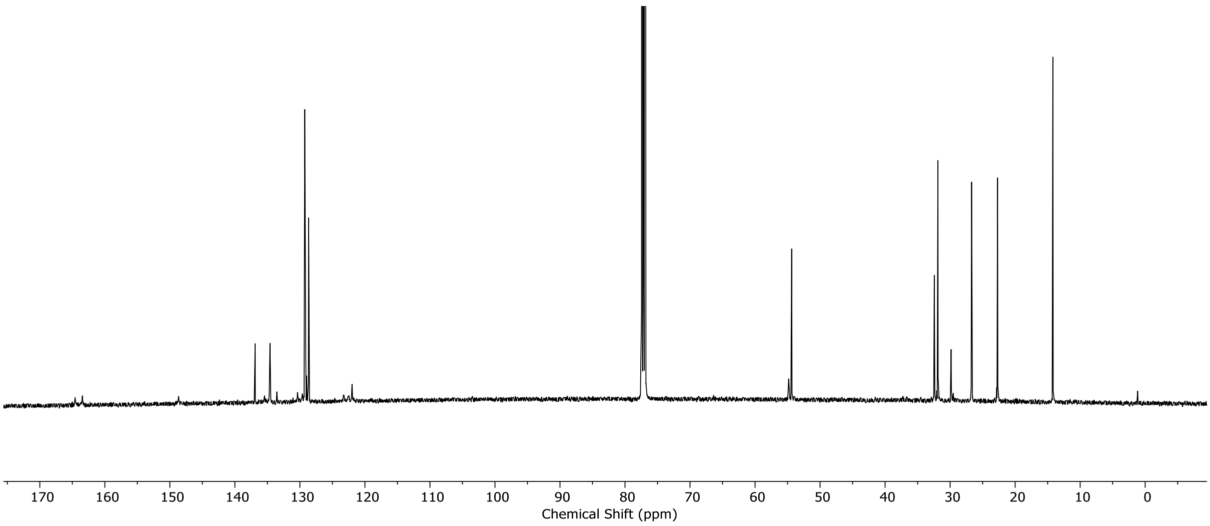

^{13}C NMR spectrum of **3a** (Chloroform-*d*, 298 K, 101 MHz).

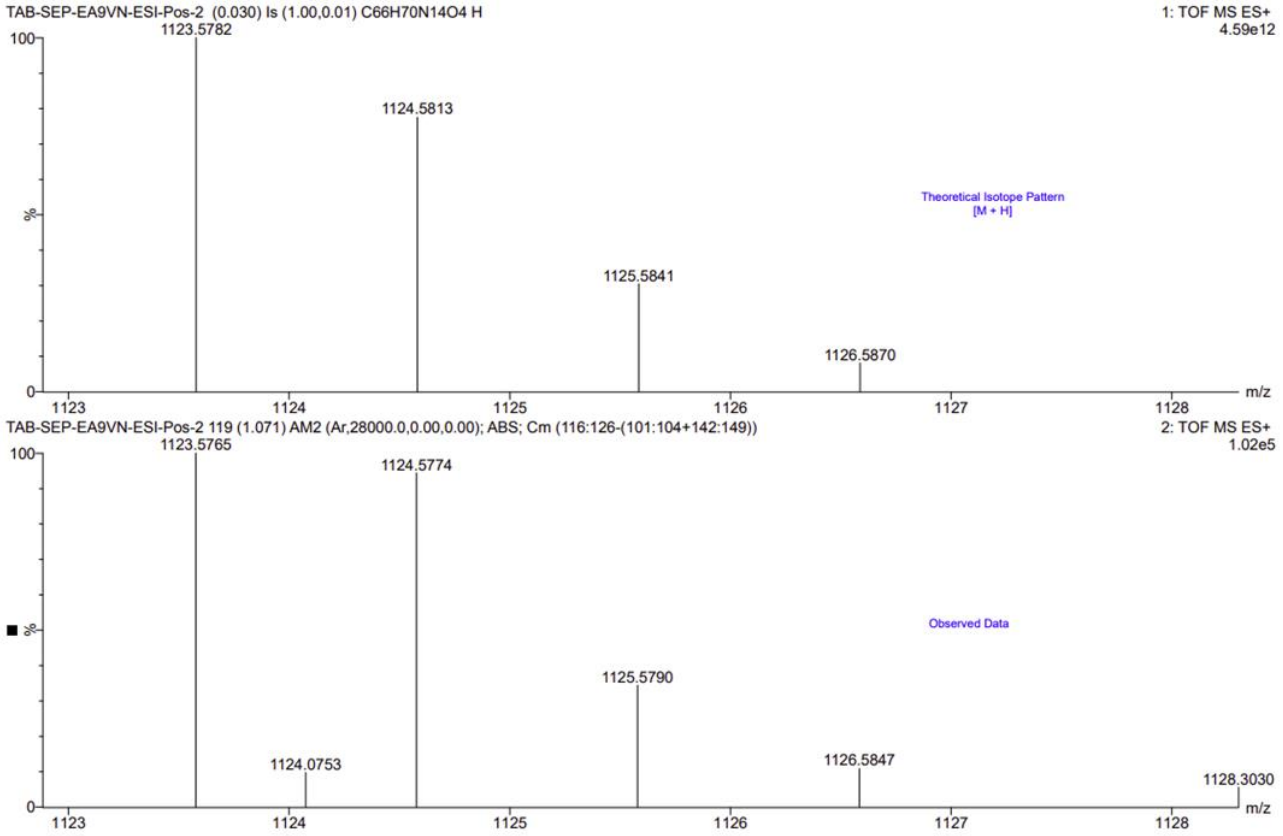

Calculated (top) and observed (bottom) ESI MS data for compound **3a**.

Bis-PDI macrocycle 1a

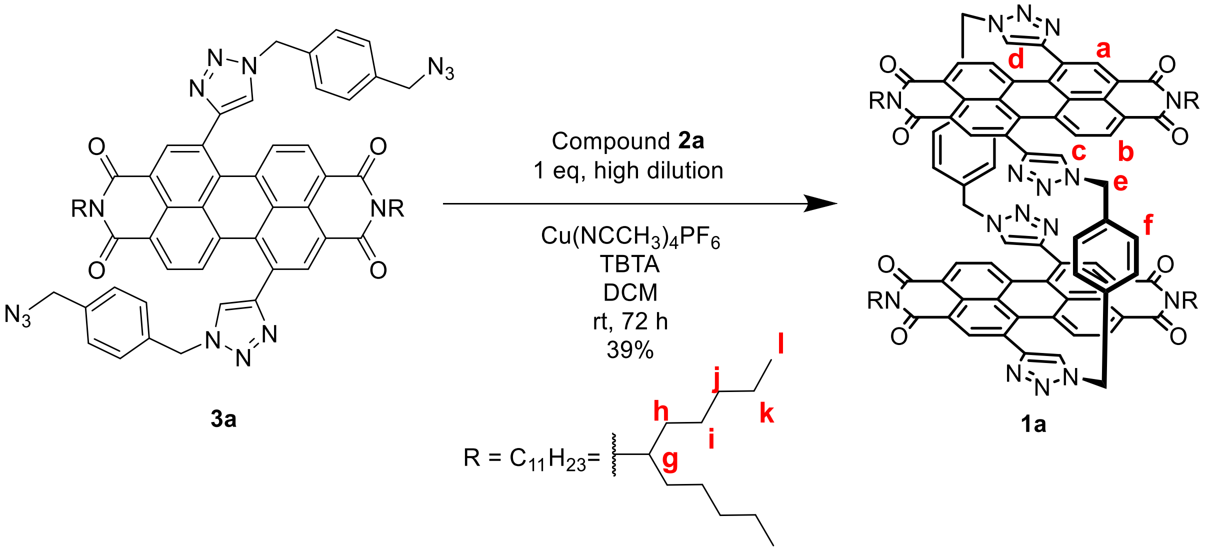

To a solution of compound **3a** (110 mg, 98 µmol) in DCM (250 mL) was added compound **2a** (73 mg, 98 µmol, 1 equiv) and Tris((1-benzyl-4-triazolyl)methyl)amine (TBTA) (21mg, 39 µmol, 0.4 equiv). The solution was then de-gassed with argon. The copper catalyst $Cu(CH_3CN)_4PF_6$ (14 mg, 39 µmol, 0.4 equiv) was then added and the solution was once again de-gassed with argon. The reaction was stirred at rt for 36 h and monitored by TLC (1:99 MeOH-DCM). The solvent was then removed *in vacuo*. The resulting residue was purified by silica gel flash

column chromatography (1:99 MeOH-DCM) followed by preparative silica TLC (0.5:99.5 MeOH-DCM), affording the title compound (as the pure 1,7-regioisomer) as a purple solid (70.8 mg, 38 µmol, 39%).

^{1}H NMR (400 MHz, TCE-*d*$_2$, 373K, the major species are the enantiomers, i.e. ***MM***,***PP:PM*** = 88:12 mol%) δ 8.58 (s, 4H$_a$), 8.18 (d, *J* = 8.1 Hz, 4H$_b$), 7.66 (d, *J* = 8.1 Hz, 4H$_c$), 7.35 (s, 8H$_f$), 7.27 (s, 4H$_d$), 5.77 (d, *J* = 15.2 Hz, 4H$_e$), 5.65 (d, *J* = 15.1 Hz, 4H$_e$), 5.10 (p, *J* = 8.4, 6.3 Hz, 4H$_g$), 2.27 – 2.06 (m, 8H$_h$), 2.05 – 1.83 (m, 8H$_h$), 1.32 – 1.24 (m, 48H$_{i-k}$), 0.93 – 0.83 (m, 24H$_l$).

^{13}C NMR (101 MHz, TCE, 373K) δ 163.48, 162.81, 148.02, 136.10, 134.87, 134.34, 133.53, 132.59, 129.43, 128.98, 128.27, 128.12, 127.76, 122.74, 121.01, 54.90, 53.84, 31.36, 29.40, 26.45, 22.18, 13.62.

HRMS (**ESI**) (m/z) calculated for $C_{116}H_{125}N_{16}O_8^+$ $[M+H]^+$ 1869.9866, found 1869.9879.

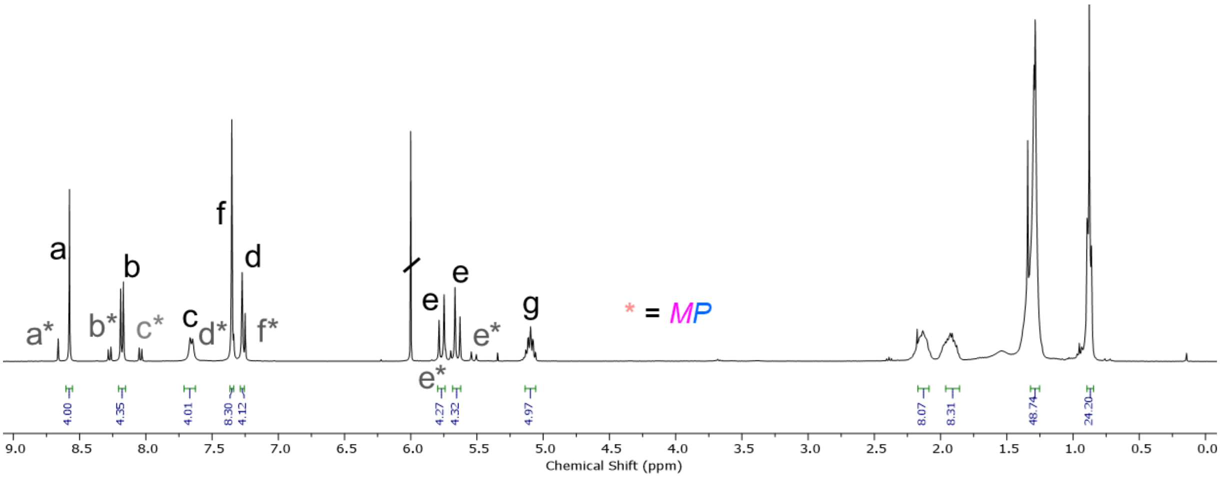

^{1}H NMR spectrum of **1a** (TCE-*d*$_2$, 373K, 400 MHz). Peaks are assigned in the 5-9 ppm region. Peaks labelled with an asterisk correspond to the minor species (***MP*** diastereomer), while those without an asterisk correspond to the major species (***MM***/***PP*** enantiomers).

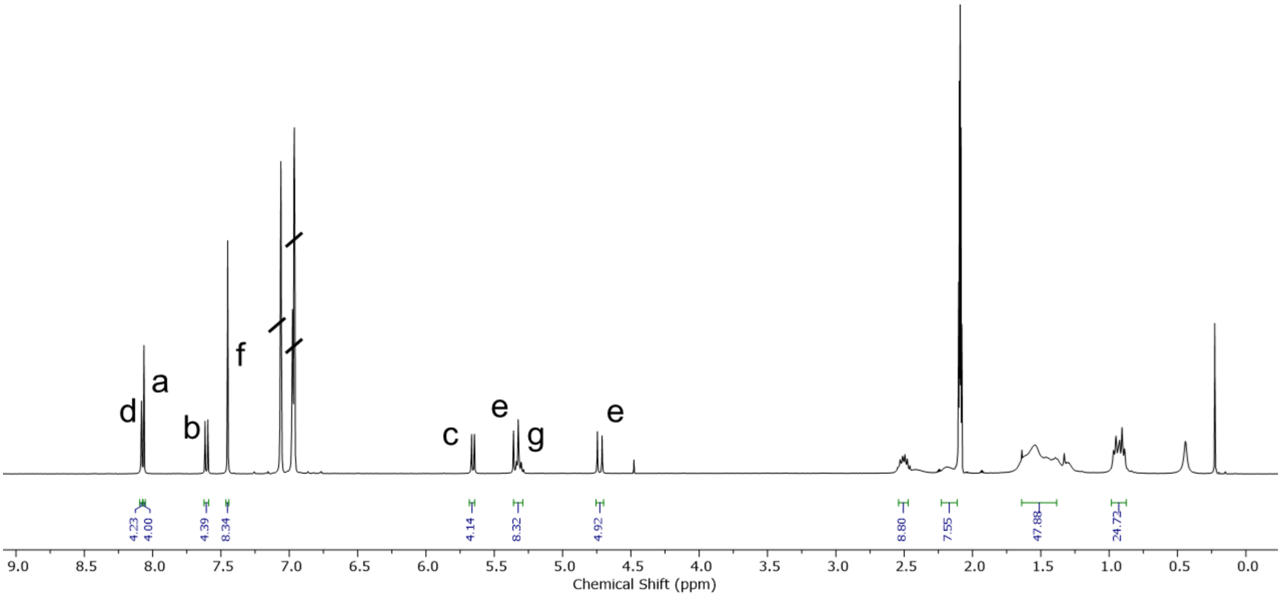

^{1}H NMR spectrum of **1a** (toluene-*d*$_8$, 373 K, 400 MHz, ***MM,PP:PM*** **> 99:1 mol%**).

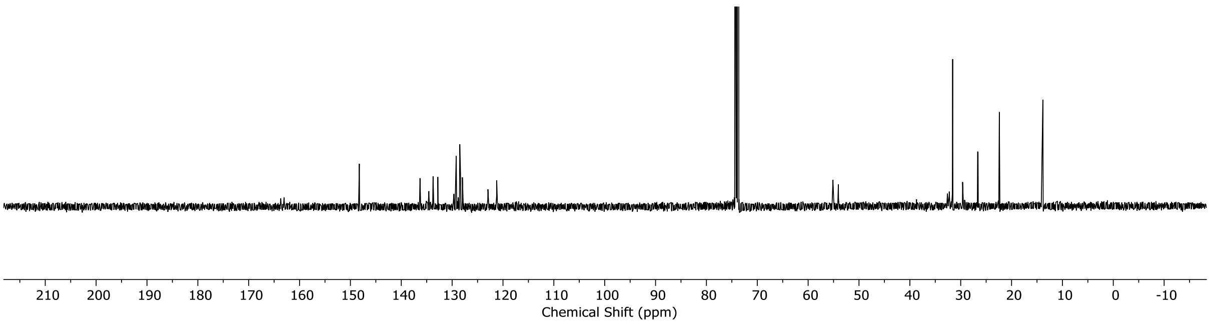

^{13}C NMR spectrum of **1a** (TCE-d_2, 373 K, 101 MHz).

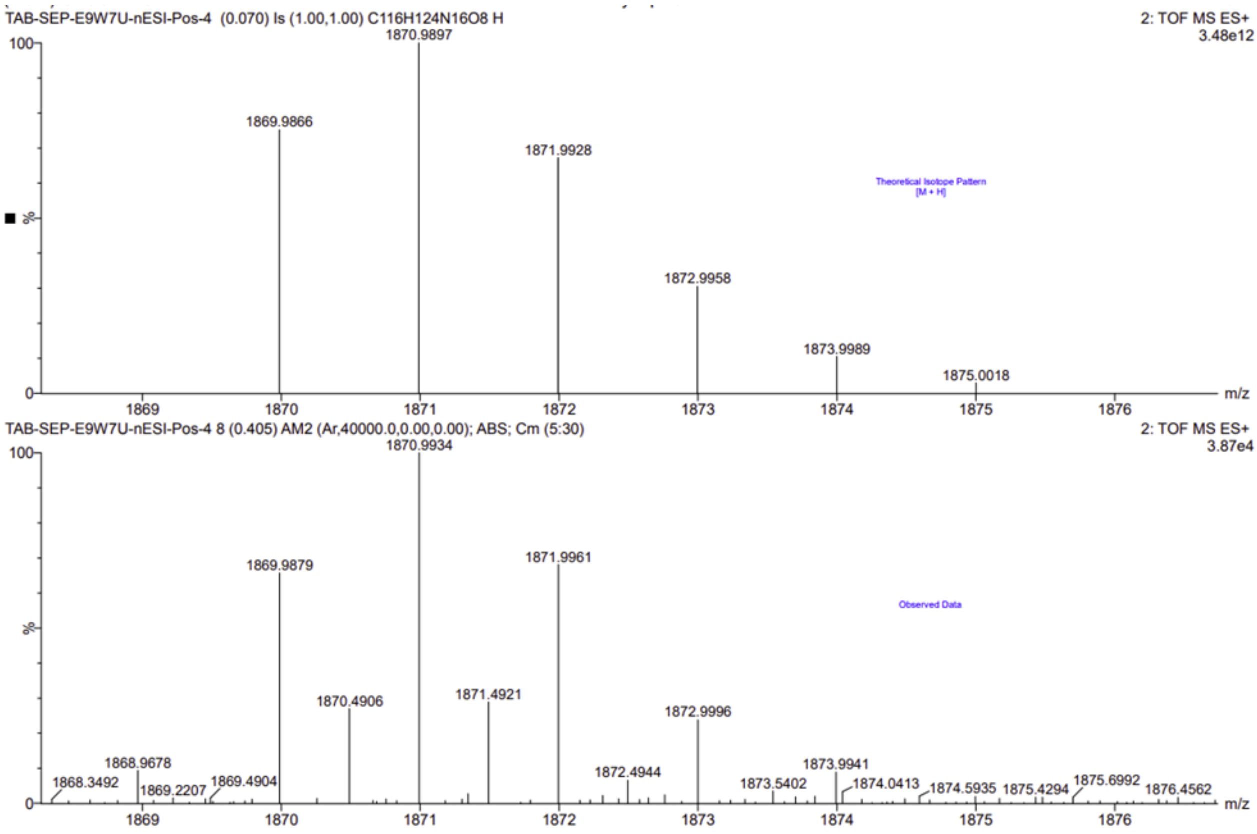

Calculated (top) and observed (bottom) ESI MS data for compound **1a**.

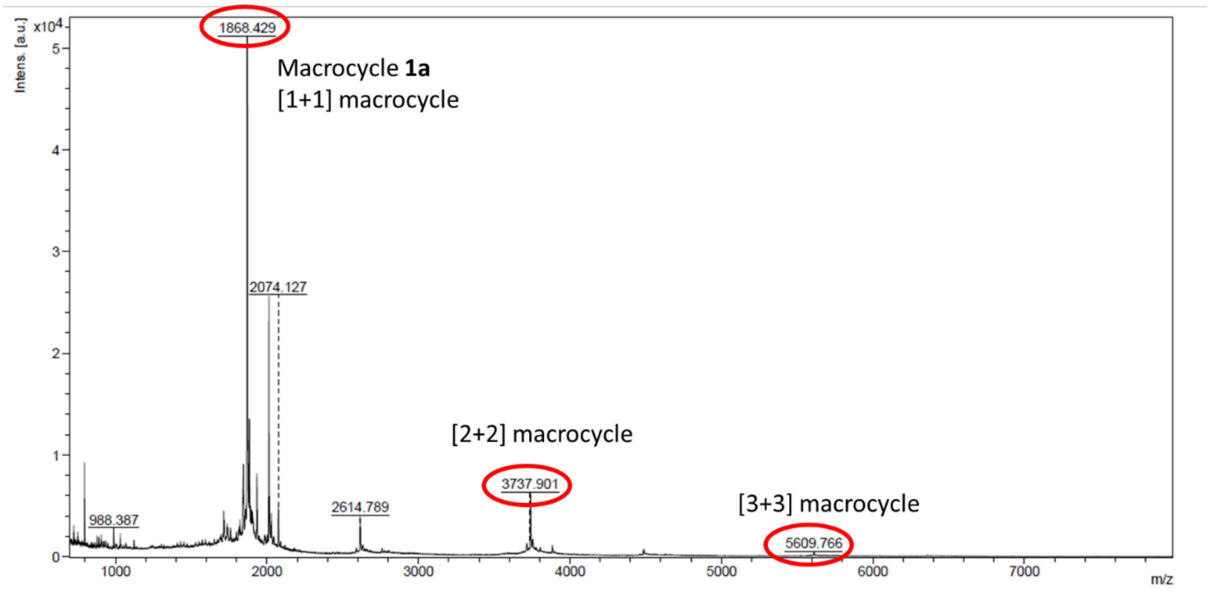

MALDI (TOF) mass spectrum of a crude reaction mixture of **1a**. This shows the formation of larger macrocyclic [2+2] and [3+3] side products, alongside the target [1+1] macrocycle **1a**.

Acyclic bis-triazole PDI 3b

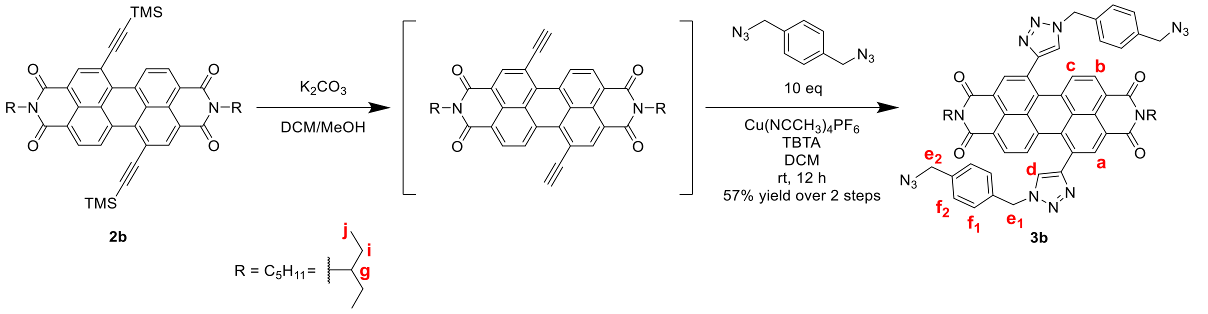

To a solution of TMS-protected bis-alkyne PDI **2b** (85 mg, 118 µmol) in DCM (20 ml) was added K_2CO_3 (50 mg) in MeOH (10 ml). The mixture was stirred at rt for 3 min, and completion of the reaction was confirmed by TLC. A further 20 mL of DCM was added to the solution. The solution was then washed with 1 M HCl (2 x 30 mL), water (2 x 30 mL) and brine (30 ml). The organic layer was then dried over anhydrous $MgSO_4$ and concentrated to dryness *in vacuo* to afford the deprotected PDI bis-alkyne which was used immediately without further purification. This PDI bis-alkyne was immediately re-dissolved in dry DCM (25 mL). To this was added 1,4-bis(azidomethyl)benzene (220 mg, 1.18 mmol, 10 equiv) and Tris((1-benzyl-4-triazolyl)methyl)amine (TBTA) (12 mg, 24 µmol, 0.2 equiv). The solution was then de-gassed with argon. The copper (I) catalyst $Cu(CH_3CN)_4PF_6$ (9 mg, 24 µmol, 0.2 equiv) was then added and the solution was once again de-gassed with argon. The reaction was stirred at rt for 12 h. The solvent was then removed *in vacuo*. The resulting residue was purified by silica gel flash column chromatography (1:99 MeOH-DCM) affording the title compound (as the pure 1,7-regioisomer) as a purple solid (63 mg, 66 µmol, 57%).

^{1}H NMR (500 MHz, Chloroform-*d*) δ 8.64 (s, 2H$_a$), 8.11 (d, *J* = 8.0 Hz, 2H$_b$), 7.82 (d, *J* = 8.1 Hz, 2H$_c$), 7.65 (s, 2H$_d$), 7.41 – 7.34 (m, 8H$_f$), 5.66 (s, 4H$_{e1}$), 5.02 (m, 2H$_g$), 4.41 (s, 4H$_{e1}$), 2.28 – 2.16 (m, 4H$_i$), 1.94 – 1.85 (m, 4H$_i$), 0.88 (t, *J* = 7.5, 1.3 Hz, 12H$_j$).

^{13}C NMR (126 MHz, $CDCl_3$) δ 163.81, 148.51, 136.81, 135.00, 134.58, 134.48, 134.18, 133.49, 129.87, 129.20, 129.18, 129.04, 128.96, 128.59, 128.55, 128.52, 122.81, 121.96, 57.74, 54.34, 54.30, 25.08, 11.36.

HRMS (ESI) (m/z) calculated for $C_{54}H_{47}N_{14}O_4^+$ [M+H$^+$] 955.3904, found 955.3885

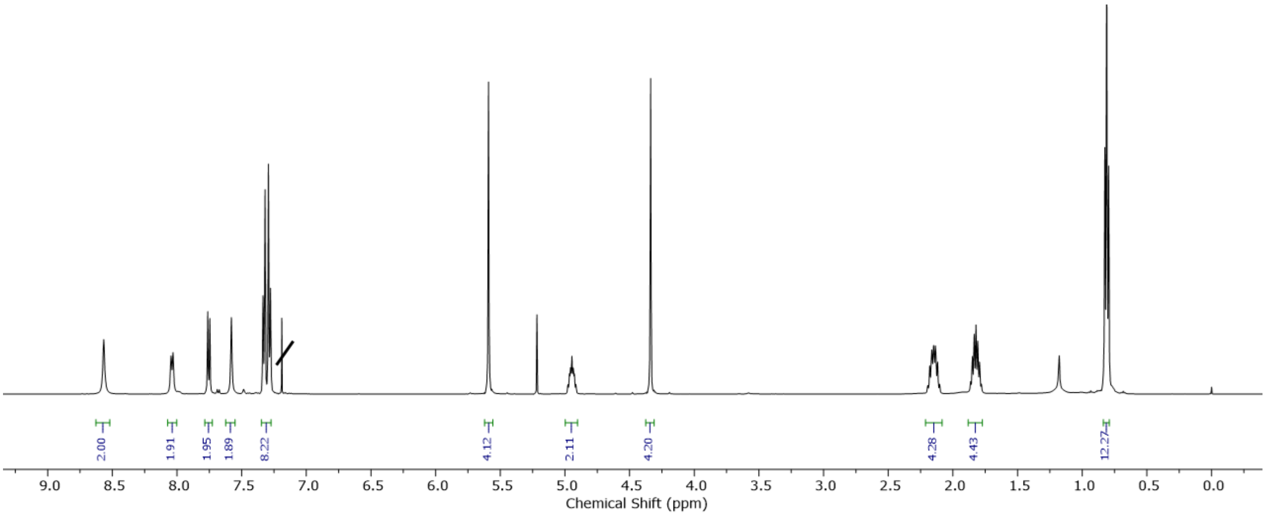

^{1}H NMR spectrum of **3b** (Chloroform-*d*, 298 K, 500 MHz).

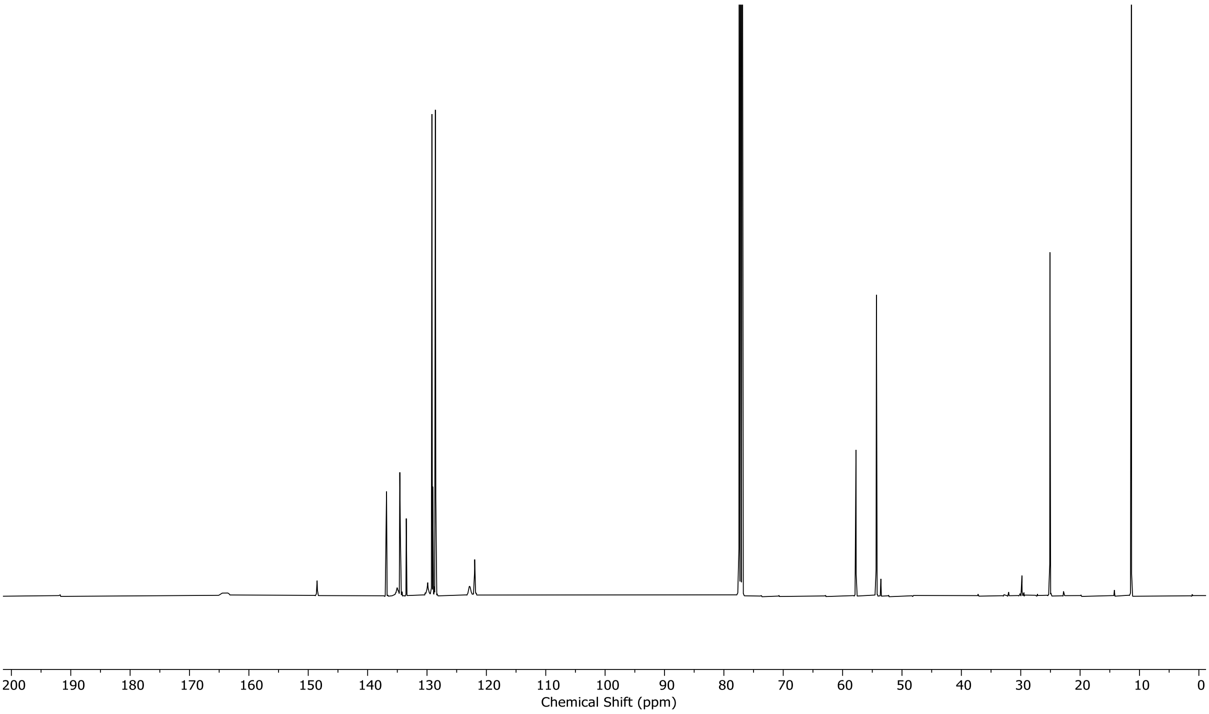

^{13}C NMR spectrum of **3b** (Chloroform-*d*, 298 K, 101 MHz).

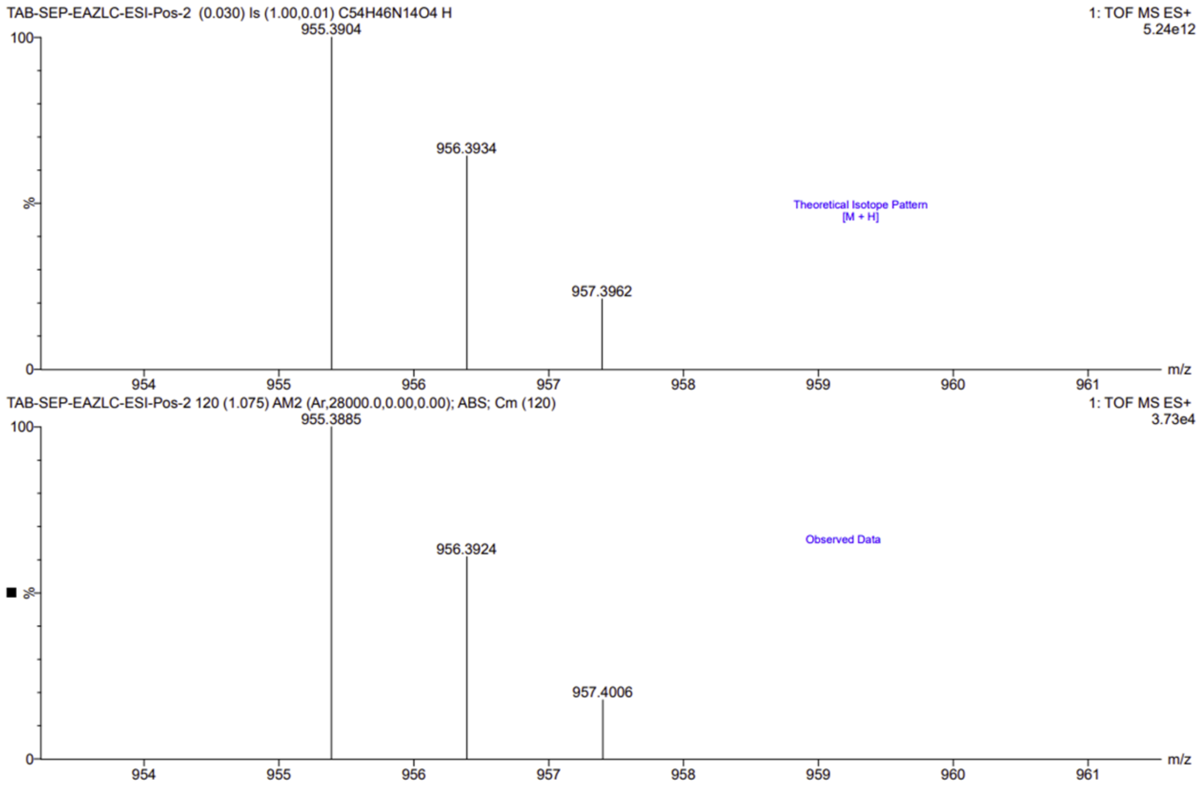

Calculated (top) and observed (bottom) ESI MS data for compound **3b**.

Bis-PDI macrocycle 1b

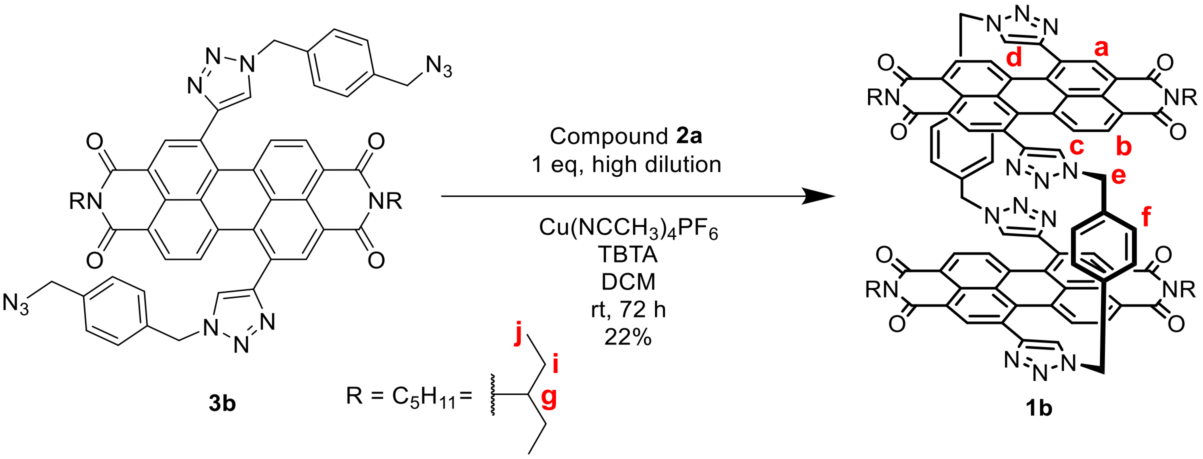

To a solution of compound **3b** (63 mg, 66 µmol) and freshly-prepared compound **2a** (38 mg, 66 µmol, 1 equiv) in DCM (250 mL) was added Tris((1-benzyl-4-triazolyl)methyl)amine (TBTA) (14 mg, 26 µmol, 0.4 equiv). The solution was then de-gassed with argon. The copper catalyst $Cu(CH_3CN)_4PF_6$ (10 mg, 26 µmol, 0.4 equiv) was then added and the solution was once again de-gassed with argon. The reaction was stirred at rt for 36 h and monitored by TLC (1:99 MeOH-DCM). The solvent was then removed *in vacuo*. The resulting residue was purified by silica gel flash column chromatography (1:99 MeOH-DCM) followed by preparative silica TLC (0.5:99.5 MeOH-DCM), affording the title compound (as the pure 1,7-regioisomer) as a purple solid (22.7 mg, 15 µmol, 22%).

^{1}H NMR (400 MHz, TCE-d_2, 373K, the major species are the enantiomers, i.e. ***MM*,*PP:PM*** = 81:19 mol%) δ 8.63 (s, 4H$_a$), 8.23 (d, J = 8.1 Hz, 4H$_b$), 7.79 (d, J = 8.1 Hz, 4H$_c$), 7.31 (s, 8H$_f$), 7.20 (s, 4H$_d$), 5.74 (d, J = 15.2 Hz, 4H$_e$), 5.64 (d, J = 15.2 Hz, 4H$_e$), 5.02 – 4.95 (m, 4H$_g$), 2.18 (m, 8H$_i$), 1.98 (m, 8H$_i$), 0.90 (q, J = 7.2 Hz, 24H$_j$).

^{13}C NMR (101 MHz, TCE, 373K) δ 164.53, 163.49, 148.29, 136.30, 134.87, 134.02, 133.08, 129.91, 129.49, 129.37, 128.62, 128.25, 123.15, 121.25, 58.74, 54.18, 29.78, 25.43, 25.22, 11.53.

HRMS (ESI) (m/z) calculated for $C_{92}H_{76}N_{16}O_8$ [M]$^+$ 1533.6111, found 1533.6090.

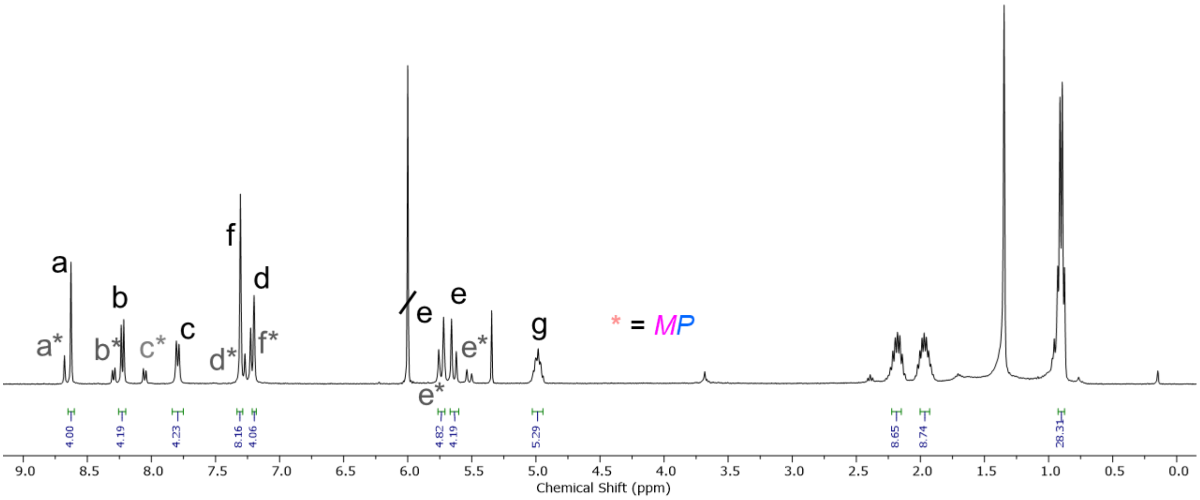

^{1}H NMR spectrum of **1b** (TCE-d_2, 373K, 400 MHz). The δ = 5-9 ppm region of this spectrum is near identical (Δδ = 0.05 - 0.1 ppm) to that of macrocycle **1a**. Peaks labelled with an asterisk correspond to the minor species (***MP*** diastereomer), while those without an asterisk correspond to the major species (***MM,PP*** enantiomers).

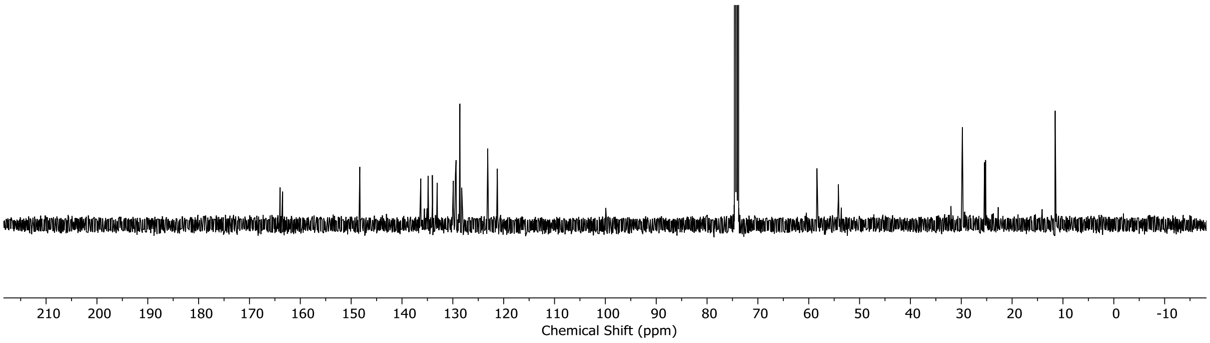

^{13}C NMR spectrum of **1b** (TCE-d_2, 373 K, 101 MHz).

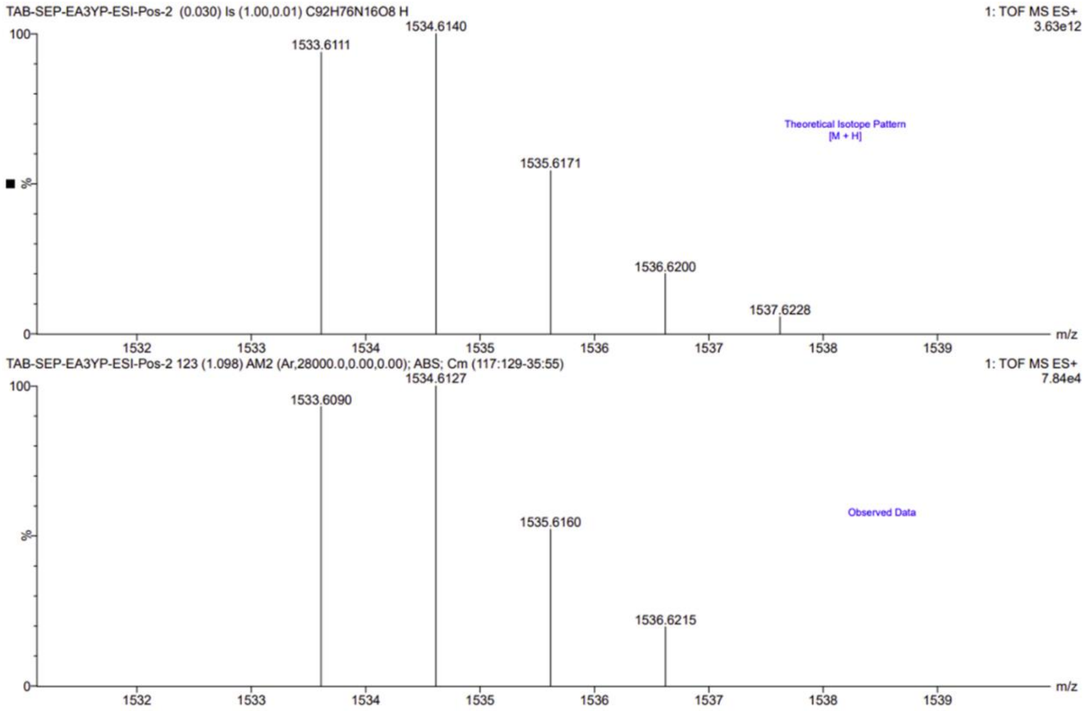

Calculated (top) and observed (bottom) ESI MS data for compound **1b**.

1,7-TMS-protected bis-alkyne PDI 5b.

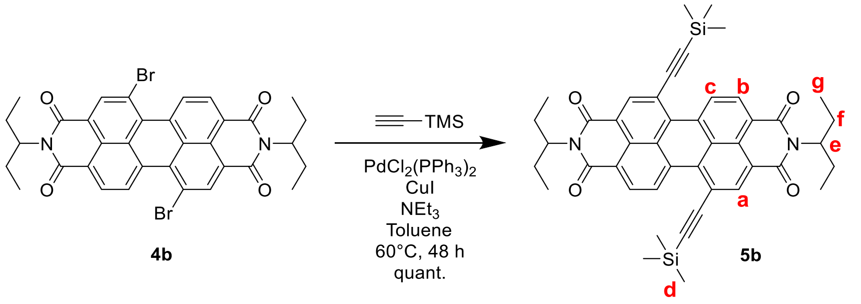

To a solution of dibromo PDI **4b**[3] (200 mg, 290 µmol) in 1:1 dry NEt_3-toluene (30mL) under a nitrogen atmosphere was added $Pd(PPh_3)_2Cl_2$ (12.94 mg, 17 µmol, 0.06 equiv), CuI (6.09 mg, 32 µmol, 0.11 equiv) and trimethylsilylacetylene (142 mg, 207 µL, 1.45 mmol, 5 equiv). The mixture was thoroughly de-gassed with nitrogen and stirred at 60 °C for 48 h. The solvent mixture was then removed *in vacuo*. The resulting residue was then re-dissolved in DCM (30 mL) and washed with 1M HCl (50 mL) and water (3 x 50 mL); dried over anhydrous $MgSO_4$ and concentrated to dryness *in vacuo*. The resulting residue was purified by silica gel flash column chromatography (1:1 n-hexane:DCM) affording the title compound as a red solid (210mg, 290 µmol, 100%).

^{1}H NMR (400 MHz, Chloroform-*d*) δ 10.22 (d, *J* = 8.2 Hz, $2H_c$), 8.82 (s, $2H_a$), 8.65 (d, *J* = 8.2 Hz, $2H_b$), 5.11 – 5.02 (m, $2H_e$), 2.33 – 2.19 (m, $4H_f$), 2.02 – 1.81 (m, $4H_f$), 0.93 (t, *J* = 7.5 Hz, $12H_g$), 0.39 (s, $18H_d$).

^{13}C NMR (101 MHz, $CDCl_3$) δ 134.49, 134.21, 128.13, 127.83, 127.66, 120.02, 106.19, 105.81, 77.48, 77.16, 76.84, 57.94, 25.17, 11.44, -0.23

MS (MALDI-TOF) (m/z) calculated for $C_{44}H_{46}N_2O_4Si_2$ [M]: 722.2996, found 722.3007

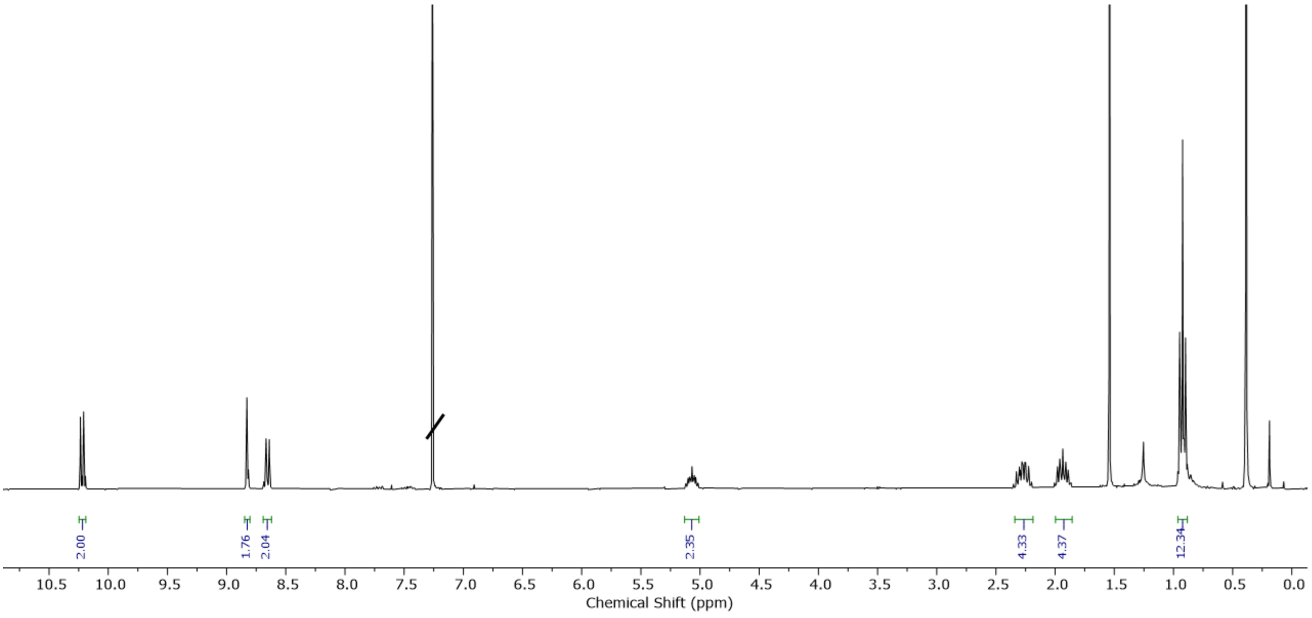

^{1}H NMR spectrum of **5b** (Chloroform-*d*, 298 K, 400 MHz).

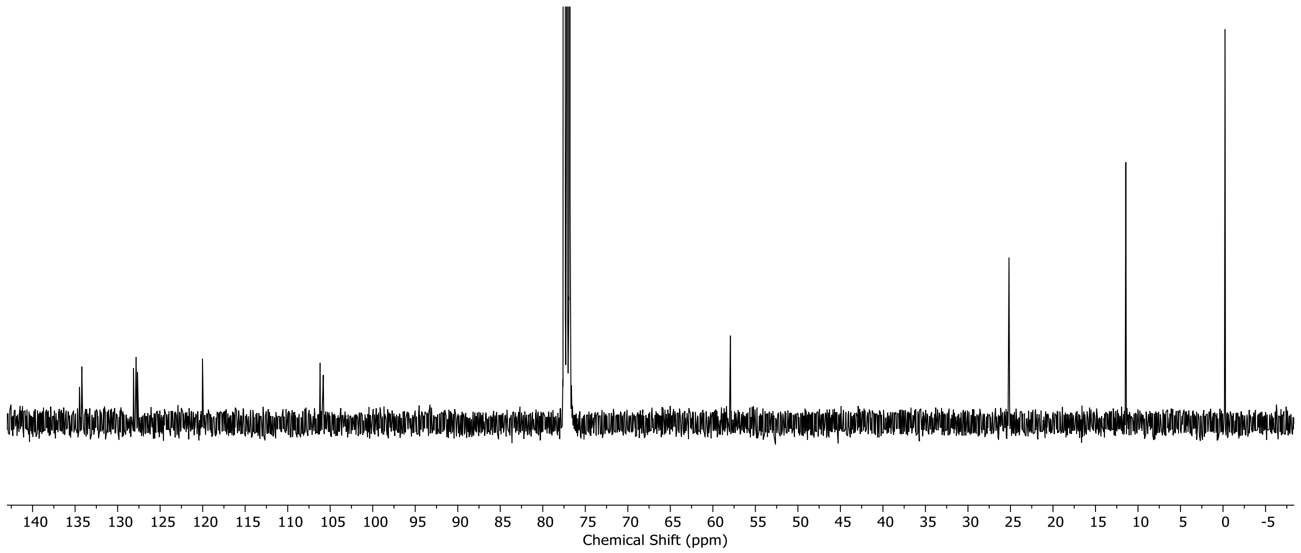

^{13}C NMR spectrum of **5b** (Chloroform-*d*, 298 K, 400 MHz).

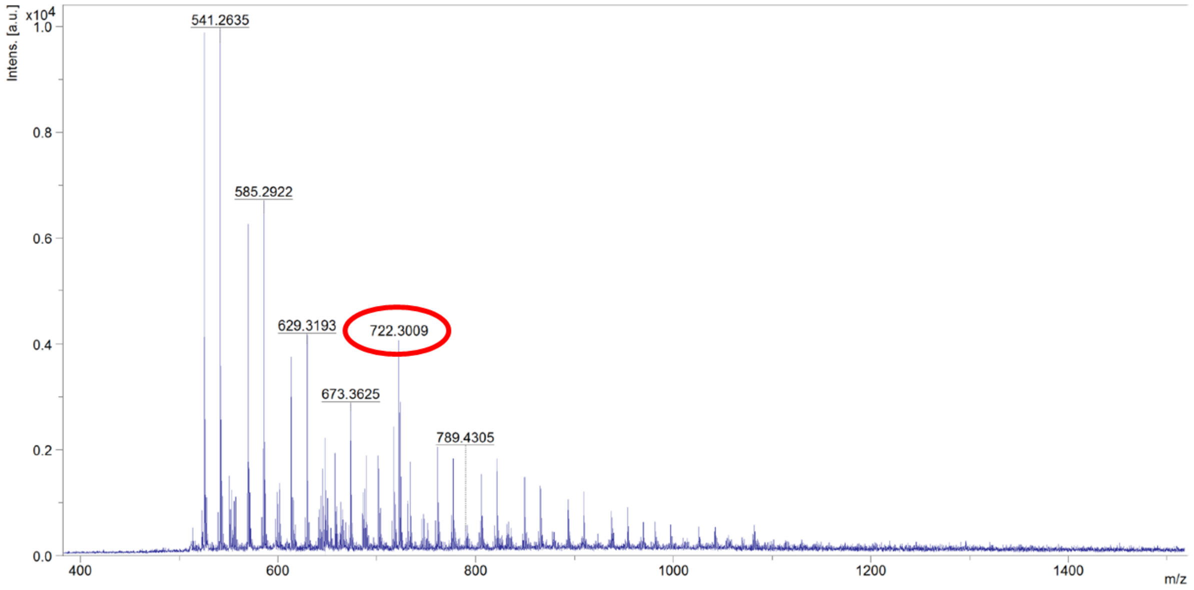

MALDI (TOF) mass spectrum for compound **5b**. An accurate mass was obtained by calibration to polyethylene glycol chains that were co-spotted with the sample and therefore also observed in the mass spectrum

2. Further NMR spectroscopy experiments

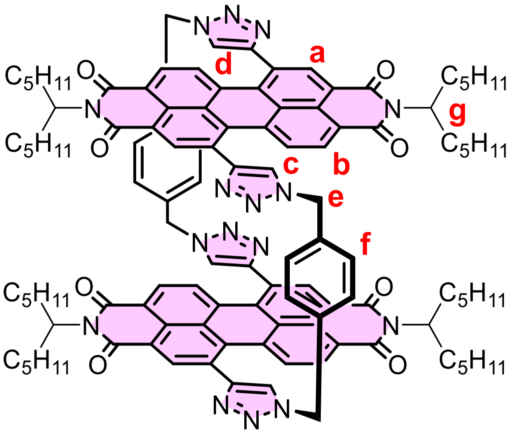

Macrocycle **1a**

a) Variable temperature ^{1}H NMR spectroscopy experiments

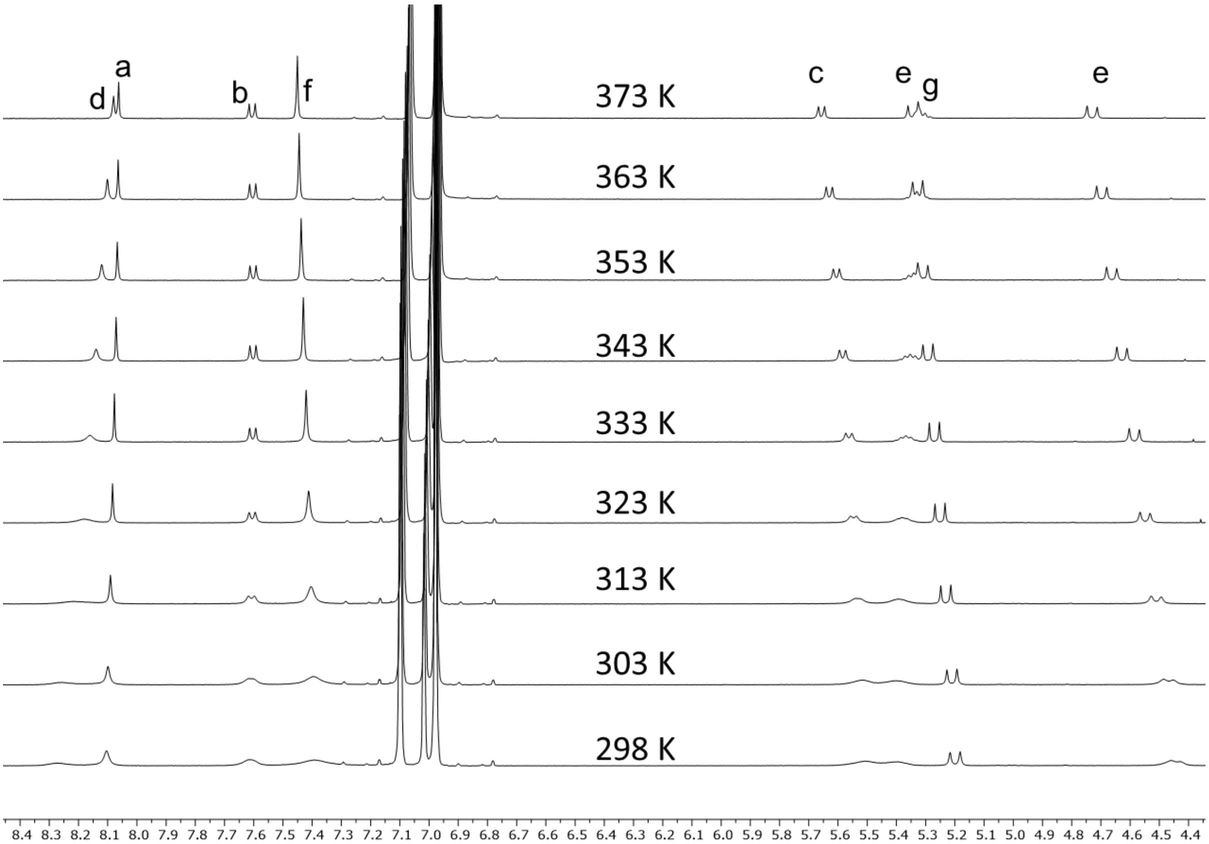

Figure S1: Truncated ^{1}H NMR spectra of **1a** (Toluene-d_8, 400 MHz) at various temperatures ranging from 298 K (bottom spectrum) to 373 K (top spectrum), showing how the spectrum is too broad to assign at room temperature (298K) but sharpens as the temperature is increased.

b) Solvent dependent ^{1}H NMR spectroscopy of macrocycle 1a

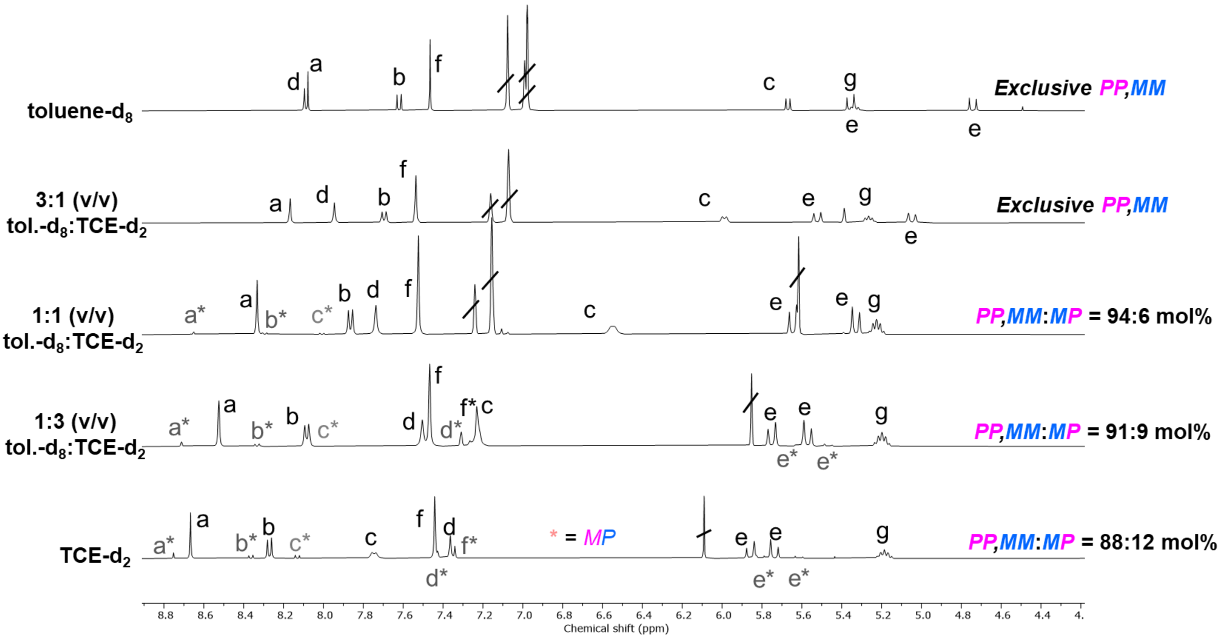

Figure S2: Partial ^{1}H NMR spectra of macrocycle **1a** in different toluene-d$_8$:TCE-d$_2$ solvent mixtures (373K, 400 MHz). The spectra are aligned using an internal reference standard, poly(dimethylsiloxane), added to each sample. Peaks labelled with an asterisk correspond to the minor species (***MP*** diastereomer), while those without an asterisk correspond to the major species (***MM***,***PP*** enantiomers). The ***MM,PP:MP*** ratios are shown for each spectrum.

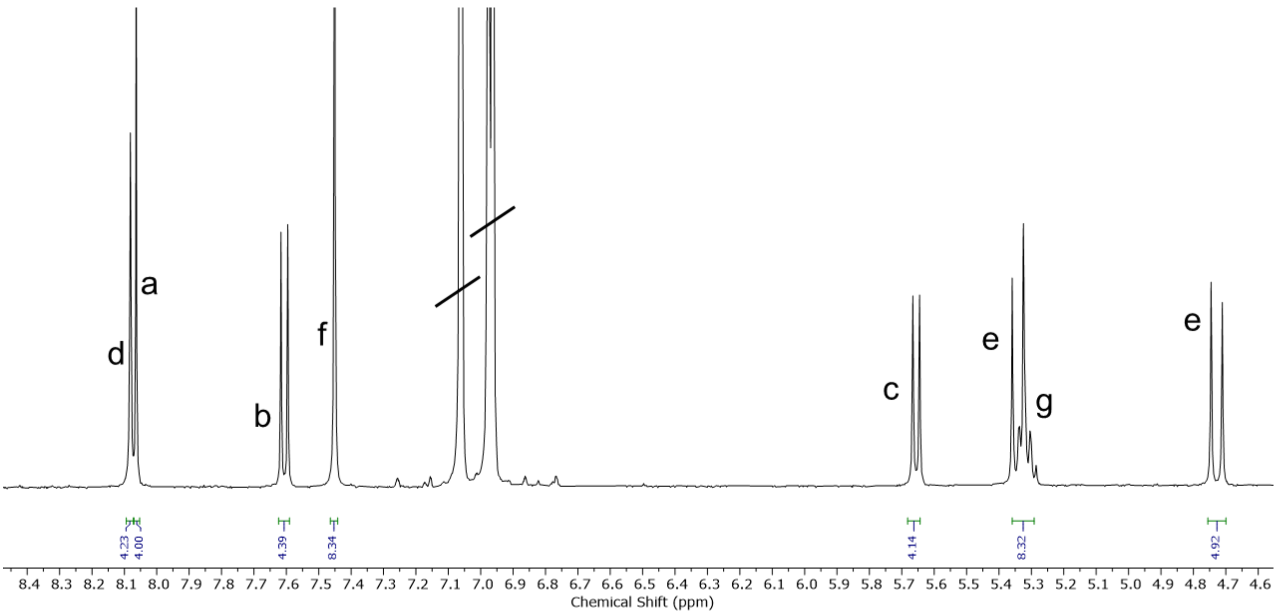

Figure S3: Zoomed in region of the ^{1}H NMR spectrum of macrocycle **1a** in toluene-*d$_8$* (373 K, 400 MHz, ***MM,PP:PM*** > 99:1 mol%) to show that no ***MP*** diastereomer (i.e. minor species) is detectable by ^{1}H NMR spectroscopy.

c) ^{1}H-^{1}H EXSY/NOE NMR Spectroscopy

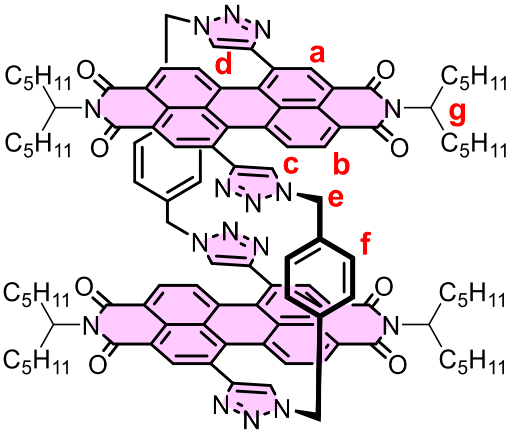

Macrocycle **1a**

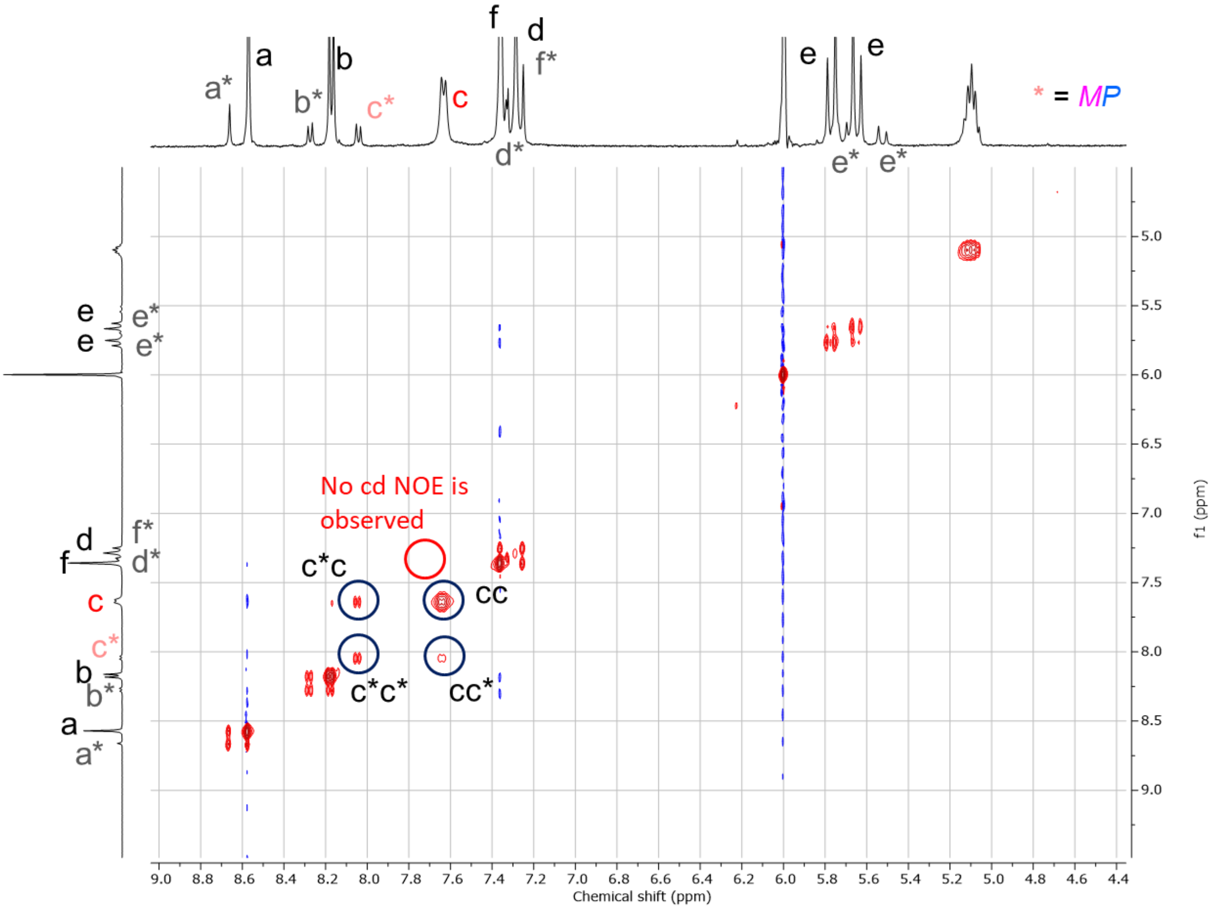

Figure S4: Partial ^{1}H-^{1}H EXSY/NOESY NMR spectrum of macrocycle **1a** (TCE-d_2, 373 K, 400 MHz). Signals with phase shown in red are EXSY signals and signals with phase shown in blue are NOESY signals.

Unlike in toluene (**Figure S5**), no NOE is seen between the H_c and H_d protons in TCE-d_2. EXSY signals show which ^{1}H NMR signals are under chemical exchange, via interconversion of ***MM,PP*** enantiomers with the ***MP*** diastereomer. The EXSY signal set for the exchange of H_c (***MM,PP*** enantiomer species) and H_{c*} (***MP*** diastereomer) is highlighted as an example.

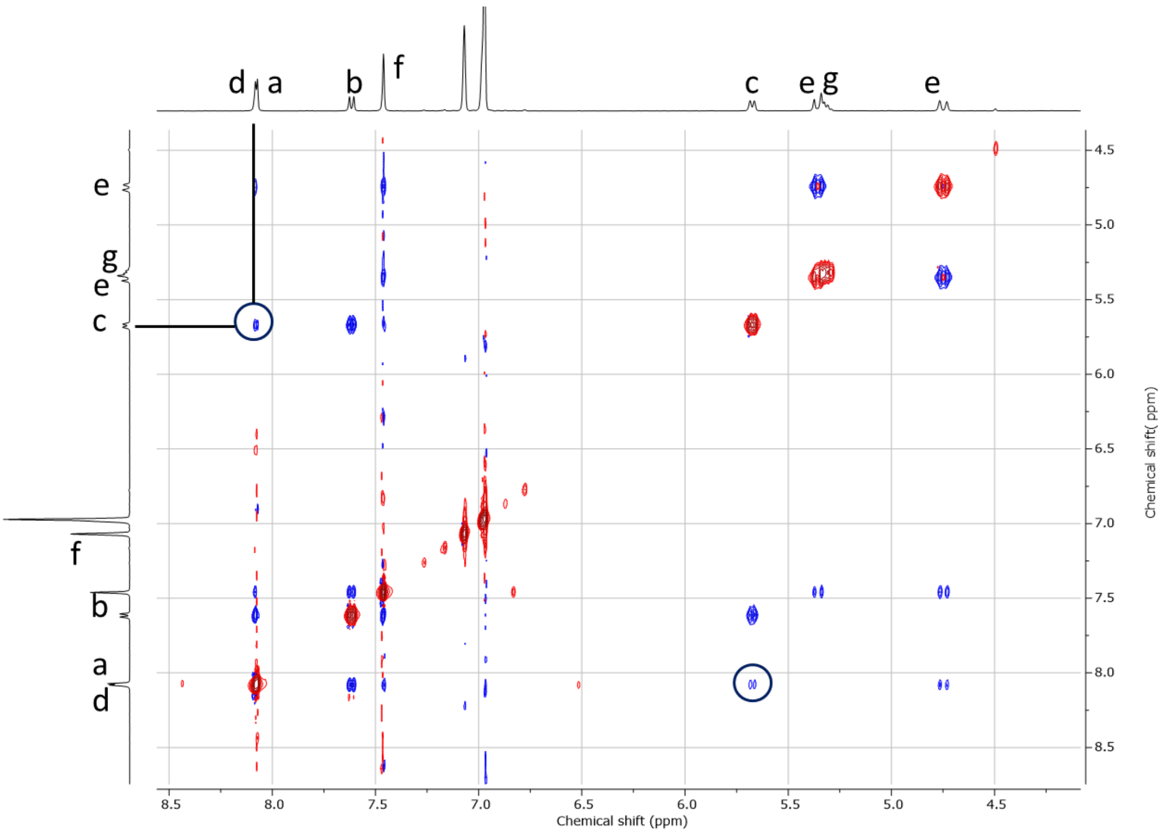

Figure S5 Partial ^{1}H-^{1}H EXSY/NOESY NMR spectrum of macrocycle **1a** (toluene-d_8, 373 K, 400 MHz). Signals with phase shown in red are EXSY signals and signals with phase shown in blue are NOESY signals.

In toluene-d_8 a NOE between the protons H_c and H_d is observed, in contrast to TCE-d_2 (**Figure S4**).

d) Determination of rate constants of *MM/PP*↔*MP* interconversion by ^{1}H-^{1}H EXSY NMR spectroscopy

Quantitative ^{1}H-^{1}H EXSY NMR spectroscopy can be used to obtain rate constants for the interconversion between different species.[4] This has been used to quantify the exchange kinetics in supramolecular systems where the exchanging species have different energies and populations.[5] An adapted version of this method has been used here.

All 2D-EXSY NMR spectra were recorded on a Bruker AV NEO 400 (400 MHz) NMR spectrometer. Exchange rates were calculated using the program EXSY CALC.[6] To calculate the exchange rates between two species, diagonal and cross-peak intensities for the exchanging NMR resonances are required from two EXSY NMR experiments at different mixing times. For each data point, one EXSY NMR experiment was carried out with a mixing time of 900 ms and another was carried out with a very short mixing time of 5 ms. The same major and minor exchanging proton signals were used for every EXSY experiment. Here, we used H_c and H_{c*} in the ^{1}H NMR spectrum of **1a** (**Figure 4**), as the large chemical shift difference between these signals allowed easy and reliable integration (Δδ = 0.5 ppm).

The exchange matrix is as follows:

$$\begin{vmatrix} -R_1 - k_1 & k_{-1} \\ k1 & -R_2 - k_{-1} \end{vmatrix}$$

Where k_1 and k_{-1} are exchange rates and R_1 and R_2 are the longitudinal relaxation rates.

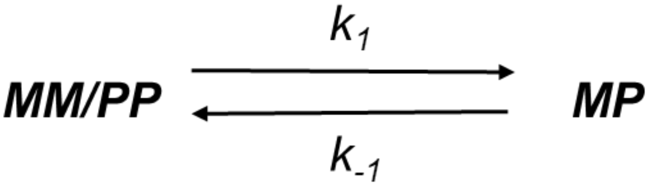

Table S1: Rates (k_1 and k_{-1}) and corresponding free energies of activation ($\Delta G^{\ddagger}_1$ and $\Delta G^{\ddagger}_{-1}$) for the interconversion between enantiomer ***MM/PP*** and diastereomer ***MP*** of macrocycle **1a**, determined by ^{1}H-^{1}H EXSY NMR spectroscopy at different temperatures and in different solvents.

Solvent	Temp. (K)	k_1 (s^{-1})	$\Delta G^{\ddagger}_1$ (kJ mol^{-1})	k_{-1} (s^{-1})	$\Delta G^{\ddagger}_{-1}$ (kJ mol^{-1})
TCE-d_2	353	0.023	103.8	0.086	99.7
	358	0.031	102.9	0.159	97.8
	363	0.043	101.8	0.206	97.0
	368	0.071	100.2	0.372	95.1
	373	0.103	99.1	0.574	93.8
1:1 (v/v) tol.-d_8:TCE-d_2	373	0.090	99.5	1.05	91.9

This data in pure TCE-d_2 can be fitted to the linear form of the Van 't Hoff equation: [7]

$$\ln\frac{k}{T} = \frac{-\Delta H^{\neq}}{R}\frac{1}{T} + \ln\frac{k_B}{h} + \frac{\Delta S^{\neq}}{R}$$

Where k_B is the Boltzmann constant, R is the gas constant and h is Planck's constant.

This fitting yields $\Delta H^{\ddagger}$ = 80.7 ± 5.7 kJ mol^{-1} and $\Delta S^{\ddagger}$ = – 49.7 ± 14.1 J K^{-1} mol^{-1} for the forwards ***MM/PP*** → ***MP*** process and $\Delta H^{\ddagger}$ = 98.7 ± 6.2 kJ mol^{-1} and $\Delta S^{\ddagger}$ = + 13.0 ± 17.0 J K^{-1} mol^{-1} for the backwards ***MP*** → ***MM/PP*** process, in pure TCE-d_2.

From here, ΔG‡ in pure TCE-d$_2$ can be calculated at 298 K: $\Delta G^{\ddagger}_1$ (298 K) = 95.5 kJ mol^{-1} for the forwards ***MM/PP*** → ***MP*** process and $\Delta G^{\ddagger}_{-1}$ (298 K) = 94.8 kJ mol^{-1} for the backwards ***MP*** → ***MM/PP*** process. Therefore, the barrier in TCE-d$_2$ at 298 K is close to that in dichloromethane at 298 K, as determined by CD spectroscopy in SI **Section 5b**.

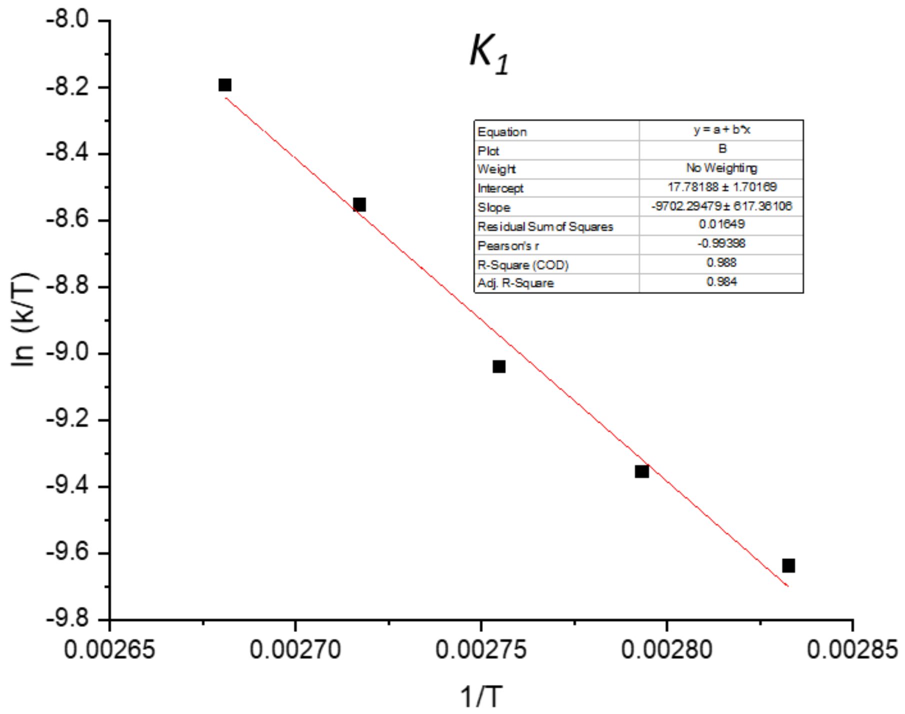

Figure S6: Van 't Hoff plot for the forwards rate constant (k_1) of interconversion between the enantiomer ***MM/PP*** and the diastereomer ***MP*** of macrocycle **1a**, obtained from ^{1}H-^{1}H EXSY NMR experiments at different temperatures.

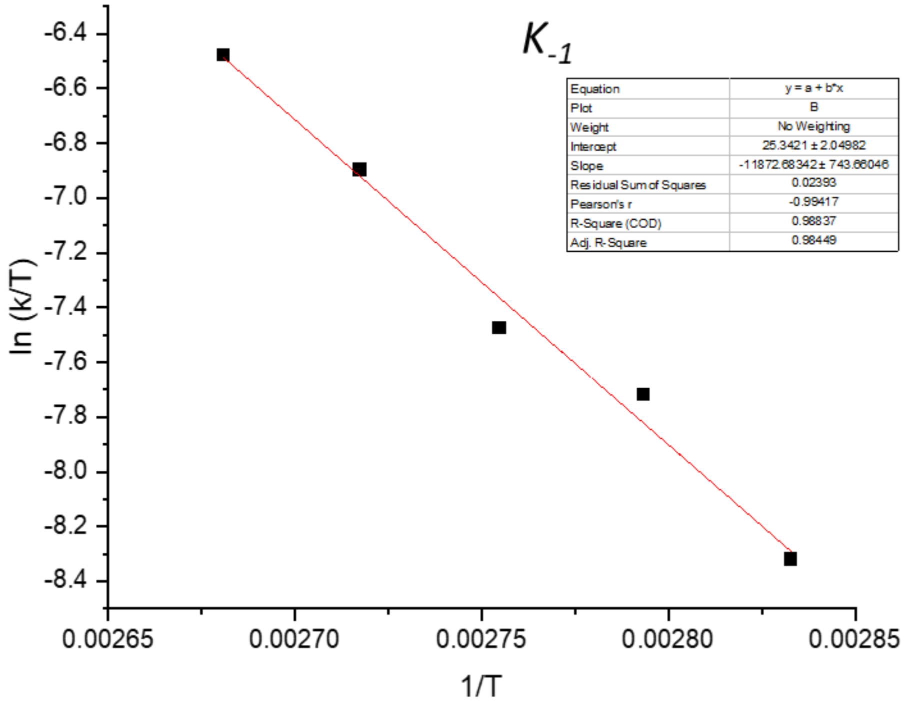

Figure S7: Van 't Hoff plot for the backwards rate constant (k_{-1}) of interconversion between the enantiomer ***MM/PP*** and the diastereomer ***MP*** of macrocycle **1a**, obtained from ^{1}H-^{1}H EXSY NMR experiments at different temperatures.

3. X-ray crystallography

Purple, needle-like crystals of macrocycle **1b**, suitable for single crystal X-ray diffraction, were grown from a racemic mixture of **1b** dissolved in chloroform, with slow diffusion of methanol (antisolvent).

Single crystal X-ray diffraction experiments were performed at the UK Diamond Light Source I19-1 3-circle diffractometer (λ = 0.6889 Å).[8-10] A suitable single crystal was selected and mounted using fomblin film on a micromount. Data were collected on a dectris-CrysAlisPro-abstract goniometer imported dectris images diffractometer. The crystals were kept at 100(2) K during data collection (single omega sweep). The structures were solved by direct methods using ShelXT[11] and refined with ShelXL using a least squares method. Olex2 software was used as the solution, refinement and analysis program.[12] The crystal diffracted weakly despite the use of synchrotron radiation and numerous attempts at growing better diffracting crystals. The data used in the refinement was truncated to a resolution of 0.84 Å which reduced completeness but improved signal to noise. Overall the data to parameter ratio is 11.5.A completeness of 83% did not support meaningful modelling of most of the disordered solvent. Instead, the olex solvent mask function was used which found 672 electrons in a volume of 2296 $Å^3$ in 2 voids per unit cell. This is consistent with the presence of 0.625[$CHCl_3$], 2.625[COH_4] per asymmetric unit which account for 668 electrons per unit cell. Hydrogen atoms were placed in geometrically calculated positions; non-hydrogen atoms were refined with anisotropic displacement parameters. Methyl hydrogens were refined as idealized CH_3 groups with tetrahedral angles. ADPs of atoms on the PDI core were restrained to be similar (SIMU). Figures were produced using CrystalMakerX.

Crystal Data for Macrocycle **1b** $C_{93}H_{80}N_{16}O_9$(M = 1565.73 g/mol): monoclinic, space group C2/c, a = 42.6612(4) Å, b =12.02180(10) Å, c = 34.4752(3) Å, α = 90°, β = 109.6030(10)°, γ = 90°, V = 16656.3(3) $Å^3$, Z = 8, T = 100(2) K, μ(Synchrotron) = 0.078 mm^{-1}, D_{calc} = 1.245, 35021 reflections collected (3.428° ≤ 2Θ ≤ 48.406°), 12270 unique [R_{int} = 0.0521, R_{sigma} = 0.0852] which were all used in the calculations. The final R_1 was 0.1629 ($I > 2\sigma(I)$) and wR_2 was 0.4597 (all data). Deposited cif number: 2157213.

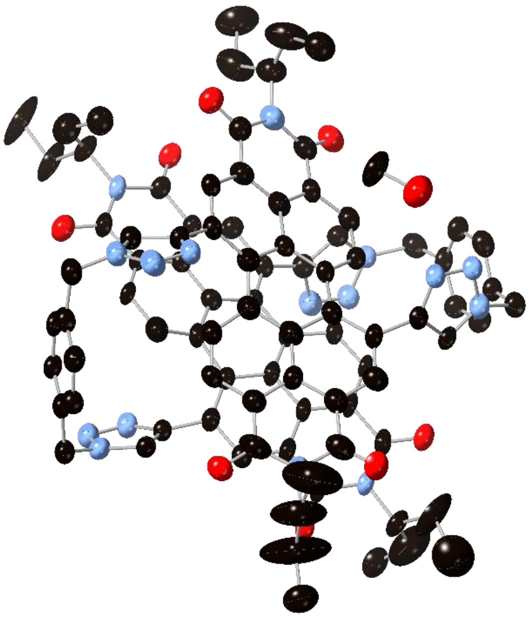

Figure S8: Asymmetric unit of macrocycle **1b** with all non-hydrogen atoms represented by ellipsoids at the 50% probability level. Hydrogen atoms omitted for clarity (C, black; O, red; N, blue)

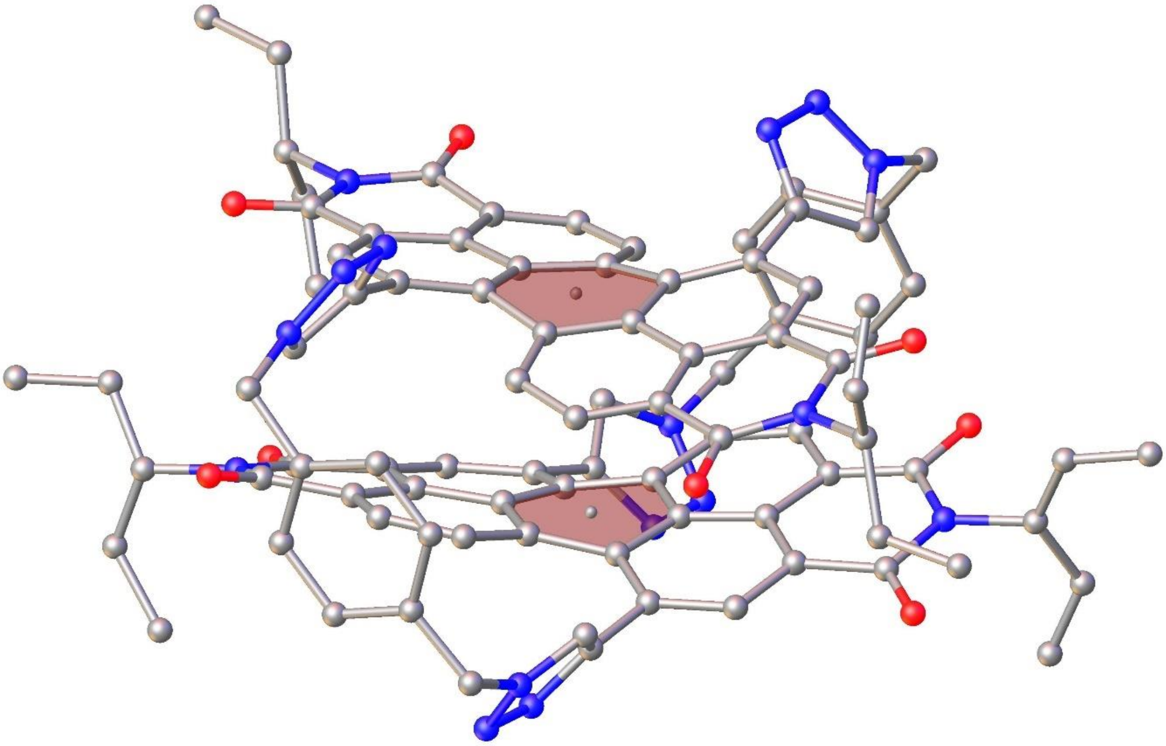

Figure S9: PDI–PDI plane to plane centroid = 3.7 Å, calculated using Olex2 software.

4. Chiral HPLC

a) Methods

Chiral chromatographic studies were performed using a Phenomenex i-Amylose-1 chiral column on an Agilent 1290 Infinity analytical HPLC instrument. The flow rate was 1 mL/minute and the detection wavelength was 500 nm. The eluents and injection volumes for each chromatogram are specified in the figure captions.

To separate the enantiomers for chiroptical studies, the system was set up to run automatedly and the enantiomers were collected using an automated fraction collector. For this purification the eluent system was 4:1 toluene:n-hexane and the injection volume was 20 µL.

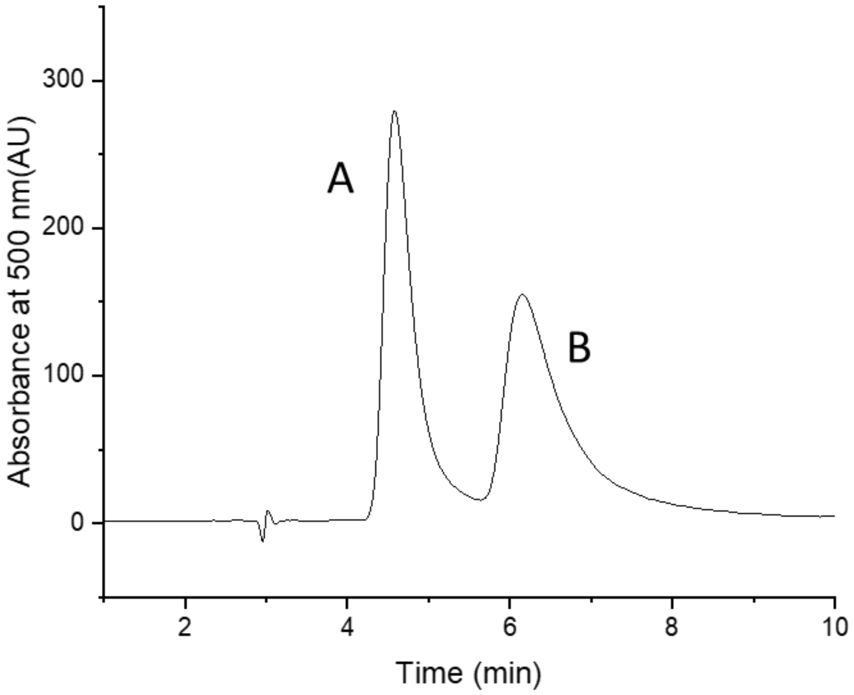

Figure S10: Chiral HPLC chromatogram of compound **1a** dissolved in toluene and eluted with 4:1 (v/v) toluene:n-hexane. Using a combination of CD spectroscopy (**Section 5**) and computational modelling (**Section 9**), peak A is assigned as the ***MM*** enantiomer and peak B is assigned as the ***PP*** enantiomer.

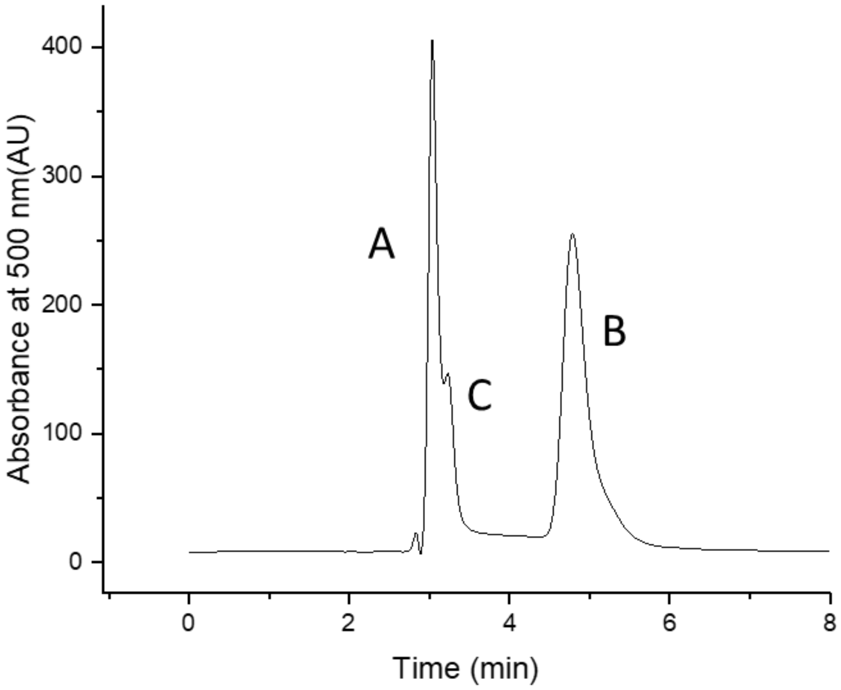

Figure S11: Chiral HPLC chromatogram of compound **1a** dissolved in DCM and eluted with 7:3 (v/v) DCM:n-hexane eluent. From ^{1}H NMR spectroscopy, peaks A and B are assigned to the enantiomers (***PP***,***MM***) and peak C is the diastereomer (***MP***).

b) Racemisation kinetics from chiral HPLC

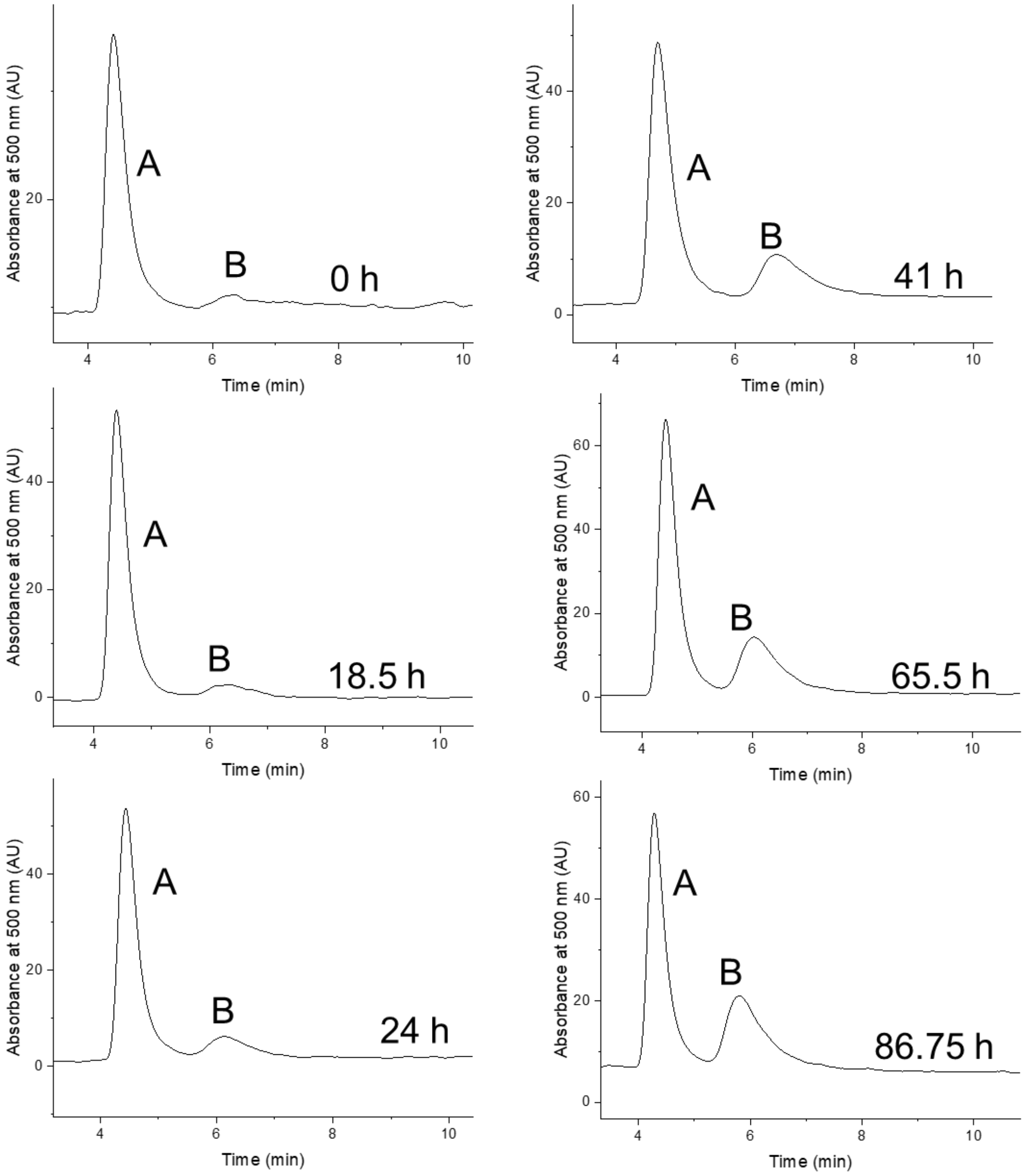

Figure S12: Change in the chiral HPLC chromatogram over time of an enantiopure sample of **1a** ***MM*** **(*MM:PP*** > 99:1 mol%) kept in toluene at room temperature. The chromatograms were run in 80:20 (v/v) toluene:n-hexane eluent.

To measure the rate of racemisation of compound **1a** in toluene, a pure fraction of peak A (***MM*** enantiomer) was obtained by running compound **1a** through the chiral HPLC column in 80:20 toluene: n-hexane eluent. The pure fraction of peak A was dried and re-dissolved in toluene. The sample was kept in toluene, and aliquots of it were re-injected into the column over time, allowing the growth of peak B to be monitored (**Figure S12**). By measuring the ratio of the integrals of peaks A and B an enantiomeric excess ee_t can be calculated for a given time t. The resulting data can then be fitted to the equation:

$$\ln\left({}^{ee_0}/_{ee_t}\right) = 2kt \qquad (\mathbf{S2})$$

Where ee_0 is the enantiomeric excess at $t = 0$ and k is the enantiomerisation rate constant. The racemisation rate constant $k_{rac} = 2k$.[13]

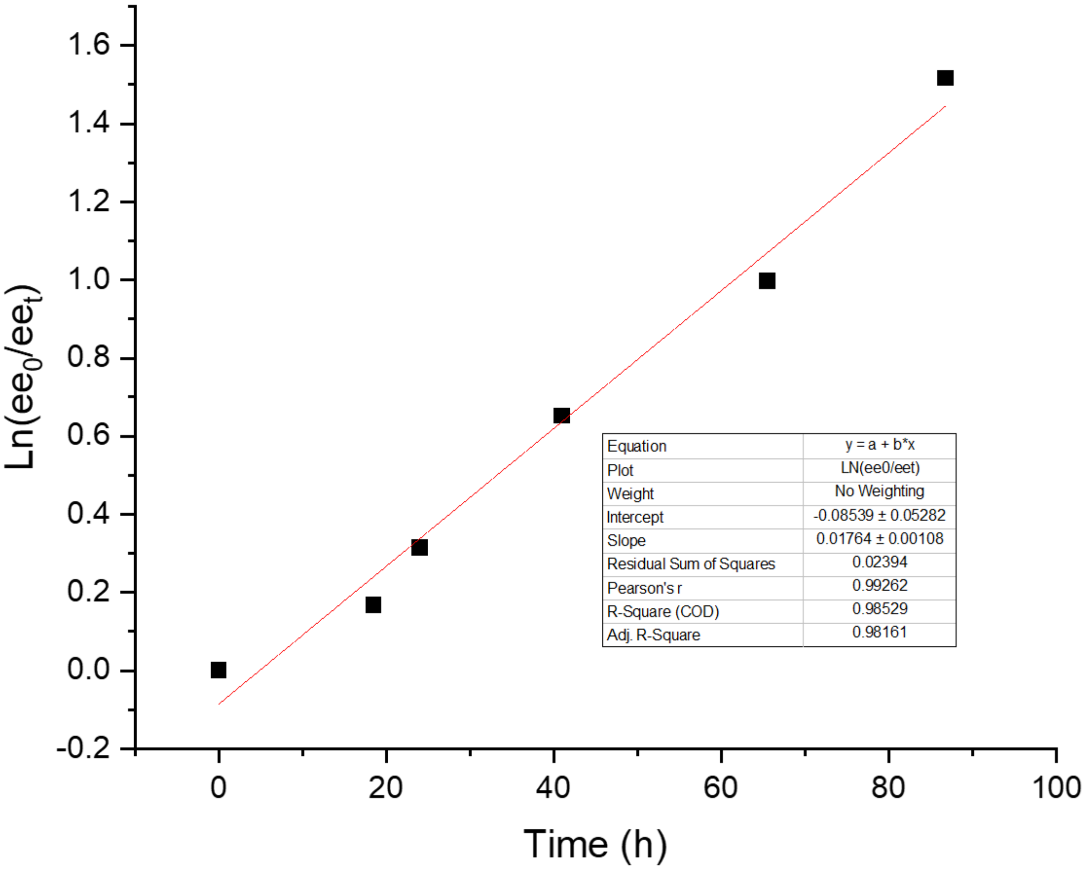

Figure S13: Plot and linear fit of $\ln\left({}^{ee_0}/_{ee_t}\right)$ against t.

The fitting of this data is shown in **Figure S13** and results in a rate constant of enantiomerisation k = 8.82 × 10^{-3} h^{-1} = 2.45 × 10^{-6} s^{-1} and hence a racemisation rate constant k_{rac} = 4.90 × 10^{-6} s^{-1} for toluene at room temperature. A free energy of activation for the racemisation process $\Delta G^‡$ = 104.98 kJ mol^{-1} was determined for toluene at room temperature according to the Eyring equation. This is close agreement with the value for $\Delta G^‡$ determined by time-course CD spectroscopy (**Section 5**).

Additionally, the chromatogram of a sample of peak A (***PP*** enantiomer) that was kept as a dry solid for three months shows very little interconversion has occurred (***MM:PP*** = 95:5 mol%), allowing us to estimate the enantiomer half-life to be $t_{1/2}$ = years in the solid state (**Figure S14**).

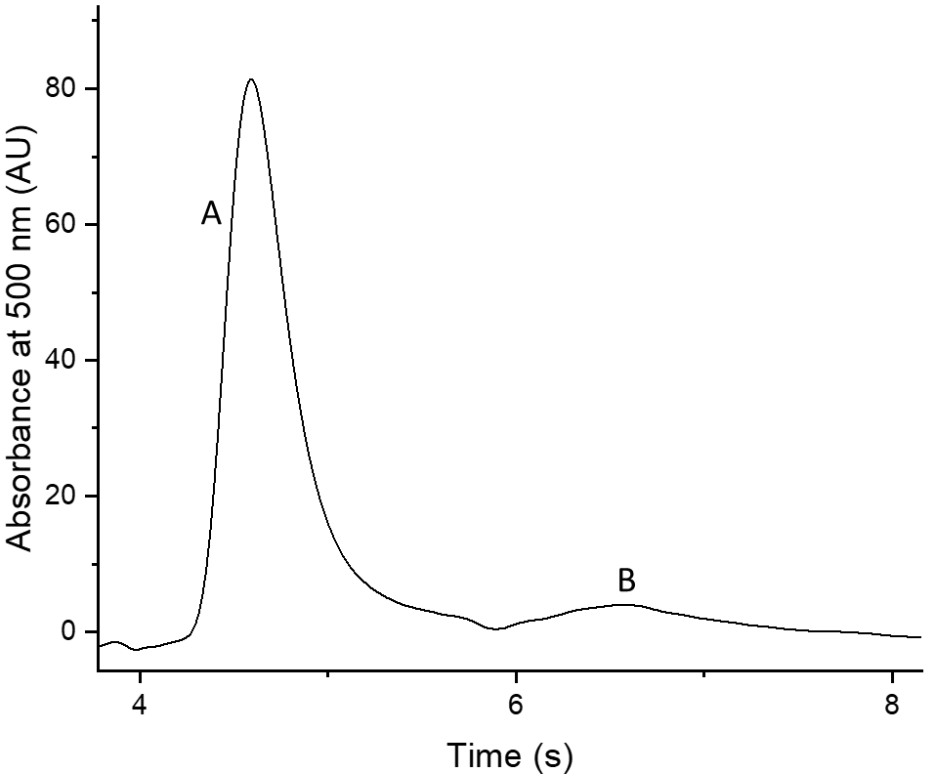

Figure S14: Chiral HPLC chromatogram run in 4:1 (v/v) toluene:n-hexane of a sample of peak A (***MM*** enantiomer) of macrocycle **1a** kept as a dry solid for approximately 3 months (***MM:PP*** = 95:5 mol% by peak integration).

5. Chiroptical studies

a) Circular dichroism studies

Circular dichroism (CD) spectra were recorded on a Jasco J-1500 CD spectrophotometer with a wavelength accuracy ± 0.2 nm (250 to 500 nm), ± 0.5 nm (500 to 800 nm) and a CD root mean square noise < 0.007 mdeg (500 nm). A quartz cuvette with 0.5 mm path length was used. The spectra were recorded at a concentration of 10 µM. The enantiomers were assigned by comparison of their CD spectra in toluene (**Figure S15**) with the computationally calculated spectra of the enantiomers in toluene (**Table S13**).

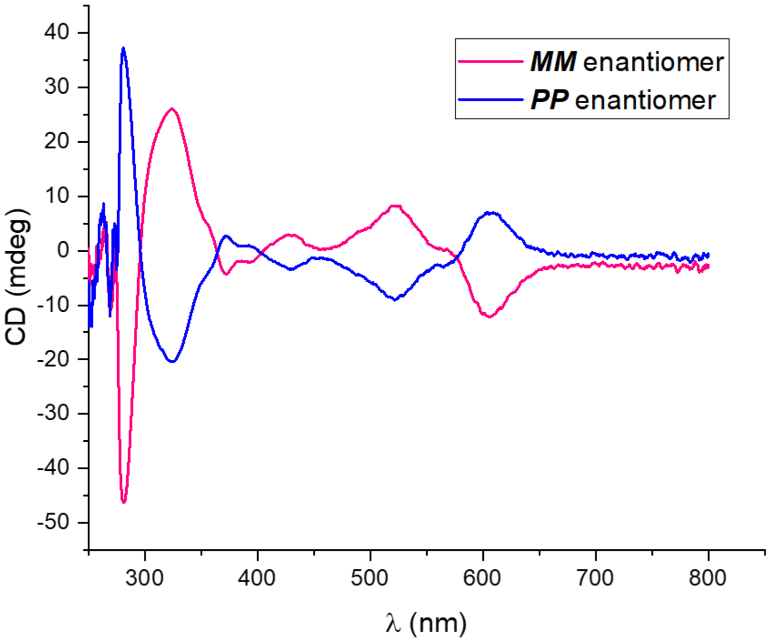

Figure S15: CD spectra for the ***MM*** (***MM*:*PP*** > 99:1 mol%) and ***PP*** (***MM*:*PP*** = 12:88 mol%) enantiomers of macrocycle **1a** in toluene.

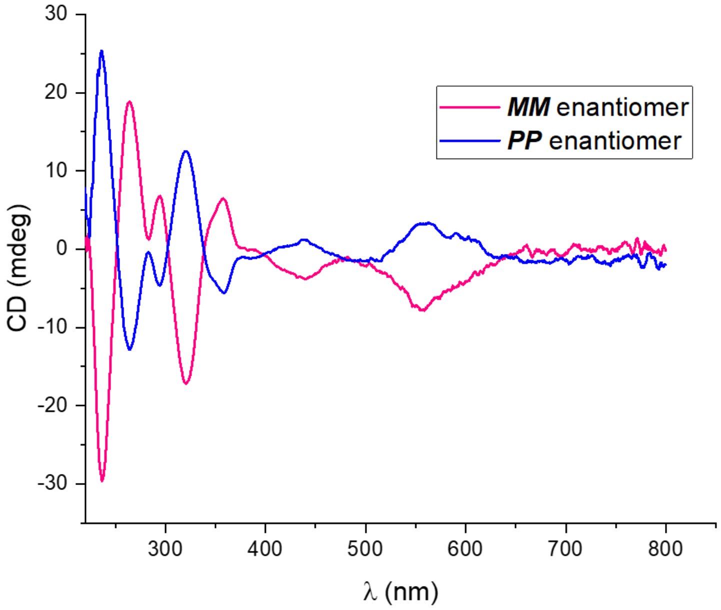

Figure S16: CD spectra for the ***MM*** (***MM*:*PP*** > 99:1 mol%) and ***PP*** (***MM*:*PP*** = 12:88 mol%) enantiomers of macrocycle **1a** in DCM. As the enantiomers racemise relatively quickly in chlorinated solvents ($t_{1/2}$ = 18 min), particular care was taken to record the spectra immediately after dissolving the samples in DCM.

b) Kinetics from time-course CD

To determine the racemisation rate constants, an enantiopure sample of **1a** ***MM*** (10 μM, ***MM*****:*****PP*** > 99:1 mol%) was dissolved in the solvent being tested (toluene or DCM) and kept in a sealed cuvette at 25 °C. The CD spectrum was recorded at regular time intervals. The decay in intensity of the strongest peaks between λ = 250 - 400 nm was monitored over time and the resulting data was fitted to the following equation:

$$\ln\left({CD_0}/{CD_t}\right) = 2kt$$

Where CD_0 is the CD signal intensity at $t = 0$ for a given peak, $\mathrm{CD_t}$ is the CD signal intensity at time t for a given peak and k is the enantiomerisation rate constant. The racemisation rate constant k_{rac} is determined from $k_{rac} = 2k$.[13]

Table S2: Kinetic parameters for the racemisation of an initially pure sample of the ***MM*** enantiomer of macrocycle **1a** in toluene and DCM (298 K) as determined by time-course CD spectroscopy.

Solvent	*k* (s^{-1})	k_{rac} (s^{-1})	$t_{1/2}$ (hours)	ΔG‡ (kJ mol^{-1}, 298 K)
Toluene	7.95×10^{-7} $\pm 7.36 \times 10^{-8}$	1.59×10^{-6} $\pm 1.47 \times 10^{-7}$	121 ± 10	107.8 ± 0.2
DCM	3.20×10^{-4} $\pm 1.36 \times 10^{-5}$	6.40×10^{-4} $\pm 2.27 \times 10^{-5}$	0.3 ± 0.01	92.9 ± 0.1

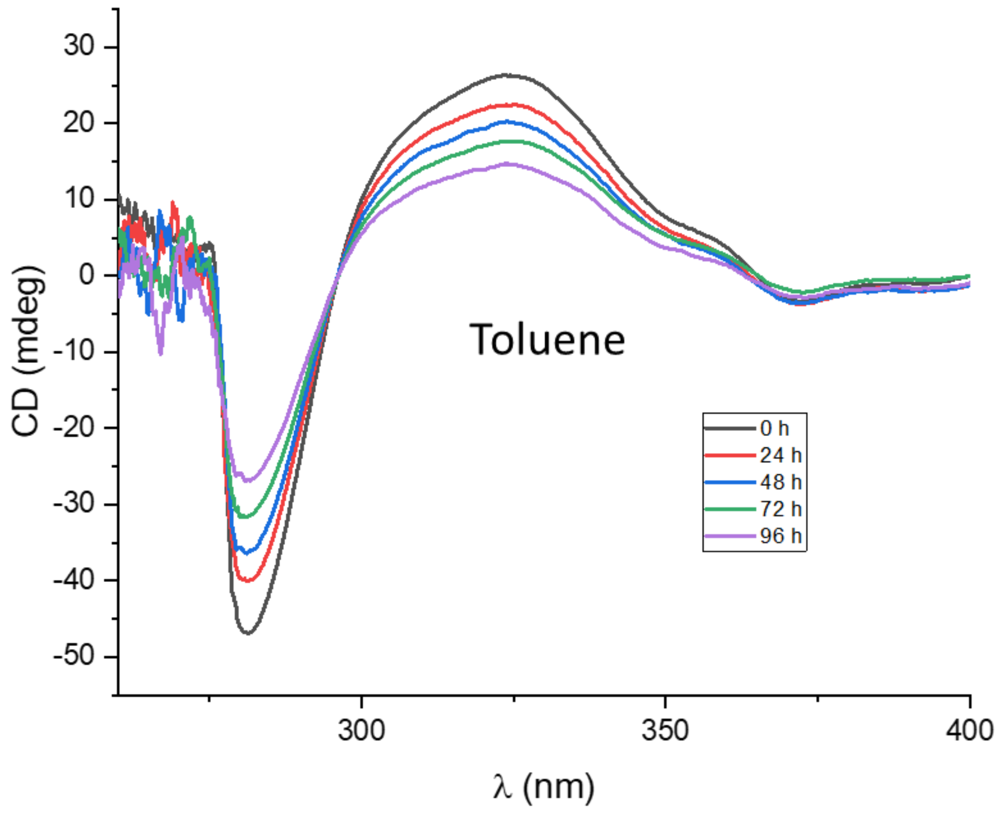

Figure S17: Time-course CD spectra for an enantiopure sample of **1a** ***MM*** (10 μM, ***MM*****:*****PP*** > 99:1 mol%) in toluene.

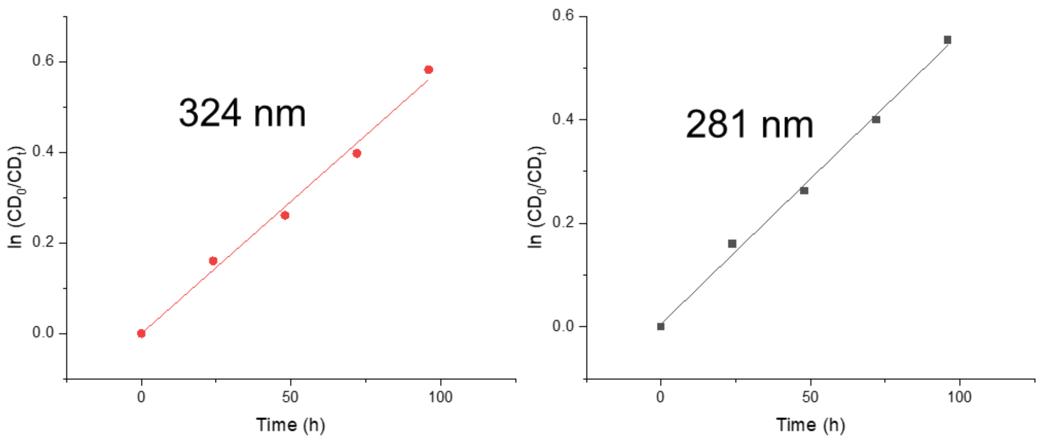

Wavelength	Slope (h^{-1})	Standard Error
324 nm	0.00584	3.19 x 10^{-4}
281 nm	0.00561	2.11 x 10^{-4}

Average slope (h^{-1})	Standard deviation
0.005725	1.15 x 10^{-4}

Figure S18: Plot and linear fit of $\ln\left({}^{CD_0}/_{CD_t}\right)$ against t at 281 nm and 324 nm for the time-course CD experiment measuring the rate of racemisation of macrocycle **1a** in toluene.

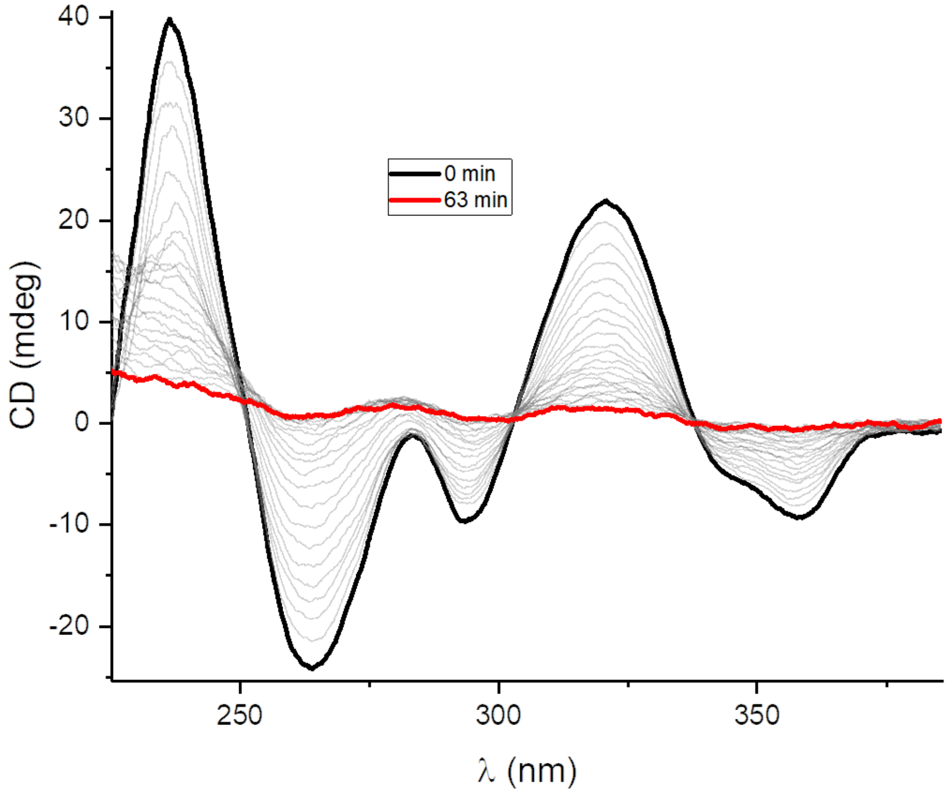

Figure S19: Time-course CD spectra for an enantiopure sample of **1a** ***MM*** (10 µM, ***MM*:*PP*** > 99:1 mol%) in DCM.

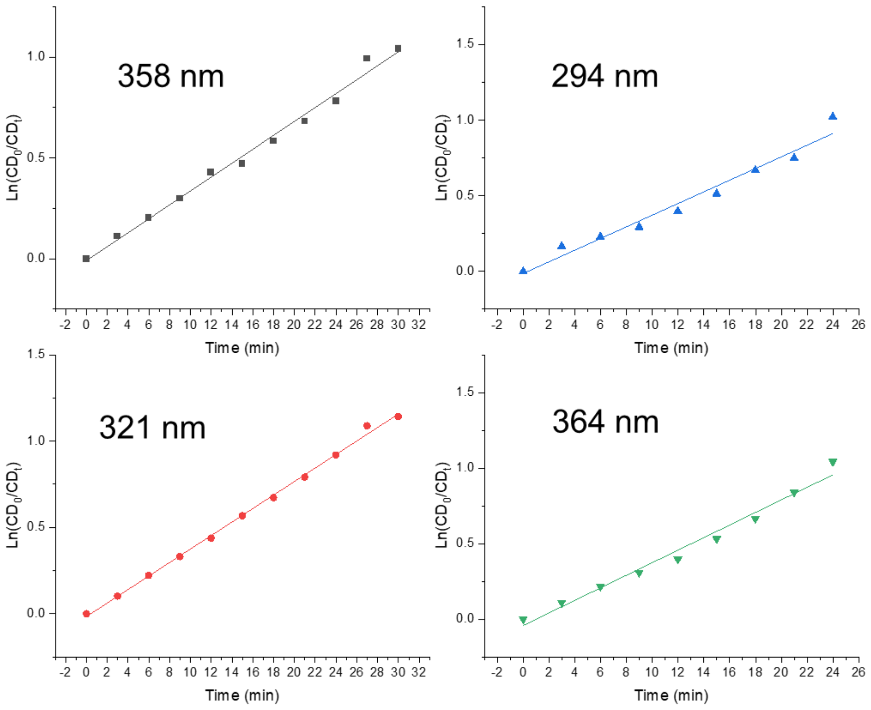

Wavelength	Slope (h^{-1})	Standard Error
358 nm	0.0345	0.0011
321 nm	0.03914	0.000635
294 nm	0.03855	0.0026
264 nm	0.0416	0.00221

Average slope (h^{-1})	Standard deviation
0.0384	0.0026

Figure S20: Plot and linear fit of $\ln\left({CD_0}/{CD_t}\right)$ against t at 358, 321, 294 and 264 nm for the time-course CD experiment measuring the rate of racemisation of macrocycle **1a** in DCM.

c) Circularly-polarised luminescence studies

CPL was measured with a home-built (modular) spectrometer. The excitation source was a broad band (200 – 1000 nm) laser- driven light source EQ 99 (Elliot Scientific). The excitation wavelength was selected by feeding the broadband light into an Acton SP-2155 monochromator (Princeton Instruments); the collimated light was focused into the sample cell (1 cm quartz cuvette). Sample PL emission was collected perpendicular to the excitation direction with a lens (f = 150 mm). The emission was fed through a photoelastic modulator (PEM) (Hinds Series II/FS42AA) and through a linear sheet polariser (Comar). The light was then focused into a second scanning monochromator (Acton SP- 2155) and subsequently on to a photomultiplier tube (PMT) (Hamamatsu H10723 series). The detection of the CPL signal was achieved using the field modulation lock-in technique. The electronic signal from the PMT was fed into a lock-in amplifier (Hinds Instruments Signaloc Model 2100). The reference signal for the lock-in detection was provided by the PEM control unit. The monchromators, PEM control unit and lock-in amplifier were interfaced to a desktop PC and controlled by a custom-written Labview graphic user interface. The lock-in amplifier provided two signals, an AC signal corresponding to (I_L- I_R) and a DC signal corresponding to (I_L + I_R) after background subtraction. The emission dissymmetry factor was therefore readily obtained from the experimental data, as 2 AC/DC.

Spectral calibration of the scanning monochromator was performed using a Hg-Ar calibration lamp (Ocean Optics). A correction factor for the wavelength dependence of the detection system was constructed using a calibrated lamp (Ocean Optics). The measured raw data was subsequently corrected using this correction factor. The validation of the CPL detection systems was achieved using light emitting diodes (LEDs) at various emission wavelengths. The LED was mounted in the sample holder and the light from the LED was fed through a broad band polarising filter and $\lambda/4$ plate (Ocean Optics) to generate circularly polarised light. Prior to all measurements, the $\lambda/4$ plate and a LED were used to set the phase of the lock-in amplifier correctly. The emission spectra were recorded with 0.5 nm step size and the slits of the detection monochromator were set to a slit width corresponding to a spectral resolution of 0.25 nm. CPL spectra (as well as total emission spectra) were obtained through an averaging procedure of several scans. The CPL spectra were smoothed using a shape-preserving Savitzky-Golay smoothing (polynomial order 5, window size 9 with reflection at the boundaries) to reduce the influence of noise and enhance visual appearance; all calculations were carried out using raw spectral data. Analysis of smoothed vs raw data was used to help to estimate the uncertainty in the stated g_{lum} factors, which was ±10%.

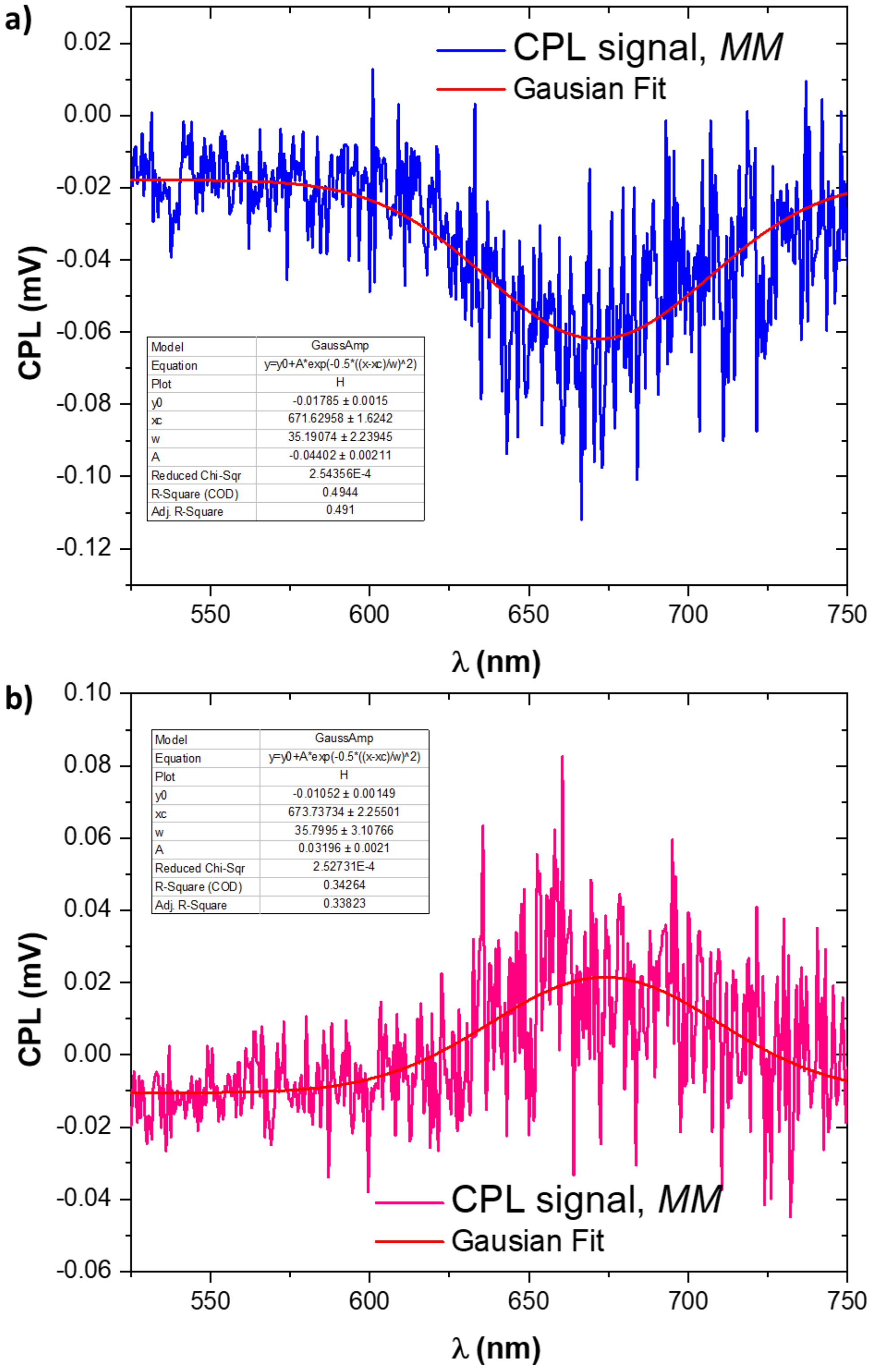

Figure S21: Raw and fitted CPL spectra of a) ***MM*** enantiomer (***MM*:*PP*** > 99:1 mol%) and b) ***PP*** enantiomer (***MM*:*PP*** = 12:88 mol%) of macrocycle **1a**.

d) Comparison with CPL emitters in the literature

Table S3 shows that macrocycle **1a** is at the upper end of the range of g_{lum} values ($g_{lum} = 10^{-2}$) for small organic molecules in solution (current highest is 10^{-1})[14-18] and the highest for discrete PDI emitters (current highest is 10^{-3}).[19] Furthermore, macrocycle **1a** exhibits the most red-shifted CPL spectrum (675 nm) of all small organic emitters reported to date.[19-21]

Table S3: A selection of the best performing small organic molecular CPL emitters in solution. The luminescence dissymmetry values (g_{lum}) and the wavelength of the emission maxima (λ_{em}) at which these were recorded are given.

Compound	Reference	g_{lum} (λ_{em} [nm])[a]	PDI-based
[4]Cyclo-2,8-chrysenylene	18	1.5×10^{-1} (443)	✗
Figure-eight-shaped [5]helicene dimer	22	1.5×10^{-2} (442)	✗
Ortho-oligo phenylene ethynylenes	23	5.5×10^{-2} (410)	✗
Perylene-based [4] rotaxane	24	2.1×10^{-2} (573)	✗
Pink box macrocycle **1a**	This work	1×10^{-2} (675)	✓
Tetrasubstituted [2.2]paracyclophane	25	1×10^{-2} (450)	✗
Azahelicene dimer	26	9×10^{-3} (536)	✗
Bis-BODIPY macrocycle	20	9×10^{-3} (663)	✗
π-extended [9]helicene	27	7×10^{-3} (532)	✗
Tetraarylated PDI	19	1×10^{-3} (655)	✓
PDI cyclohexane derivative	28	1×10^{-3} (610)	✓
Diketopyrrolopyrrole–helicene	21	9×10^{-4} (650)	✗

[a]Values are for discrete monomers in solution, as for macrocycle **1a**.

6. Photophysics

All steady state electronic absorption and emission spectra were recorded at a concentration of 10 µM (unless otherwise stated) at 298 K. For UV-Vis-NIR spectroscopy a Cary 5000 spectrophotometer was used, with a wavelength accuracy ≤ 0.08 nm and absorbance accuracy ≤ 0.01 Abs. For fluorescence spectroscopy a Jasco FP-8500 was used with emission and excitation wavelength accuracies ± 1.0 nm. The detector base sensitivity is 8500:1. Quartz cuvettes with 1 cm path length were used.

a) UV-vis-NIR absorption and emission spectra

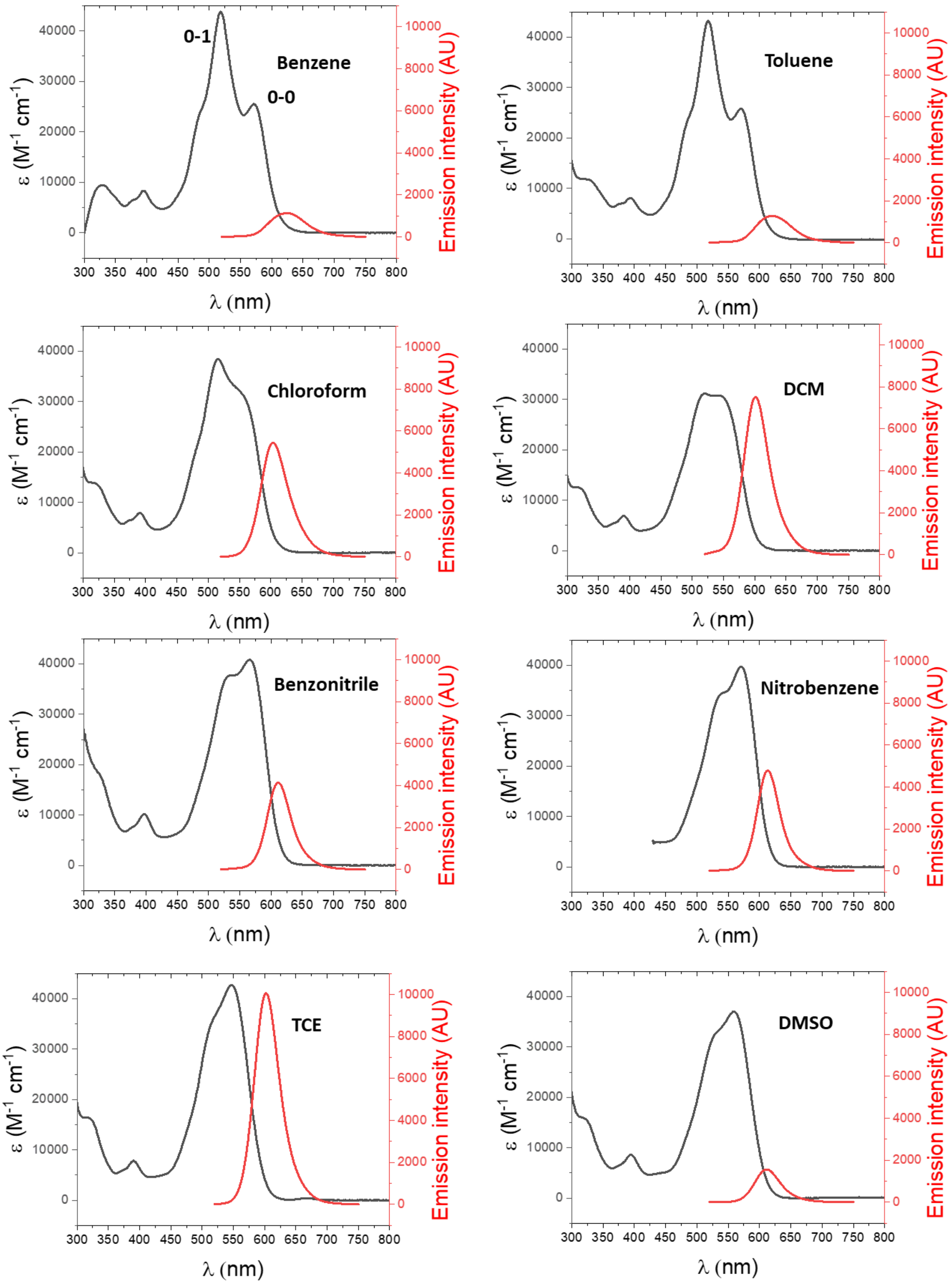

Figure S22: Normalised UV-vis absorption spectra and fluorescence emission spectra of macrocycle **1a** in selected solvents (10 μM). The 0-0 and 0-1 vibronic transition peaks are labelled in the absorption spectrum of benzene.

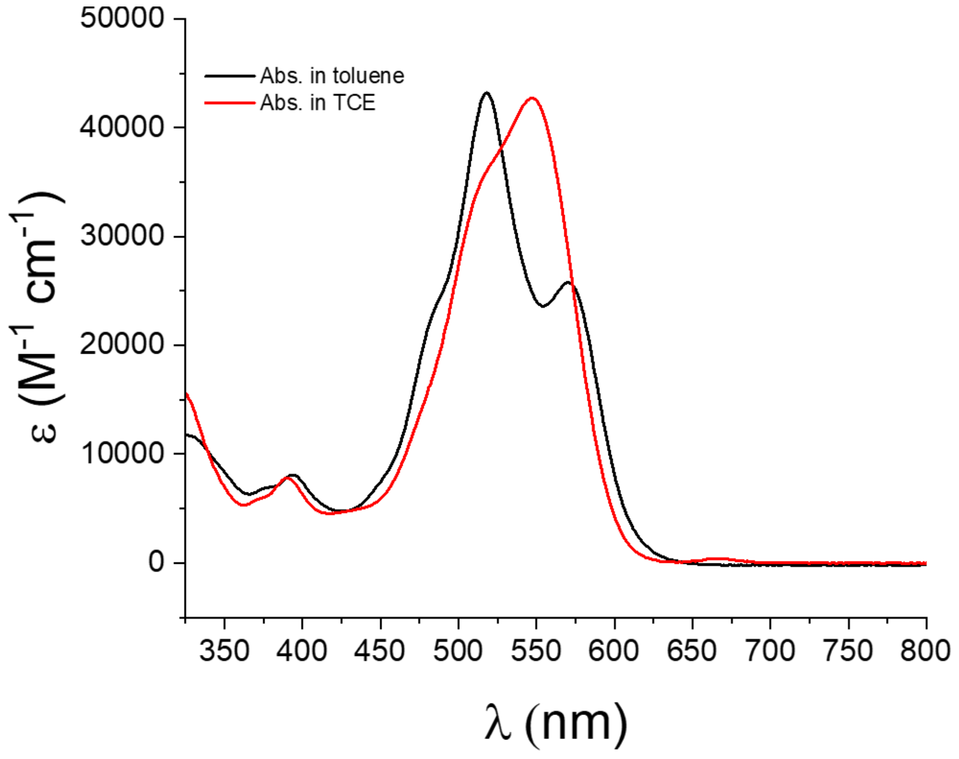

Figure S23: Normalised UV-vis absorption spectra of macrocycle **1a** in toluene and TCE (10 µM). Intramolecular H-type agreggation in toluene results in a reversal in the 0-0 and 0-1 vibronic peak intensities in the UV-vis spectrum, and in quenching of fluorescence emission (**Table S4**).

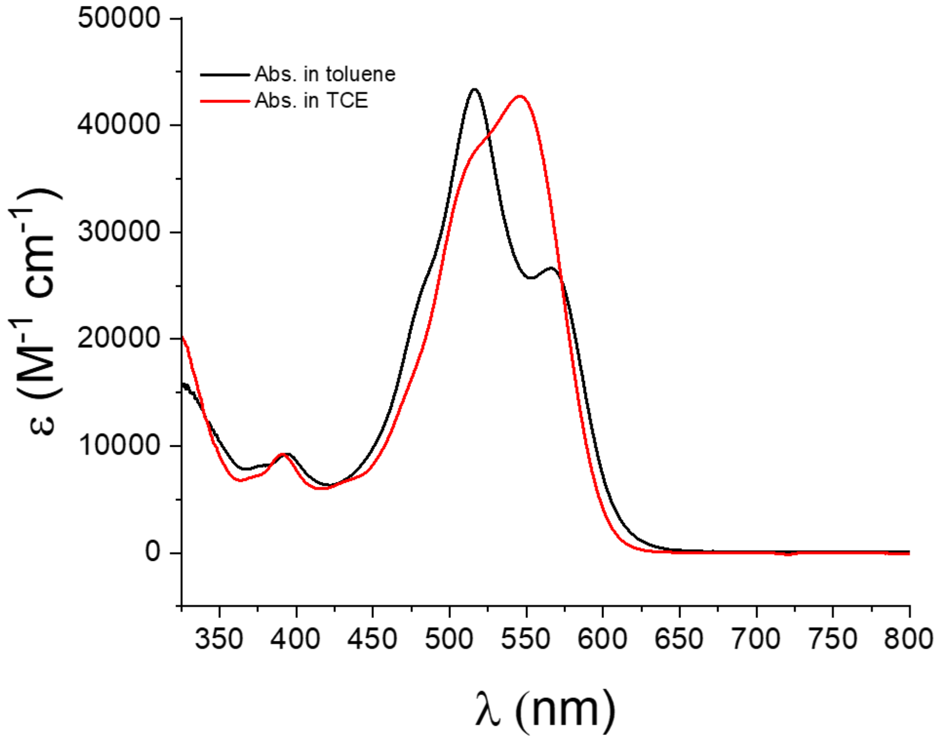

Figure S24: Normalised UV-vis absorption spectra of macrocycle **1b** in toluene and TCE (10 µM). These spectra are very similar to those of macrocycle **1a**.

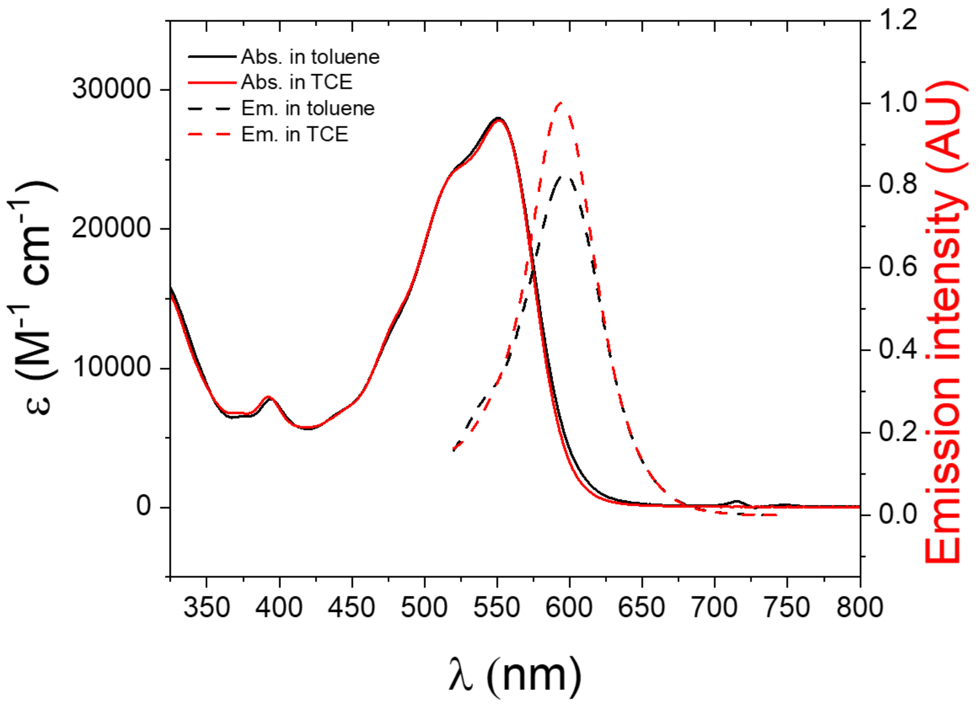

Figure S25: Normalised UV-vis absorption and fluorescence emission spectra of acyclic bis-triazole PDI **3a** in toluene and TCE (10 µM). The UV-vis spectra of **3a** have no solvent dependence, in contrast to macrocycles **1a,b**.

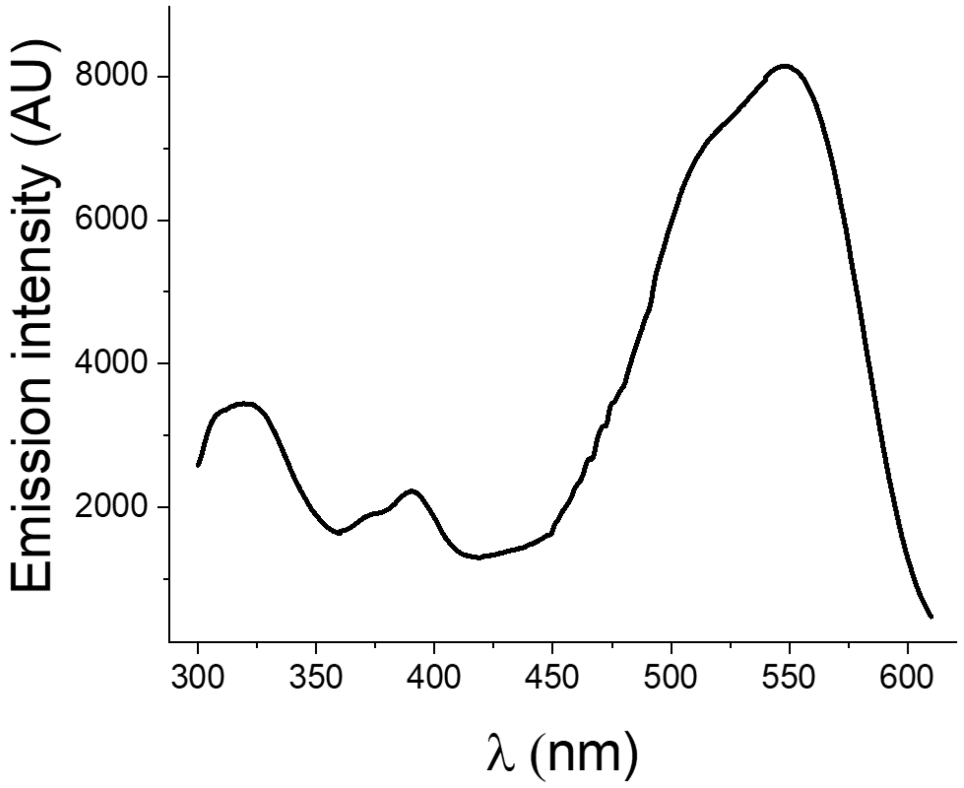

Figure S26: Excitation spectrum (λ_{em} = 620 nm) of macrocycle **1a** in TCE (10 µM).

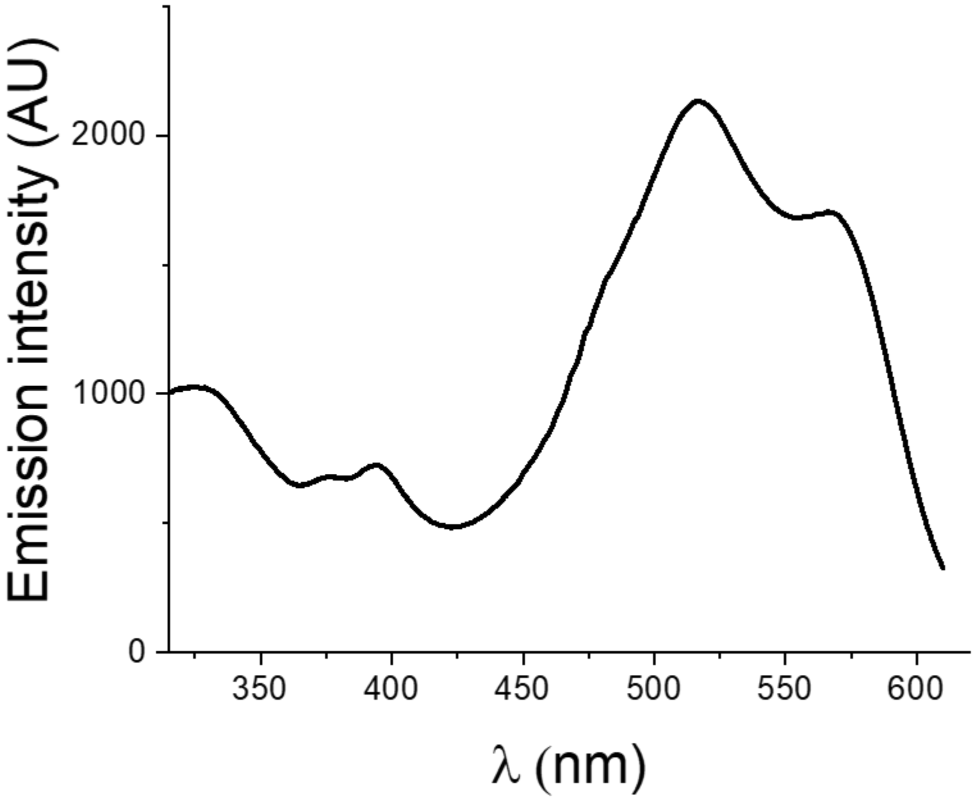

Figure S 27: Excitation spectrum (λ_{em} = 620 nm) of macrocycle **1a** in toluene (10 µM).

b) Beer-Lambert plots

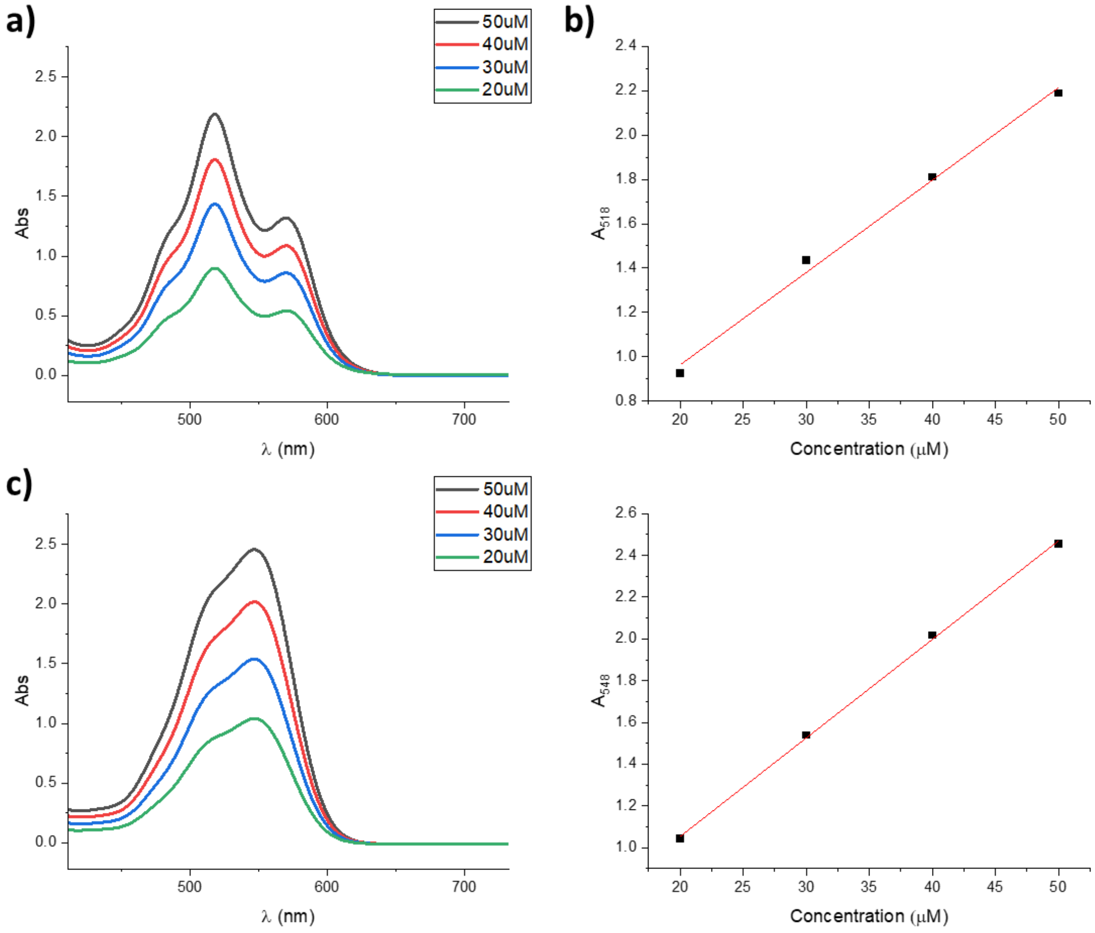

Figure S28: a) Absorption spectrum of macrocycle **1a** in toluene at different concentrations, up to Abs$_{max}$ for the instrument. **b)** The dependence of absorption (518 nm) on concentration for macrocycle **1a** in toluene is linear, showing Beer-Lambert behaviour. **c)** Absorption spectrum of macrocycle **1a** in TCE at different concentrations. **d)** The dependence of absorption (548 nm) on concentration for macrocycle **1a** in TCE is linear, showing Beer-Lambert behaviour.

e) Quantum yields

Absolute fluorescence quantum yields were obtained on an Edinburgh Instruments FLS920 steady-state spectrometer fitted with an integrating sphere. All samples were recorded at a 1 µM with a 7 - 8 nm excitation slit and 0.1 - 0.2 nm emission slit width. Experiments were carried out in solution using 1 cm path length quartz cuvettes with four transparent polished faces.

Table S4: Quantum yields for compounds **1a** and **3a** in toluene and TCE.

Compound	Solvent	Quantum Yield
**1a**	Toluene	0.48
	TCE	0.90
**3a**	Toluene	> 0.99
	TCE	> 0.99

7. Quantification of intramolecular H-type aggregation

To gain a deeper insight into the macrocycle-solvent interactions that promote intramolecular H-type aggregation, the UV-vis absorption spectrum of **1a** was recorded in a wide range of solvents. The $A_{0\text{-}0}$ / $A_{0\text{-}1}$ ratio was recorded for each solvent. From here a Gibbs free energy of intramolecular H-type aggregation (ΔG_{agg}) was determined for each solvent, following an adapted method used by Würthner and co-workers.[29] In this method, the following assumptions are made:

1. The $A_{0\text{-}0}$ / $A_{0\text{-}1}$ ratio in toluene corresponds to all molecules of macrocycle **1a** being in a state of full intramolecular H-type aggregation.
2. The $A_{0\text{-}0}$ / $A_{0\text{-}1}$ ratio in TCE corresponds to all molecules of macrocycle **1a** being in a state where there is no H-type aggregation.

These assumptions are validated by the fact the $\varepsilon_{0\text{-}0}$ / $\varepsilon_{0\text{-}1}$ ratio for macrocycle **1a** is minimised in toluene (0.58), while the ratio is maximised in TCE (1.19) and is the same as that of the monomeric PDI **3a** in either solvent. We note that the ***MP*** diastereomer is present as a minor species in chlorinated solvents (~10 mol%) and, from density functional theory calculations (**Section 9**), is not an H-type aggregate.

From these assumptions, the mole fraction α_u of fully unaggregated molecules can be estimated according to the following equation:

$$\alpha_u = \frac{\rho_a - \rho_{obs}}{\rho_a - \rho_u}$$

Where ρ_a is the $\varepsilon_{0\text{-}0}$ / $\varepsilon_{0\text{-}1}$ ratio of the fully H-type aggregated macrocycle in toluene, ρ_u is the $A_{0\text{-}0}$ / $A_{0\text{-}1}$ ratio of the non H-type aggregated macrocycle in TCE and ρ_{obs} is the observed $A_{0\text{-}0}$ / $A_{0\text{-}1}$ for a given solvent being investigated.[29]

From this, an equilibrium constant K_{eq} can be calculated as follows:

$$K_{eq} = \frac{C_a}{C_u} = \frac{1 - \alpha_u}{\alpha_u}$$

Where C_a and C_u are the concentrations of H-type aggregated and non H-type aggregated macrocycles respectively. Hence, a Gibbs free energy of intramolecular H-type aggregation (ΔG_{agg}) can be determined for each solvent according to:

$$\Delta G_{\mathrm{agg}} = -RT \ln K_{eq}$$

As toluene and TCE are the reference solvents for fully H-type aggregated and non H-type aggregated species respectively, they cannot be included in the ΔG plots as they represent asymptotes in the model.

Table S5: $A_{0\text{-}0}$ / $A_{0\text{-}1}$ ratios and corresponding ΔG_{agg} values for macrocycle **1a** in different solvents.

**Solvent**	**$A_{0\text{-}0}$ / $A_{0\text{-}1}$**	**ΔG_{agg} (kJ mol^{-1})**
Benzene	0.58	n/a
Toluene	0.58	n/a
tert-Butylbenzene	0.58	n/a
α,α,α-trifluorotoluene	0.59	-11.37
m-Xylene	0.62	-6.94
Fluorobenzene	0.62	-6.53
p-Xylene	0.62	-6.51
1:1 Chloroform:n-hexane	0.64	-5.76
o-Xylene	0.65	-5.22
Chlorobenzene	0.65	-5.12
Bromobenzene	0.73	-2.73
Acetonitrile	0.75	-2.39
Benzyl alcohol	0.78	-2.73
Chloroform	0.80	-1.40
Iodobenzene	0.82	-1.02
Trifluoroethanol	0.89	-0.035
Acetone	0.91	0.43
DCM	0.99	1.82
1,2-Dichlorobenzene	1.07	3.68
Benzonitrile	1.09	4.21
Benzaldehyde	1.09	4.47
DMSO	1.13	5.98
Nitrobenzene	1.16	8.00
TCE	1.19	n/a

The ΔG_{agg} of macrocycle **1a** for different solvents were plotted against various solvent scales (**Figure S29-36**). Good correlations are observed against scales that account for solvent polarity (ε, μ_a, Kirkwood-Onsager) or solvent polarity and polarizability (π*, Catalán SPP, χ_R), where Pearson's r = 0.8-0.9.[30] There is no correlation with solvent polarizability (α) or hydrogen bonding (β).

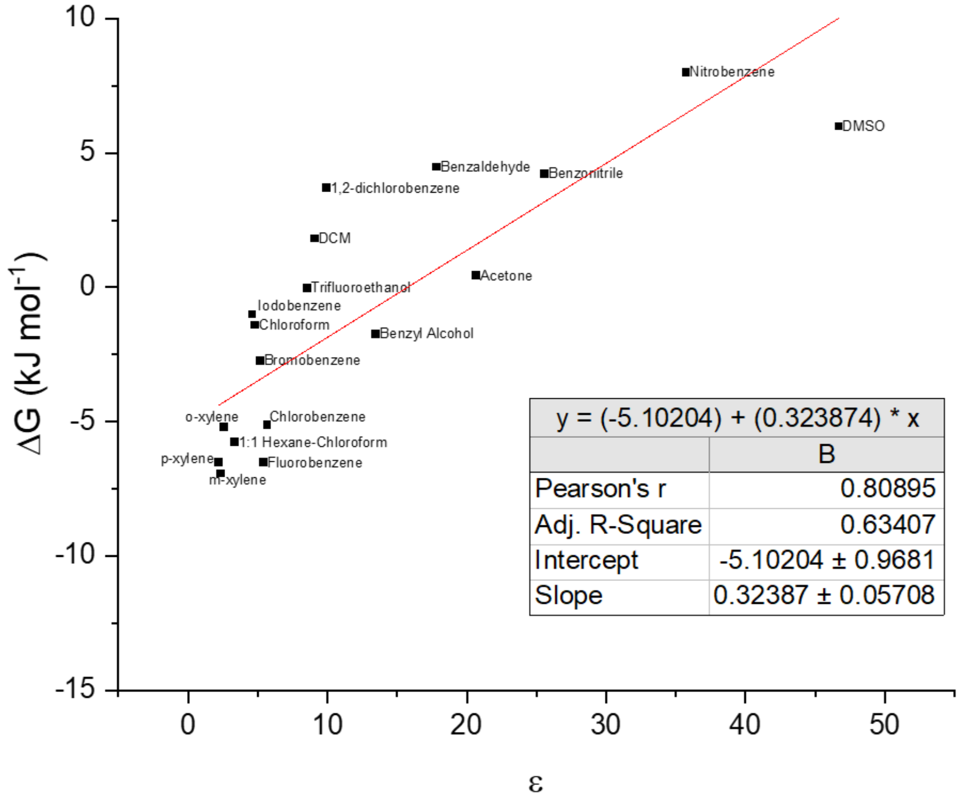

Figure S29: Plot of $\Delta \boldsymbol{G}_{agg}$ against dielectric constant ε of each solvent. The red line represents the linear regression fitting of the data.

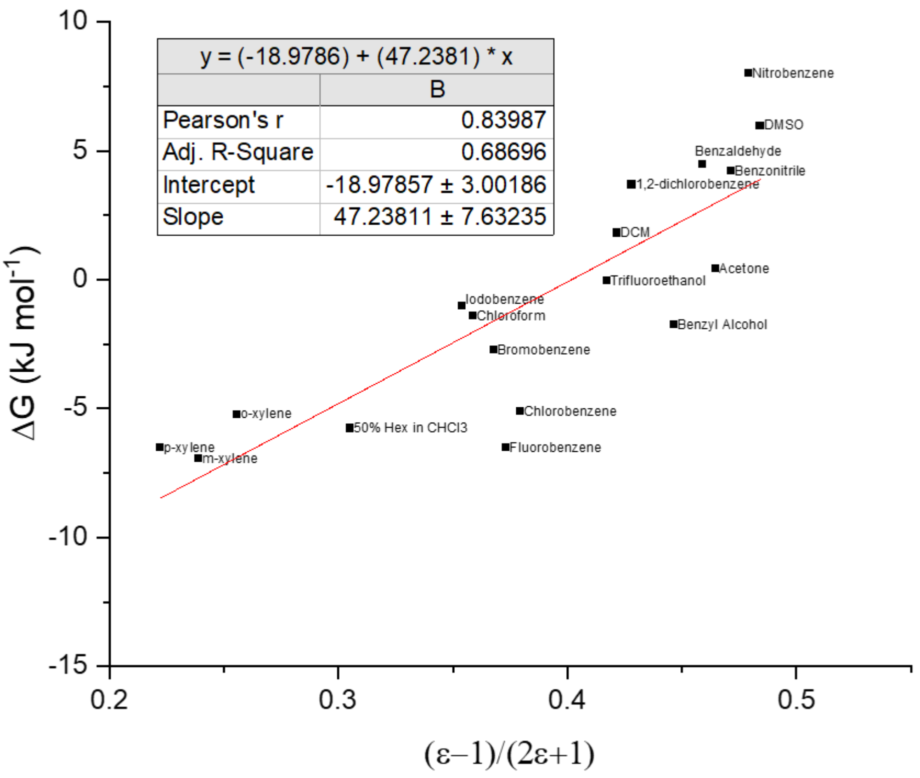

Figure S30: Plot of ΔG_{agg} against the Kirkwood-Onsager function $(\varepsilon - 1)(2\varepsilon + 1)$ for each solvent. The red line represents the linear regression fitting of the data.

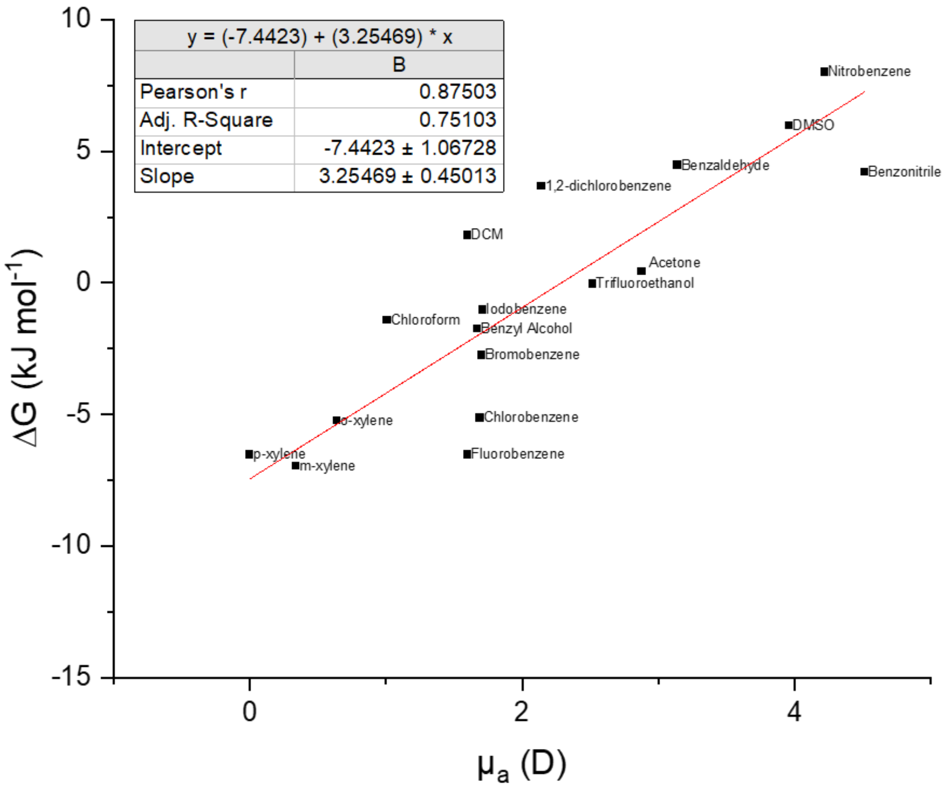

Figure S31: Plot of ΔG_{agg} against dipole moment μ_a of each solvent. The red line represents the linear regression fitting of the data.

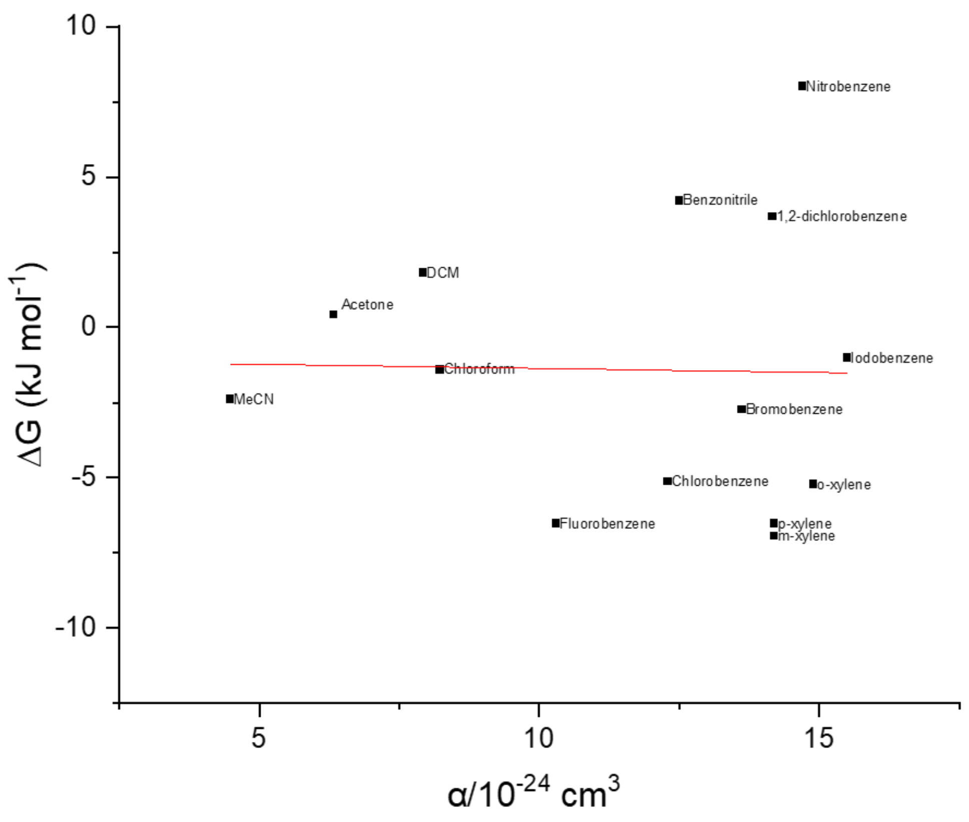

Figure S32: Plot of ΔG_{agg} against polarizability α of each solvent, showing no correlation.

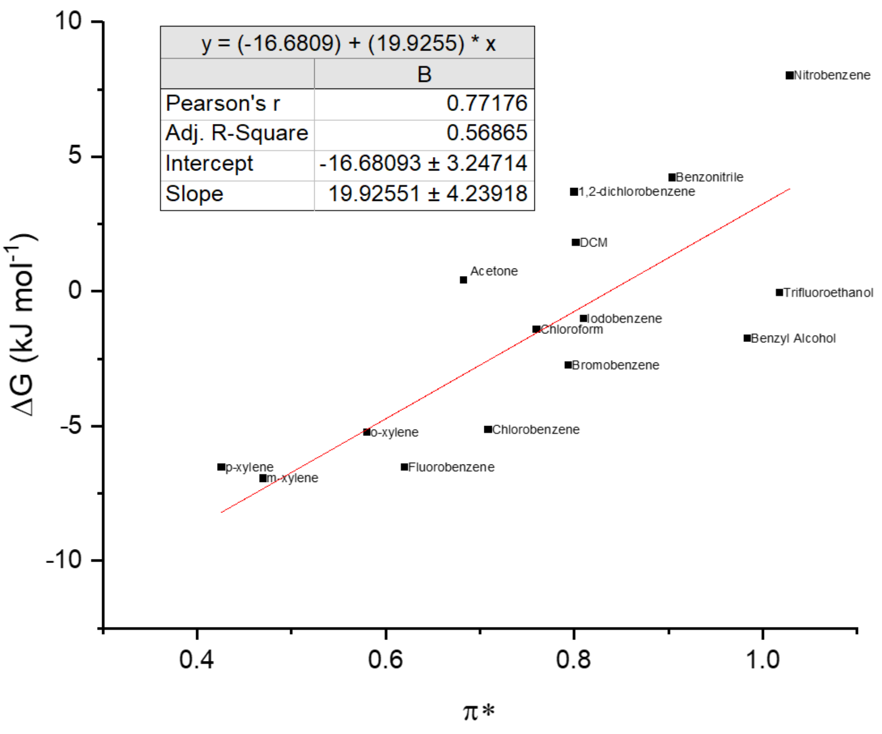

Figure S33: Plot of ΔG_{agg} against the empirical Kamlet-Taft π^*scale of solvent polarity/polarizability. [31] The red line represents the linear regression fitting of the data.

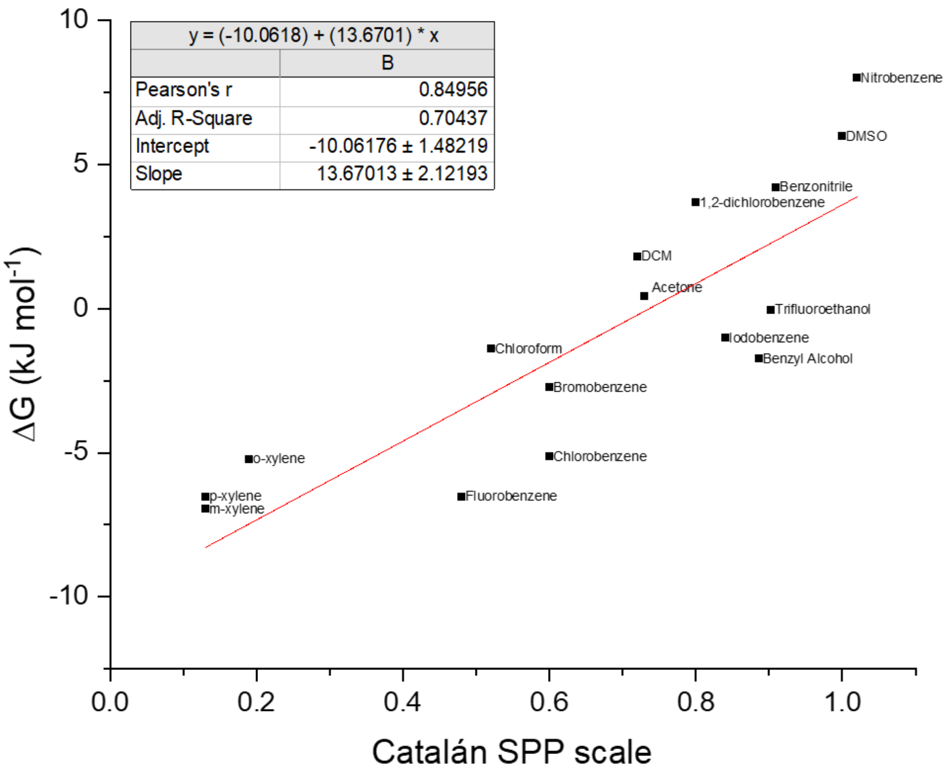

Figure S34: Plot of ΔG_{agg} against the empirical Catalán SPP scale of solvent polarity/polarizability. [32] The red line represents the linear regression fitting of the data.

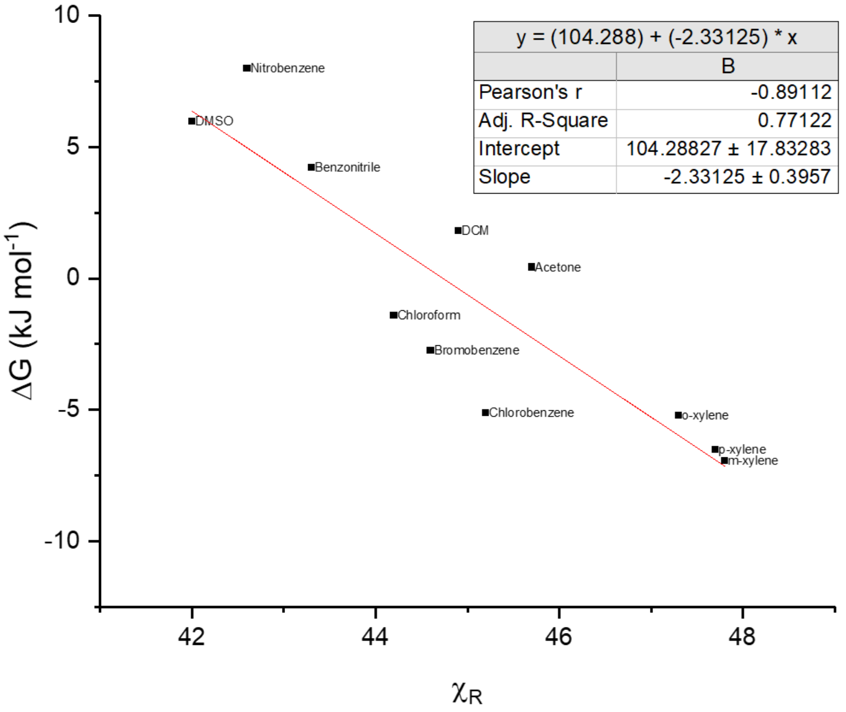

Figure S35: Plot of ΔG_{agg} against the empirical χ_R scale of solvent polarity/polarizability. The red line represents the linear regression fitting of the data. [33]

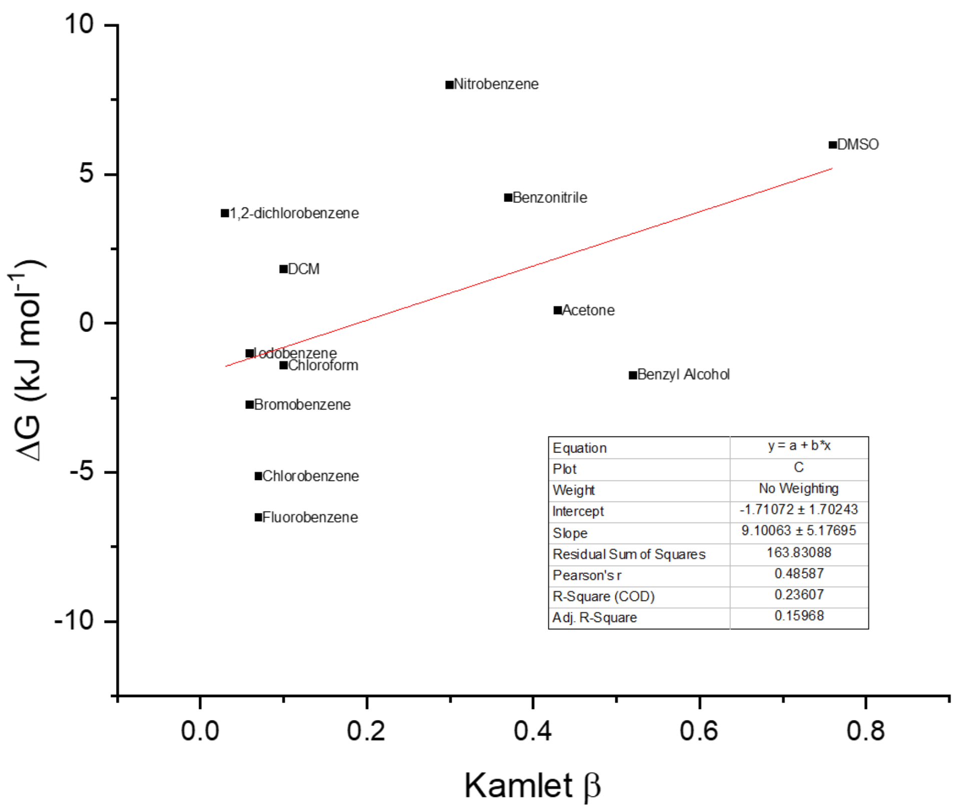

Figure S36: Plot of ΔG_{agg} against the Kamlet-Taft **β** scale of hydrogen bond acceptor strength.[34] No correlation is observed against **β.**

The UV-vis absorption spectrum of macrocycle **1a** was measured as the solvent composition was gradually changed from pure toluene to 97:3 (v/v) toluene:TCE, while keeping the concentration of **1a** constant (**Figure S37**). By monitoring the $A_{0\text{-}0}$ / $A_{0\text{-}1}$ ratio for each solvent composition (**Figure S38**), the free energy of intramolecular H-type aggregation (ΔG_{agg}) can be determined for different toluene:TCE solvent mixtures (**Figure S39**).

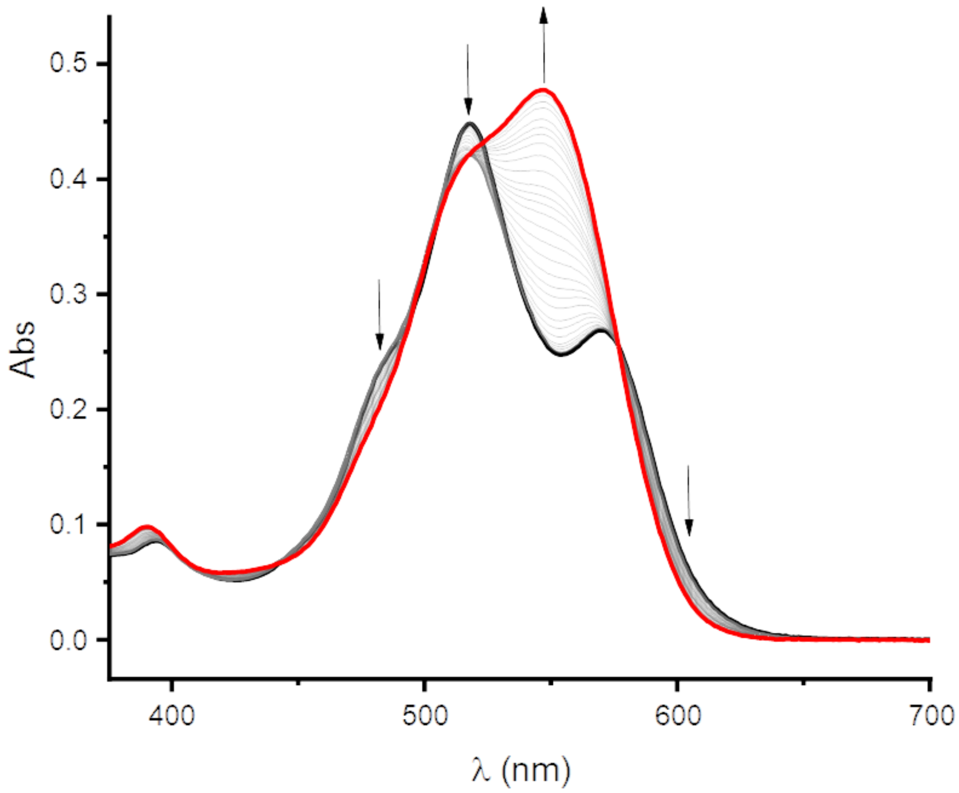

Figure S37: Change in the UV-vis absorption spectrum of a 10 µmol solution of macrocycle **1a** in toluene:TCE solvent mixtures (grey) going from pure toluene (black) to 97:3 (v/v) toluene:TCE (red). The arrows denote the direction of change as the proportion of TCE is increased.

The method used here is based on that developed by Moore and Ray to study the solvent-induced folding of phenylene ethynylene oligomers,[35] and used by Würthner and co-workers.[29] The equation below used to calculate ΔG_{agg} results in asymptotes at 0 % and 100 % TCE. However, plotting of the ΔG in the transition region against % TCE gives a straight-line relationship that can be extrapolated to obtain an estimate ΔG_{agg} for full intramolecular H-type aggregation in pure toluene (i.e., at 0% TCE) according to the equation:

$$\Delta G_{agg} = \Delta G(\mathrm{Tol}) + m[\mathrm{TCE}]$$

Where $\Delta G(\mathrm{Tol})$ is the free energy for full intramolecular H-type agreggation in pure toluene, [TCE] is the concentration of TCE in the solvent mixture, and m is the gradient of the plot. From **Figure S39**, this gives $\Delta G(\mathrm{Tol})$ = -11.17 ± 0.11 kJ mol^{-1}.

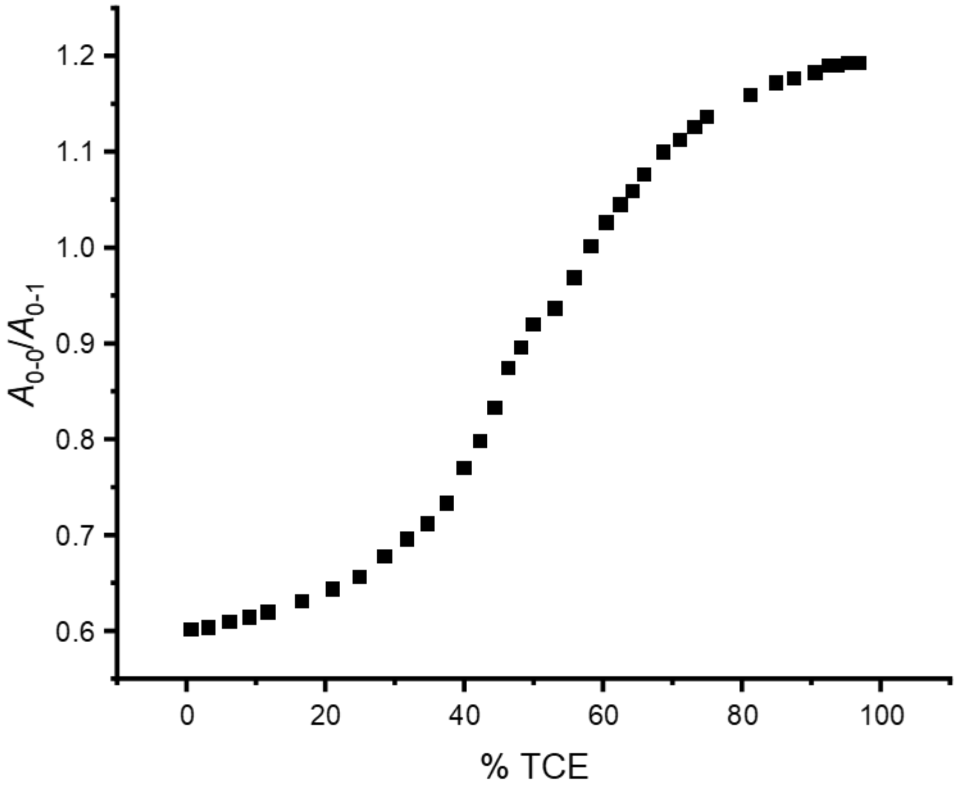

Figure S38: Change in A_{0-0} / A_{0-1} ratio in the absorption spectrum of compound **1a** in toluene:TCE mixture as the volume % of TCE is increased.

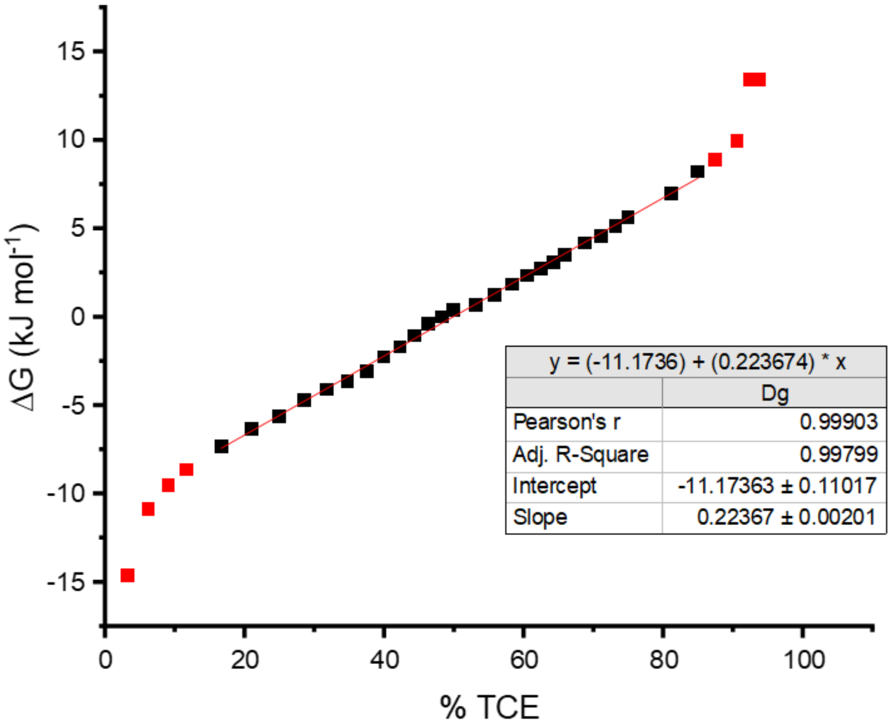

Figure S39: Plot of ΔG_{agg} of macrocycle **1a** (derived from the A_{0-0} / A_{0-1} ratio) for different toluene:TCE solvent mixtures. Data points in red are excluded from the linear regression fitting.

8. Electrochemistry

Electrochemical experiments were carried out in anhydrous degassed solvents (DCM or 1:1 toluene-DCM) using 0.1 M tetrabutylammonium tetrafluoroborate as the supporting electrolyte. The working electrode was a 3 mm glassy carbon electrode (polished with diamond slurry prior to use), a Pt counter electrode, and a Ag/AgCl reference. All cyclic voltammograms (CVs) were referenced to the Fc^{+}/Fc^{0} redox couple and recorded at a scan rate of 0.1 Vs^{-1}. An Autolab Interface 6 potentiostat was used for electrochemical measurements. The inherent instrument error is V: ± 0.2% (± 2 mV).

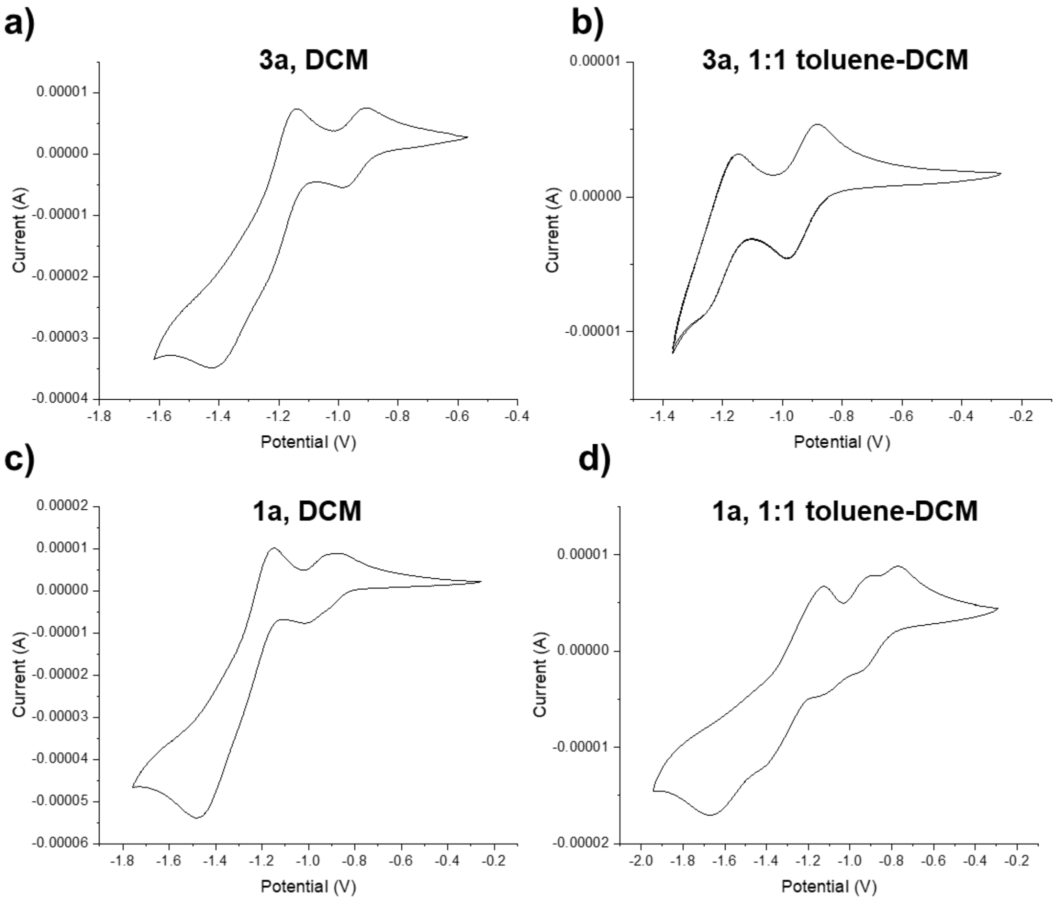

Figure S40: Cyclic voltammograms of a) Acyclic bis-triazole PDI **3a** in DCM, b) Acyclic bis-triazole PDI **3a** in 1:1 (v/v) toluene:DCM, c) macrocycle **1a** in DCM, d) macrocycle **1a** in 1:1 (v/v) toluene:DCM.

Table S6: Cyclic voltammetry data for macrocycle **1a** and acyclic PDI **3a**[a]

		**Redox Process, $E_{1/2}$ / V**			
	**Solvent**	**A**	**B**	**C**	**D**
Macrocycle **1a**	1:1 (v/v) toluene:DCM	−0.84	−1.02	−1.18	−1.70[b]
	DCM	−0.94		−1.31	
Acyclic PDI **3a**	1:1 (v/v) toluene:DCM	−0.94		−1.20	
	DCM	−0.94		−1.18	

[a]Recorded in the stated solvents containing 0.4 M [$^nBu_4N$][BF_4] as supporting electrolyte at ambient temperature, with potentials quoted at 0.10 V s^{-1} against $E_{1/2}$ Fc^+/Fc. [b]$E_{1/2}$ value could not be calculated so E_{ca} is given instead.

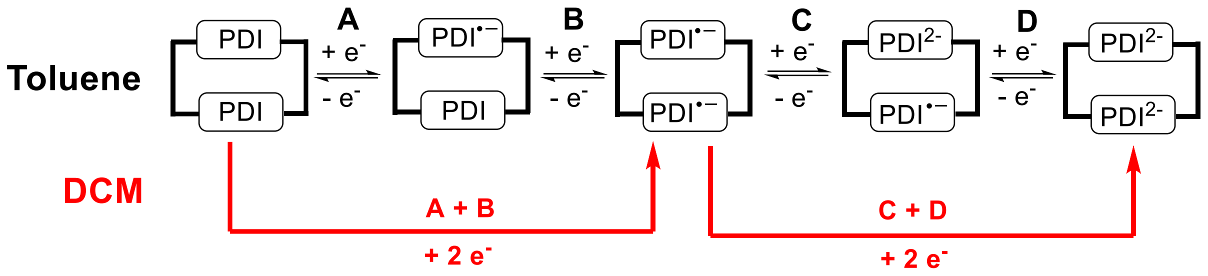

Figure S41: Proposed schematic of the redox processes of macrocycle **1a** in each solvent.

9. Density Functional Theory Calculations

a) Conformer search

Conformer searches in toluene and dichloromethane for a simplified model of macrocycle **1**, in which the imide-based alkyl chains (R groups) are replaced with methyl groups, were performed using the combination of the CREST code,[36] the GFN2-xTB semiempirical tight-binding method[37] and the analytical linearized Poisson–Boltzmann (ALPB) implicit solvation model.[38] The lowest energy conformers found using CREST were subsequently reoptimized by means of density functional theory using either the B97-3c composite scheme[39] or the combination of the PBE density functional,[40] the D4 dispersion correction method[41] and the def2-TZVP basis-set.[42] Solvation effects in the DFT calculations were described using either the COSMO[43] (toluene, dichloromethane) or COSMO-RS[44] (toluene, dichloromethane) implicit solvation models. All DFT calculations, including the time-dependent DFT and NMR calculations discussed below, are performed using Turbomole 7.5.[45-46]

An initial conformer search in the gas phase gave the lowest energy conformers **A-H** below. Conformations related by the simple rotation of the methyl group(s) at the imide position(s) were omitted. These structures are provided as .xyz files in the 'DFT structures' folder.

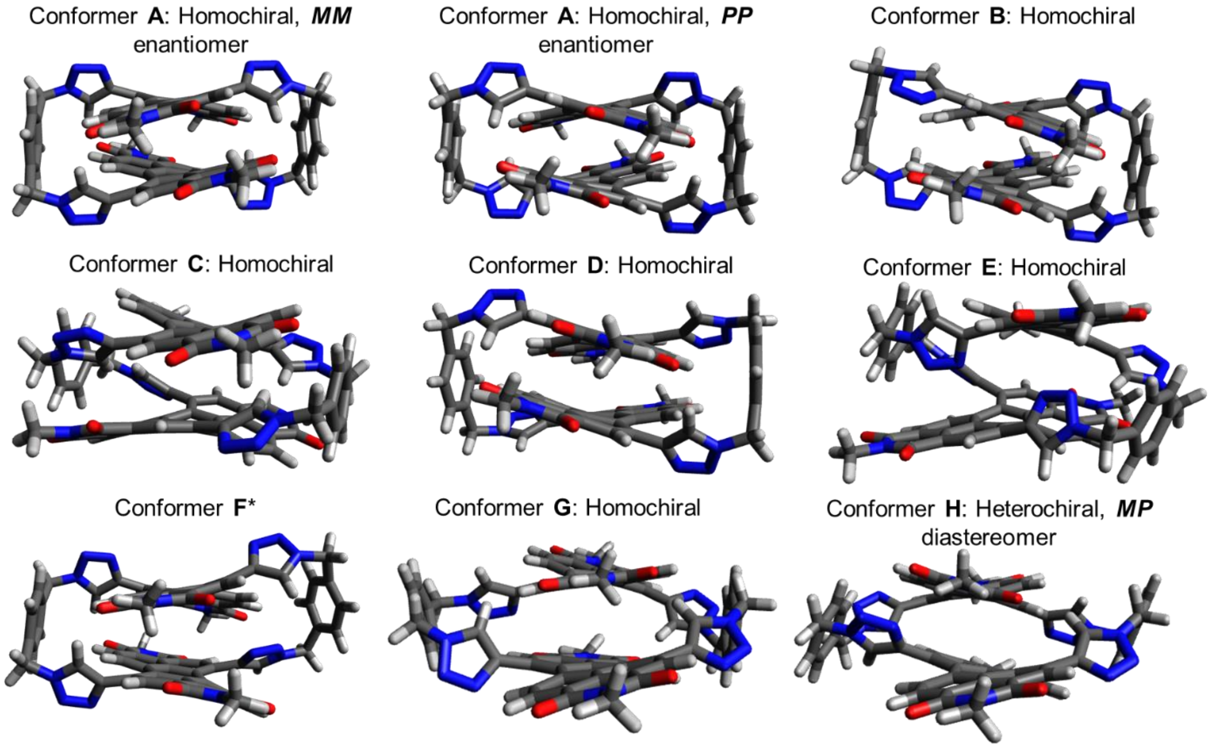

Figure S42: Lowest energy conformers **A-H** (gas phase). The homochiral/heterochiral labels refer to the relationship between the two axially chiral PDI units. Conformer **F** cannot be labelled in this way since the perylene core of the bottom PDI is pseudo planar. ***MM*** and ***PP*** assignments are included for conformer **A**, in agreement with the homochiral conformation of the macrocycle observed experimentally in toluene (**1a**) and the X-ray crystal structure (**1b**). The ***MP*** assignments is given to the heterochiral conformer **H**.

The relative energies of conformers **A-H** were obtained in the gas phase and in toluene and chlorinated solvents (**Tables S7-S8**). The energy landscape in chlorinated solvents predicted by density functional theory differs from that observed experimentally, most likely because our model does not account for intermolecular macrocycle–solvent hydrogen bonds, specifically impacting the energies of conformers **C** and **H**, in which intramolecular hydrogen bonds are broken.

Table S7: Relative energies in kJ/mol of conformers A-H in the gas phase and in different solvents described using different solvation models as calculated with B97-3c.

	gas phase	DCM	$CHCl_3$	Toluene	Toluene
	--	cosmo	cosmo-rs	cosmo	cosmo-rs
A	0	0	0	0	0
B	30	24	23	26	27
C	51	42	51	47	52
D	60	48	48	54	55
E	88	74	72	81	82
F	93	73	70	85	78
G	101	81	69	90	75
H	107	79	67	90	76

Table S8: Relative energies in kJ/mol of conformers **A-H** in the gas phase and in different solvents described using different solvation models as calculated with PBE-D4/def2-TZVP.

	gas phase	DCM	$CHCl_3$	Toluene	Toluene
	--	cosmo	cosmo-rs	cosmo	cosmo-rs
A	0	0	0	0	0
B	28	21	21	25	25
C	47	38	47	43	49
D	56	43	44	49	51
E	78	64	63	70	71
F	85	69	66	78	73
G	88	68	55	76	61
H	97	67	56	70	66

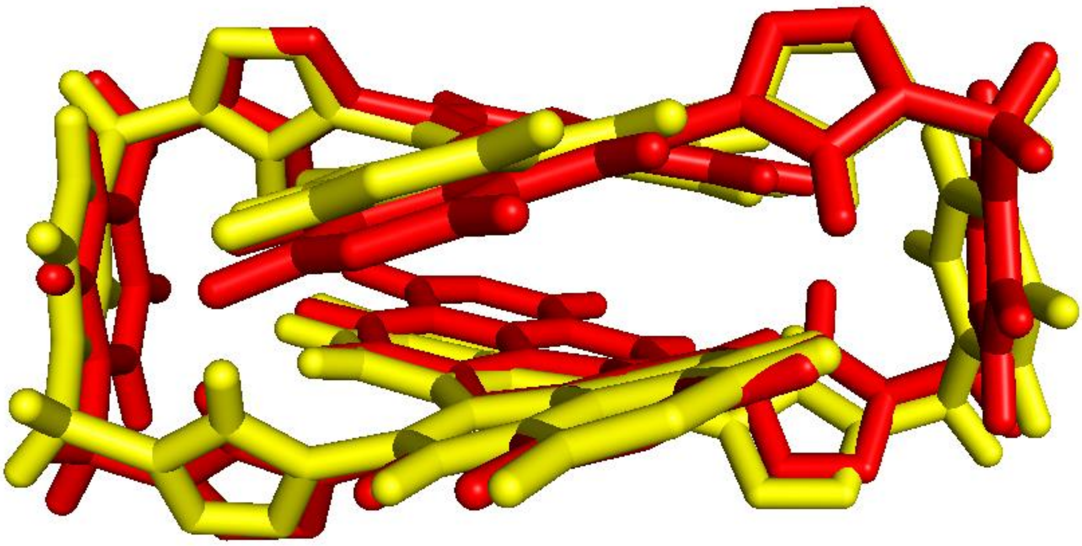

Figure S43: An overlay of the DFT predicted conformer **A** (red) and the same fragment of the X-ray crystal structure (yellow) of macrocycle **1**. The methyl group (DFT structure) or alkyl chains (crystal structure) have been omitted to aid with comparison.

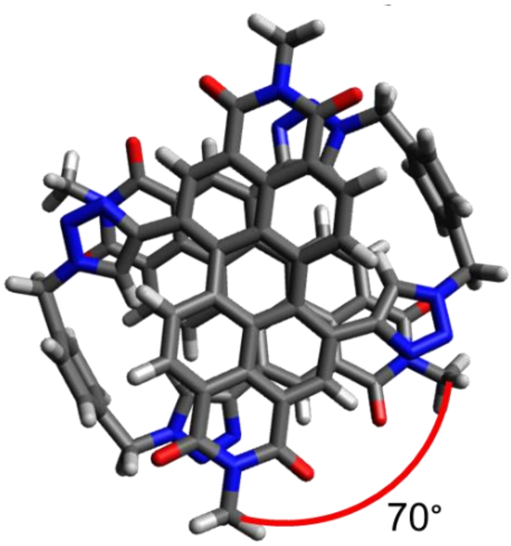

Figure S44: A top-down view of conformer **C** shows the PDI units are rotated by 70° relative to one another, which switches π–π interactions OFF. This contrasts with the 20° rotation in the H-type aggregated conformer **A** (π–π ON) as seen in the DFT structure (**Figure S43**) and X-ray crystal structure (**Figure 2c**).

b) Predicted UV-vis and CD spectra

Vertical excitation and circular dichroism spectra of the DFT optimised conformers were calculated by single point calculations on the B97-3c optimised structures using the combination of the ωB97x density functional[47] and the def2-TZVPP basis-set.[42]

Predicted UV-vis spectra

The lowest energy excitation (1) in the spectrum of **C** (i.e., π–π OFF, no H-type aggregation) has a greater intensity than the corresponding peak (1) in conformers **A** and **B** (i.e., π–π ON, H-type aggregate). Hence, DFT predicts a red-shifted peak for **C**, relative to **A** and **B**. This is consistent with the experimentally observed red shift of λ_{max} of macrocycle **1a** on going from toluene to chlorinated solvents.

Table S9 TD-ωB97x/def2-TZVPP predicted vertical excitation spectra conformer **A** in toluene (cosmo).

Exc.	excitation energy / eV	wavelength (nm)	oscillator strength
1	2.33	533	0.01202
2	2.60	477	0.68522
3	3.11	398	0.10038
4	3.16	392	0.00000

Table S10: TD-ωB97x/def2-TZVPP predicted vertical excitation spectra conformer **A** in DCM (cosmo).

Exc.	excitation energy / eV	wavelength (nm)	oscillator strength
1	2.31	537	0.01749
2	2.55	486	0.89341
3	3.11	399	0.10952
4	3.16	392	0.00001

Table S11: TD-ωB97x/def2-TZVPP predicted vertical excitation spectra conformer **B** in DCM (cosmo).

Exc.	excitation energy / eV	wavelength (nm)	oscillator strength
1	2.31	538	0.02390
2	2.53	491	0.85778
3	3.04	408	0.04706
4	3.15	393	0.04026

Table S12: TD-ωB97x/def2-TZVPP predicted vertical excitation spectra conformer **C** (no H-type aggregation) in DCM (cosmo).

Exc.	excitation energy / eV	wavelength (nm)	oscillator strength
1	2.42	513	0.11749
2	2.57	483	0.73674
3	3.37	368	0.02782
4	3.39	366	0.03303

Predicted CD spectra

Table S13: TD-ωB97x/def2-TZVPP predicted circular dichroism spectra for the ***MM*** and ***PP*** isomers of conformer A in toluene (cosmo).

	**Excitation energy / eV (wavelength / nm)**	**Rotary strength / 10^{-40} erg cm^3**	
		MM	***PP***
1	2.33 (533)	-223.26	223.35
2	2.60 (477)	292.31	-292.47
3	3.11 (398)	5.17	-5.08
4	3.16 (392)	-0.02	0.03
5	3.62 (342)	0.00	0.00
6	3.66 (339)	87.61	-87.62
7	3.78 (328)	-91.76	91.80
8	3.81 (326)	-136.50	136.45

The predicted CD spectra of each enantiomer of homochiral conformer A (***MM*** and ***PP***) were used to assign the experimental CD spectra of macrocycle **1a**.

c) Predicted ^{1}H NMR spectroscopy chemical shifts

^{1}H NMR spectroscopy chemical shifts of DFT optimised conformers were predicted using the Gauge-Including Atomic Orbitals method[42] and PBE-D4/def2-TZVP for structures optimised with the same functional and basis-set combination. The PDI protons $H_{a\text{-}c}$ were chosen because they are informative of PDI–PDI π–π stacking interactions.

The calculated ^{1}H NMR spectrum of conformer **A** (H-type aggregate) shows a good agreement with the ^{1}H NMR spectrum measured in toluene-d_8 (**Table S14**), and a poor agreement with the spectrum measured in TCE-d_2 (**Table S15**). Instead, the calculated spectrum for conformer **C** (no H-type aggregation) shows a good agreement with the spectrum measured in TCE-d_2 (**Table S16**).

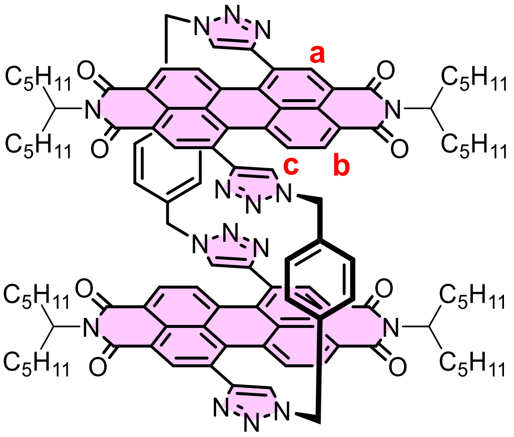

Macrocycle **1a**

Table S14: Calculated (conformer **A** of macrocycle **1**) and measured (macrocycle **1a**, 373 K) ^{1}H NMR spectroscopy chemical shifts of PDI protons $H_{a\text{-}c}$ in toluene-d_8 (relative to TMS), showing good agreement.

Proton	Calculated δ in toluene (ppm)	Measured δ in toluene-d_8 (ppm)	|Δδ|
a	8.0	8.1	0.1
b	7.7	7.6	0.1
c	5.7	5.7	0

Table S15: Calculated (conformer **A** of macrocycle **1**) and measured (macrocycle **1a**, 373 K) ^{1}H NMR spectroscopy chemical shifts of PDI protons $H_{a\text{-}c}$ in chlorinated solvent (relative to TMS), showing poor agreement.

Proton	Calculated δ in $CHCl_3$ (ppm)	Measured δ in TCE-d_2 (ppm)	|Δδ|
a	8.0	8.6	0.6
b	7.8	8.2	0.4
c	5.7	7.7	2.0

Table S16: Calculated (conformer **C** of macrocycle **1**) and measured (macrocycle **1a**, 373 K) ^{1}H NMR spectroscopy chemical shifts of PDI protons $H_{a\text{-}c}$ in chlorinated solvent (relative to TMS), showing good agreement.

Proton	Calculated δ in $CHCl_3$ (ppm)	Measured δ in TCE-d_2 (ppm)	|Δδ|
a	8.8	8.6	0.2
b	8.2	8.2	0
c	8.3	7.7	0.6

10. References

1. Chan, T. R.; Hilgraf, R.; Sharpless, K. B.; Fokin, V. V., Polytriazoles as Copper(I)-Stabilizing Ligands in Catalysis. *Organic Letters* **2004,** *6* (17), 2853-2855.
2. Schmidt, C. D.; Lang, N.; Jux, N.; Hirsch, A., A Facile Route to Water-Soluble Coronenes and Benzo[ghi]perylenes. *Chemistry – A European Journal* **2011,** *17* (19), 5289-5299.
3. Rajasingh, P.; Cohen, R.; Shirman, E.; Shimon, L. J. W.; Rybtchinski, B., Selective Bromination of Perylene Diimides under Mild Conditions. *The Journal of Organic Chemistry* **2007,** *72* (16), 5973-5979.
4. Perrin, C. L.; Dwyer, T. J., Application of two-dimensional NMR to kinetics of chemical exchange. *Chemical Reviews* **1990,** *90* (6), 935-967.
5. Farahani, N.; Zhu, K.; Loeb, S. J., Rigid, Bistable Molecular Shuttles Combining T-shaped Benzimidazolium and Y-shaped Imidazolium Recognition Sites. *ChemPhysChem* **2016,** *17* (12), 1875-1880.
6. Cobas, J. C.; Martin-Pastor, M., EXSYCalc Ver 1.0; Mestrelab Research, Coruña, Spain. *EXSYCalc Ver 1.0; Mestrelab Research, Coruña, Spain*

2007.
7. Atkins, P. W.; De Paula, J., *Atkins' Physical chemistry*. 10th ed. ed.; Oxford University Press: Oxford, 2014.
8. Beilsten-Edmands, J.; Winter, G.; Gildea, R.; Parkhurst, J.; Waterman, D.; Evans, G., Scaling diffraction data in the DIALS software package: algorithms and new approaches for multi-crystal scaling. *Acta Crystallographica Section D* **2020,** *76* (4), 385-399.
9. Winter, G., xia2: an expert system for macromolecular crystallography data reduction. *Journal of Applied Crystallography* **2010,** *43* (1), 186-190.
10. Winter, G.; Waterman, D. G.; Parkhurst, J. M.; Brewster, A. S.; Gildea, R. J.; Gerstel, M.; Fuentes-Montero, L.; Vollmar, M.; Michels-Clark, T.; Young, I. D.; Sauter, N. K.; Evans, G., DIALS: implementation and evaluation of a new integration package. *Acta Crystallographica Section D* **2018,** *74* (2), 85-97.
11. Sheldrick, G., SHELXT - Integrated space-group and crystal-structure determination. *Acta Crystallographica Section A* **2015,** *71* (1), 3-8.
12. Dolomanov, O. V.; Bourhis, L. J.; Gildea, R. J.; Howard, J. A. K.; Puschmann, H., OLEX2: a complete structure solution, refinement and analysis program. *Journal of Applied Crystallography* **2009,** *42* (2), 339-341.
13. Rickhaus, M.; Jundt, L.; Mayor, M., Determining Inversion Barriers in Atrop- isomers – A Tutorial for Organic Chemists. *CHIMIA* **2016,** *70* (3), 192.
14. Sánchez-Carnerero, E. M.; Agarrabeitia, A. R.; Moreno, F.; Maroto, B. L.; Muller, G.; Ortiz, M. J.; de la Moya, S., Circularly Polarized Luminescence from Simple Organic Molecules. *Chemistry – A European Journal* **2015,** *21* (39), 13488-13500.
15. Zhao, J.; Xing, P., Regulation of Circularly Polarized Luminescence in Multicomponent Supramolecular Coassemblies. *ChemPhotoChem* **2022,** *6* (1), e202100124.
16. Hasegawa, M.; Nojima, Y.; Mazaki, Y., Circularly Polarized Luminescence in Chiral π-Conjugated Macrocycles. *ChemPhotoChem* **2021,** *5* (12), 1042-1058.
17. Ma, J.-L.; Peng, Q.; Zhao, C.-H., Circularly Polarized Luminescence Switching in Small Organic Molecules. *Chemistry – A European Journal* **2019,** *25* (68), 15441-15454.
18. Sato, S.; Yoshii, A.; Takahashi, S.; Furumi, S.; Takeuchi, M.; Isobe, H., Chiral intertwined spirals and magnetic transition dipole moments dictated by cylinder helicity. *Proceedings of the National Academy of Sciences* **2017,** *114* (50), 13097-13101.
19. Renner, R.; Mahlmeister, B.; Anhalt, O.; Stolte, M.; Würthner, F., Chiral Perylene Bisimide Dyes by Interlocked Arene Substituents in the Bay Area. *Chemistry – A European Journal* **2021,** *27* (46), 11997-12006.

20. Saikawa, M.; Nakamura, T.; Uchida, J.; Yamamura, M.; Nabeshima, T., Synthesis of figure-of-eight helical bisBODIPY macrocycles and their chiroptical properties. *Chemical Communications* **2016,** *52* (71), 10727-10730.
21. Dhbaibi, K.; Favereau, L.; Srebro-Hooper, M.; Jean, M.; Vanthuyne, N.; Zinna, F.; Jamoussi, B.; Di Bari, L.; Autschbach, J.; Crassous, J., Exciton coupling in diketopyrrolopyrrole–helicene derivatives leads to red and near-infrared circularly polarized luminescence. *Chemical Science* **2018,** *9* (3), 735-742.
22. Kubo, H.; Shimizu, D.; Hirose, T.; Matsuda, K., Circularly Polarized Luminescence Designed from Molecular Orbitals: A Figure-Eight-Shaped [5]Helicene Dimer with D2 Symmetry. *Organic Letters* **2020,** *22* (23), 9276-9281.
23. Reine, P.; Campaña, A. G.; Alvarez de Cienfuegos, L.; Blanco, V.; Abbate, S.; Mota, A. J.; Longhi, G.; Miguel, D.; Cuerva, J. M., Chiral double stapled o-OPEs with intense circularly polarized luminescence. *Chemical Communications* **2019,** *55* (72), 10685-10688.
24. Hayashi, K.; Miyaoka, Y.; Ohishi, Y.; Uchida, T.-a.; Iwamura, M.; Nozaki, K.; Inouye, M., Observation of Circularly Polarized Luminescence of the Excimer from Two Perylene Cores in the Form of [4]Rotaxane. *Chem. - Eur. J.* **2018,** *24* (55), 14613-14616.
25. Morisaki, Y.; Gon, M.; Sasamori, T.; Tokitoh, N.; Chujo, Y., Planar Chiral Tetrasubstituted [2.2]Paracyclophane: Optical Resolution and Functionalization. *Journal of the American Chemical Society* **2014,** *136* (9), 3350-3353.
26. Ushiyama, A.; Hiroto, S.; Yuasa, J.; Kawai, T.; Shinokubo, H., Synthesis of a figure-eight azahelicene dimer with high emission and CPL properties. *Organic Chemistry Frontiers* **2017,** *4* (5), 664-667.
27. Qiu, Z.; Ju, C.-W.; Frédéric, L.; Hu, Y.; Schollmeyer, D.; Pieters, G.; Müllen, K.; Narita, A., Amplification of Dissymmetry Factors in π-Extended [7]- and [9]Helicenes. *Journal of the American Chemical Society* **2021,** *143* (12), 4661-4667.
28. Reine, P.; Ortuño, A. M.; Mariz, I. F. A.; Ribagorda, M.; Cuerva, J. M.; Campaña, A. G.; Maçôas, E.; Miguel, D., Simple Perylene Diimide Cyclohexane Derivative With Combined CPL and TPA Properties. *Frontiers in Chemistry* **2020,** *8*.
29. Spenst, P.; Young, R. M.; Phelan, B. T.; Keller, M.; Dostál, J.; Brixner, T.; Wasielewski, M. R.; Würthner, F., Solvent-Templated Folding of Perylene Bisimide Macrocycles into Coiled Double-String Ropes with Solvent-Sensitive Optical Signatures. *Journal of the American Chemical Society* **2017,** *139* (5), 2014-2021.
30. Lan, Y.; Corradini, M. G.; Liu, X.; May, T. E.; Borondics, F.; Weiss, R. G.; Rogers, M. A., Comparing and Correlating Solubility Parameters Governing the Self-Assembly of Molecular Gels Using 1,3:2,4-Dibenzylidene Sorbitol as the Gelator. *Langmuir* **2014,** *30* (47), 14128-14142.
31. Kamlet, M. J.; Abboud, J. L.; Taft, R. W., The solvatochromic comparison method. 6. The .pi.* scale of solvent polarities. *Journal of the American Chemical Society* **1977,** *99* (18), 6027-6038.
32. Catalán, J.; López, V.; Pérez, P.; Martin-Villamil, R.; Rodríguez, J.-G., Progress towards a generalized solvent polarity scale: The solvatochromism of 2-(dimethylamino)-7-nitrofluorene and its homomorph 2-fluoro-7-nitrofluorene. *Liebigs Annalen* **1995,** *1995* (2), 241-252.
33. Brooker, L. G. S.; Craig, A. C.; Heseltine, D. W.; Jenkins, P. W.; Lincoln, L. L., Color and Constitution. XIII.1 Merocyanines as Solvent Property Indicators2. *Journal of the American Chemical Society* **1965,** *87* (11), 2443-2450.
34. Kamlet, M. J.; Taft, R. W., The solvatochromic comparison method. I. The .beta.-scale of solvent hydrogen-bond acceptor (HBA) basicities. *Journal of the American Chemical Society* **1976,** *98* (2), 377-383.
35. Moore, J. S.; Ray, C. R., Supramolecular Organization of Foldable Phenylene Ethynylene Oligomers. In *Poly(arylene etynylene)s: From Synthesis to Application*, Weder, C., Ed. Springer Berlin Heidelberg: Berlin, Heidelberg, 2005; pp 91-149.
36. Pracht, P.; Bohle, F.; Grimme, S., Automated exploration of the low-energy chemical space with fast quantum chemical methods. *Physical Chemistry Chemical Physics* **2020,** *22* (14), 7169-7192.

37. Bannwarth, C.; Ehlert, S.; Grimme, S., GFN2-xTB—An Accurate and Broadly Parametrized Self-Consistent Tight-Binding Quantum Chemical Method with Multipole Electrostatics and Density-Dependent Dispersion Contributions. *Journal of Chemical Theory and Computation* **2019,** *15* (3), 1652-1671.
38. Ehlert, S.; Stahn, M.; Spicher, S.; Grimme, S., Robust and Efficient Implicit Solvation Model for Fast Semiempirical Methods. *Journal of Chemical Theory and Computation* **2021,** *17* (7), 4250-4261.
39. Brandenburg, J. G.; Bannwarth, C.; Hansen, A.; Grimme, S., B97-3c: A revised low-cost variant of the B97-D density functional method. *The Journal of Chemical Physics* **2018,** *148* (6), 064104.
40. Perdew, J. P.; Burke, K.; Ernzerhof, M., Generalized Gradient Approximation Made Simple. *Physical Review Letters* **1996,** *77* (18), 3865-3868.
41. Caldeweyher, E.; Bannwarth, C.; Grimme, S., Extension of the D3 dispersion coefficient model. *The Journal of Chemical Physics* **2017,** *147* (3), 034112.
42. Weigend, F.; Ahlrichs, R., Balanced basis sets of split valence, triple zeta valence and quadruple zeta valence quality for H to Rn: Design and assessment of accuracy. *Physical Chemistry Chemical Physics* **2005,** *7* (18), 3297-3305.
43. Klamt, A.; Schüürmann, G., COSMO: a new approach to dielectric screening in solvents with explicit expressions for the screening energy and its gradient. *Journal of the Chemical Society, Perkin Transactions 2* **1993,** (5), 799-805.
44. Sinnecker, S.; Rajendran, A.; Klamt, A.; Diedenhofen, M.; Neese, F., Calculation of Solvent Shifts on Electronic g-Tensors with the Conductor-Like Screening Model (COSMO) and Its Self-Consistent Generalization to Real Solvents (Direct COSMO-RS). *The Journal of Physical Chemistry A* **2006,** *110* (6), 2235-2245.
45. Furche, F.; Ahlrichs, R.; Hättig, C.; Klopper, W.; Sierka, M.; Weigend, F., Turbomole. *WIREs Computational Molecular Science* **2014,** *4* (2), 91-100.
46. Balasubramani, S. G.; Chen, G. P.; Coriani, S.; Diedenhofen, M.; Frank, M. S.; Franzke, Y. J.; Furche, F.; Grotjahn, R.; Harding, M. E.; Hättig, C.; Hellweg, A.; Helmich-Paris, B.; Holzer, C.; Huniar, U.; Kaupp, M.; Khah, A. M.; Khani, S. K.; Müller, T.; Mack, F.; Nguyen, B. D.; Parker, S. M.; Perlt, E.; Rappoport, D.; Reiter, K.; Roy, S.; Rückert, M.; Schmitz, G.; Sierka, M.; Tapavicza, E.; Tew, D. P.; Wüllen, C. v.; Voora, V. K.; Weigend, F.; Wodyński, A.; Yu, J. M., TURBOMOLE: Modular program suite for ab initio quantum-chemical and condensed-matter simulations. *The Journal of Chemical Physics* **2020,** *152* (18), 184107.
47. Chai, J.-D.; Head-Gordon, M., Systematic optimization of long-range corrected hybrid density functionals. *The Journal of Chemical Physics* **2008,** *128* (8), 084106.
